# Microarray analysis of toxicogenomic effects of Ortho-phenylphenol in *Staphylococcus aureus*

**DOI:** 10.1186/1471-2164-9-411

**Published:** 2008-09-15

**Authors:** Hyeung-Jin Jang, Chantal Nde, Freshteh Toghrol, William E Bentley

**Affiliations:** 1 Center for Biosystems Research, University of Maryland Biotechnology Institute, College Park, Maryland 20742, USA; 2 Microarray Research Laboratory, Biological and Economic Analysis Division, Office of Pesticide Programs, U. S. Environmental Protection Agency, Fort Meade, Maryland 20755, USA

## Abstract

**Background:**

*Staphylococcus aureus *(*S. aureus*), is responsible for many infectious diseases, ranging from benign skin infections to life-threatening endocarditis and toxic shock syndrome. Ortho-phenylphenol (OPP) is an antimicrobial agent and an active ingredient of EPA-registered disinfectants with wide human exposure in various agricultural, hospital and veterinary disinfectant products. Despite many uses, an understanding of a cellular response to OPP and it's mechanism of action, targeted genes, and the connectivity between targeted genes and the rest of cell metabolism remains obscure.

**Results:**

Herein, we performed a genome-wide transcriptome analysis of the cellular responses of *S. aureus *when exposed to 0.82 mM of OPP for 20 and 60 min. Our data indicated that OPP downregulated the biosynthesis of many amino acids, which are required for protein synthesis. In particular, the genes encoding the enzymes of the diaminopimelate (DAP) pathway which results in lysine biosynthesis were significantly downregualted. Intriguingly, we revealed that the transcription of genes encoding ribosomal proteins was upregulated by OPP and at the same time, the genes encoding iron acquisition and transport were downregulated. The genes encoding virulence factors were upregulated and genes encoding phospholipids were downregulated upon 20 min exposure to OPP.

**Conclusion:**

By using microarray analysis that enables us to simultaneously and globally examine the complete transcriptome during cellular responses, we have revealed novel information regarding the mode of action of OPP on *Staphylococcus*: OPP inhibits anabolism of many amino acids and highly downregulates the genes that encode the enzymes involved in the DAP pathway. Lysine and DAP are essential for building up the peptidoglycan cell wall. It was concluded that the mode of action of OPP is similar to the mechanism of action of some antibiotics. The discovery of this phenomenon provides useful information that will benefit further antimicrobial research on *S. aureus*.

## Background

The U.S. Environmental Protection Agency (EPA) has endeavored to determine the efficacy and the mode of action of antimicrobials. At EPA, 5,000 antimicrobial products are registered, and hospital-level disinfectants are being tested against pathogens such as *S. aureus*, which is responsible for many infectious diseases, ranging from benign skin infections to life-threatening endocarditis and toxic shock syndrome [1]. One of the reasons EPA has exerted such efforts is that hospital-acquired infections are a serious threat to public health. Therefore, it is important to use appropriate antimicrobial agents with clear understanding of the subsequent effects to prevent infection outbreaks in health care environments [2].

The phenolic compound, ortho-phenylphenol (OPP), is an antimicrobial agent and an active ingredient of EPA-registered disinfectant with wide human exposure in various agricultural, hospital and veterinary disinfectant products. OPP is employed in a variety of applications, including hard surface disinfection, wood preservation, treatment of citrus fruit, vegetables before packaging to prevent microbial decay and textile production due to its bactericidal and fungicidal activity [3-5].

There have been several reports related to the exposure of OPP on humans. It has been reported that OPP increased the incidence of urinary bladder tumors in F344/DuCrj rats when administered in the diet [6]. The results of this study stimulated the initiation of additional testing of OPP for both tumor induction and possible reactivity with DNA. OPP has been found to have estrogenic or anti-androgenic activity, and binds to the androgen or estrogen receptors [7]. In spite of these effects OPP is still used in applications that simultaneously contact both humans and bacteria. It is therefore important to understand the differential effects on each so that its efficacy can be understood and even optimized.

Moreover, a lack of understanding of a cellular response to OPP hinders further development of more innovative methods for combating pathogens. Certainly, better elucidation of the molecular events responsible for establishing and maintaining pathogenicity will help to map affected cell functions and serve to delineate the mechanisms involved in the disinfectant activity.

Microarrays have been effectively employed to simultaneously and globally examine the complete transcriptional response at the genomic level in *Pseudomonas aeruginosa *and *S. aureus *upon exposure to antimicrobials [8-15].

In this study, to our knowledge, for the first time, we show that the global transcription response of *S. aureus *to OPP includes downregulation of genes involved in lysine metabolism, as well as genes involved in amino acid metabolism, by utilizing Affymetrix *S. aureus *GeneChip arrays. Our findings indicate that: (i) many cellular protective processes were upregulated, (ii) the transcription of genes involved in primary metabolic pathways was downregulated, and (iii) the transcription of genes encoding lysine and histidine biosynthesis was downregulated. Next we performed real-time PCR analysis on selected genes to validate the array results. Based on this result, it was concluded that this study may help to elucidate the mechanism of action by which OPP stops cell wall construction and thereby inhibiting *S. aureus *growth, and may facilitate the design of more effective antimicrobials.

## Results and discussion

### Growth inhibition by OPP

**Figure 1:**
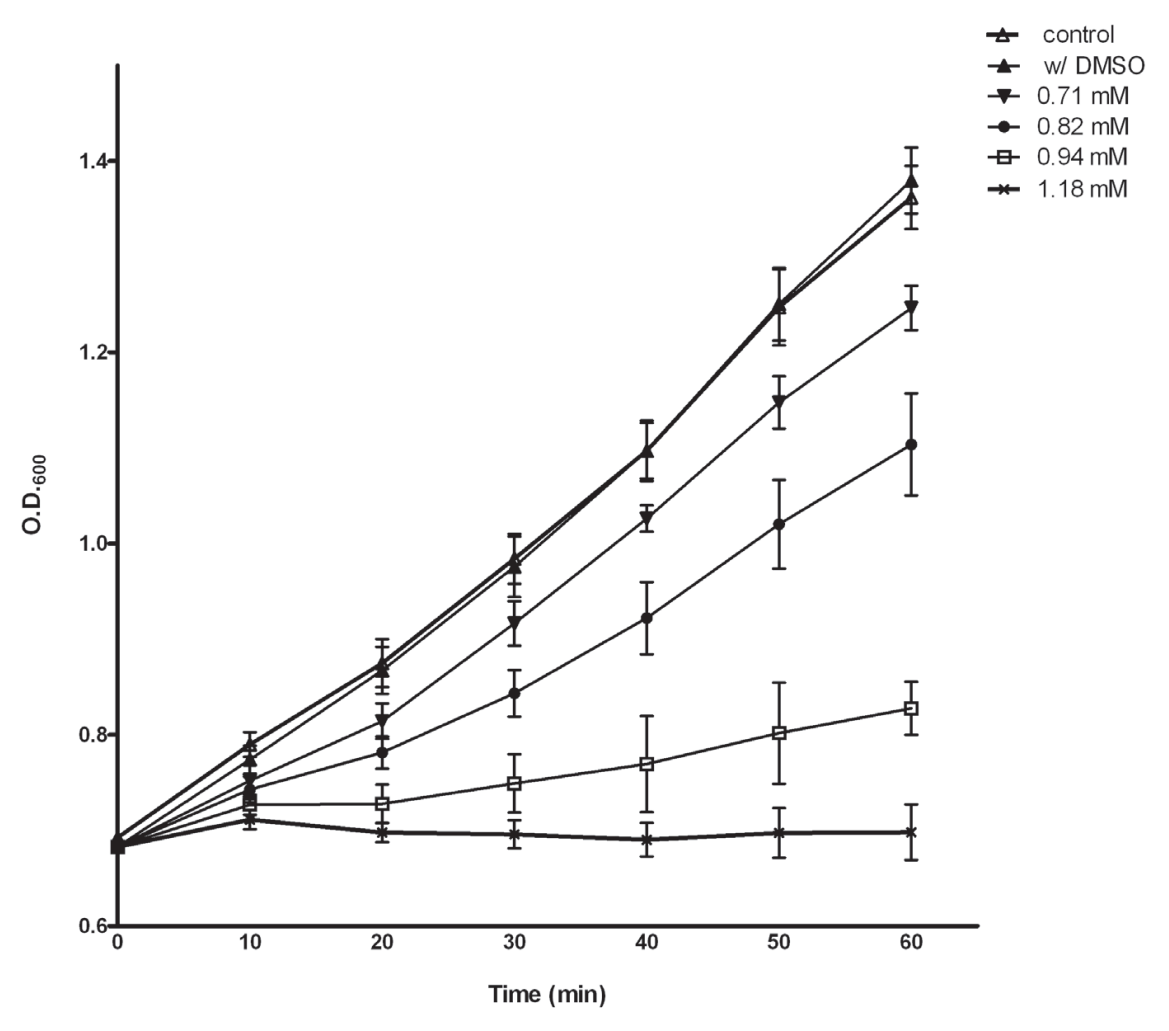
***S. aureus *growth (optical density at 600 nm) after treatment with OPP: control (open *triangles*), with DMSO (*closed triangles*), 0.71 mM (*inverted closed triangles*), 0.82 mM (*closed circles*), 0.94 mM (*open squares*), and 1.18 mM OPP (*X*)**. Growth inhibition was performed during the exponential phase of the cells without DMSO (control) and control with DMSO, 0.71 mM, 0.82 mM, 0.94 mM, and 1.18 mM OPP dissolved in DMSO. The results are the mean of triplicate experiments; the error bars represent standard deviation.

To determine the sublethal inhibitory effect of OPP on *S. aureus*, we first exposed the exponentially growing cells to different concentrations of OPP dissolved in DMSO (0 up to 1.18 mM). In figure 1, we demonstrate that 0.82 mM concentration of OPP caused a growth inhibition for about 20 min. Note that minimum inhibitory concentration (MIC50) of OPP on *S. aureus *was reportedly 500 mg/l (3 mM) [16]. In this study, to better understand how *S. aureus *initially responds to OPP, we chose the rate of cell growth inhibition with 0.82 mM OPP after 20 and 60 min exposure times compared to control (without OPP).

### Transcriptional profiles in response to OPP

To investigate early transcriptional changes in response to OPP exposure, we isolated total RNA after 20 min and 60 minutes exposure to 0.82 mM OPP and conducted five independent microarray experiments in the absence (control) and the presence (experimental) of 0.82 mM OPP (see figure 1). To further identify genes with statistically marked changes in expression levels, we applied the following criteria to each of the 20 min, 60 min, and control-experimental microarray data sets: (i) a *p-*value for a *t*-test should be equal to or less than 0.05, (ii) an absolute fold change in transcript level should be equal to or greater than 2, and (iii) a gene should have a presence or marginal call [17] from 50% or more replicates on both the experimental and control replicate sets. Of the 7,775 genes represented on the *S. aureus *GeneChip, 2,348 genes showed statistical significance based on a 1-way ANOVA. We found that mRNA levels 669 genes of *S. aureus *were significantly altered in response to OPP by two fold or more upregulation or downregulation. The raw data of 7,775 genes control (0 min) and experimental genes after (20 and 60 min exposure to 0.82 mM of OPP) has been deposited in NCBI's Gene Expression Omnibus [18] and is accessible through GEO Series accession number GSE10605 (Additional file 1).

### Analysis of gene expression changes in 20 min and 60 min

To examine how genes with transcript level changes are distributed with regard to their functions, we further classified these 669 genes that were either upregulated or downregulated by a fold change of two or more according to the Gene Classification based on COG functional categories in the genome of National Center for Biotechnology information (NCBI) [19] (see also Additional file 2).

**Figure 2:**
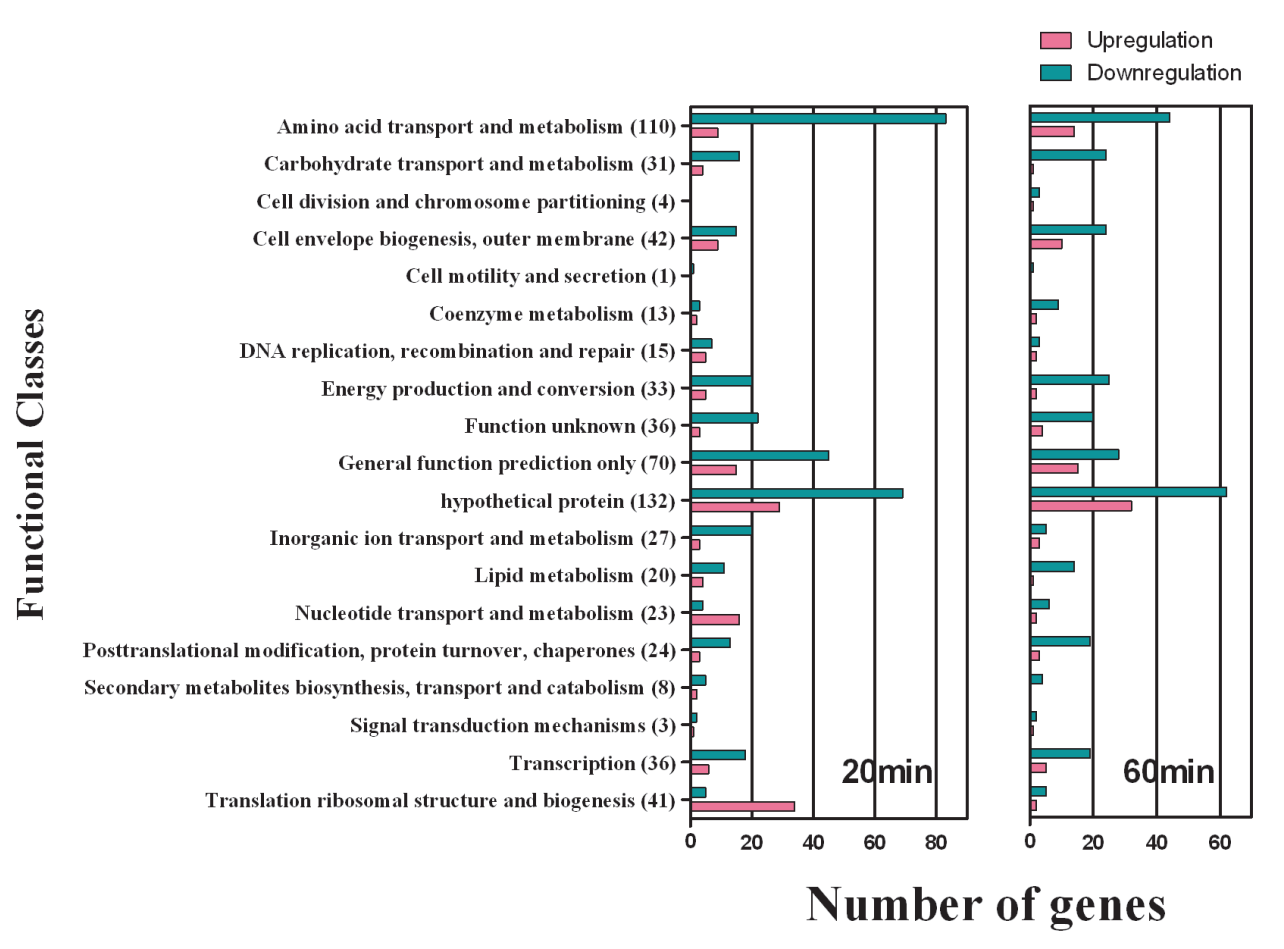
**Functional classification of genes with statistically significant upregulated (*red*) and downregulated (*green*) when exposed to 0.82 mM OPP at 20 min and 60 min (a total of 669 genes)**. The number in parenthesis represents the total number of genes affected within the genome in each functional class.

In Figure 2, the differences between the numbers of up and downregulated genes in each functional class after 20 and 60 minutes exposure to 0.82 mM of OPP are illustrated. Note that Figure 2 represents a total of 669 genes including the group of "function unknown (36), hypothetical protein (132) and general function predicted only (70)". Some interesting findings are as follows: (i) the genes of amino acid transport and metabolism were highly downregulated at both 20 and 60 min; (ii) the genes of inorganic ion transport and metabolism were downregulated at 20 min and decrease in the number of genes downregulated at 60 min was also observed; (iii) the genes in the class of "translation, ribosomal structure and biogenesis" were significantly upregulated at 20 min; (iv) the number of genes involved in nucleotide transport and metabolism were increased after 20 min compared to after 60 min. In general, figure 2 illustrates that the functional classes contained more downregulated and fewer upregulated genes at 20 min. This result suggests that the functional class profiles were notably different between 20 and 60 min, and this difference might explain why *S. aureus *underwent the initial growth inhibition followed by partial growth recovery upon exposure to OPP.

### Functional classifications analysis

**Figure 3:**
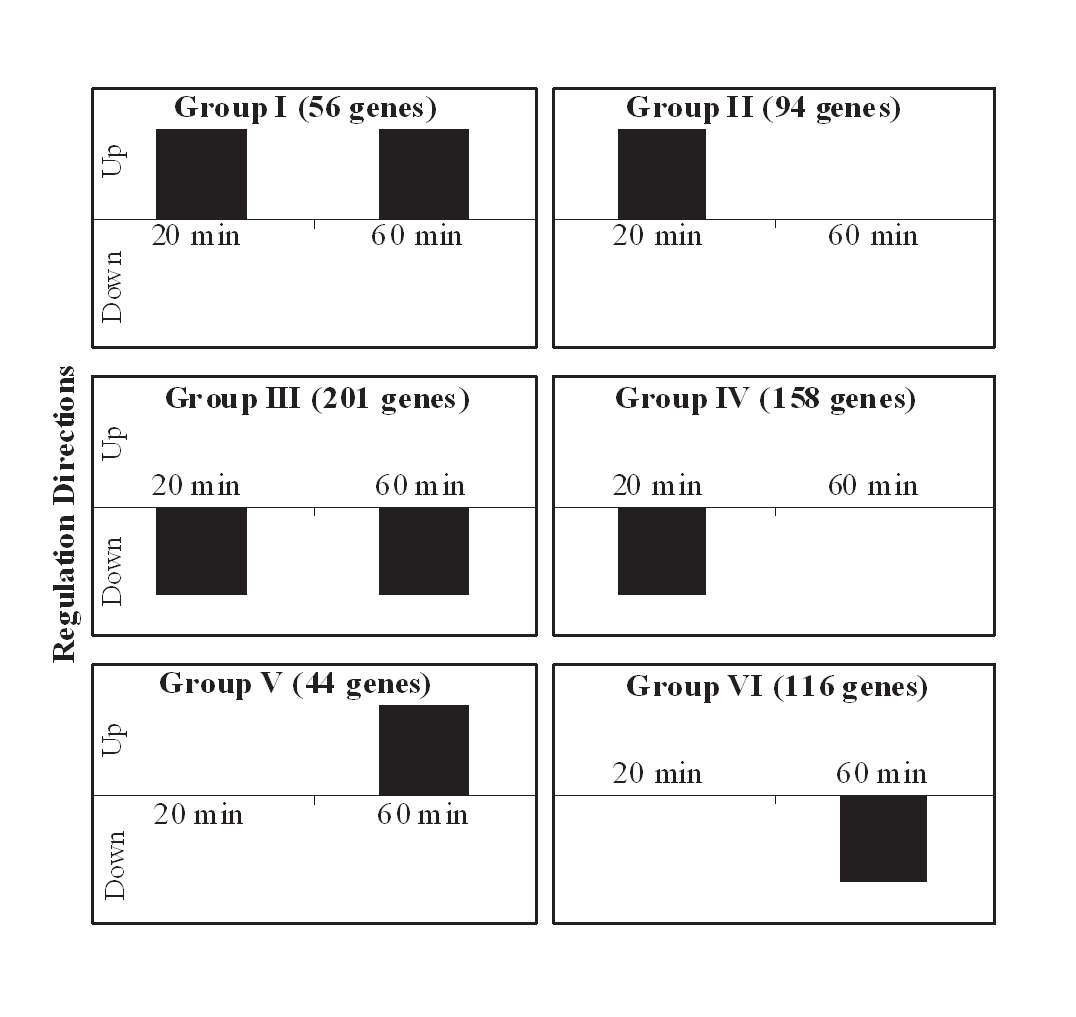
**Groups of differentially regulated 431 genes with known functional class, which are categorized by their transcription directions upon 20 and 60 min exposures**. Group I contained 23 genes upregulated upon both exposure times, while group II had 80 genes upregulated at 20 min and no significant changes upon 60 min exposure. Further, group III possessed 26 genes that were upregulated in response to 60 min exposure. Group IV contained 128 genes downregulated upon both exposure times, whereas 95 genes of group V exhibited downregulation after 20 min exposure. Finally, group VI had 79 genes that were downregulated upon 60 min exposure.

**Figure 4:**
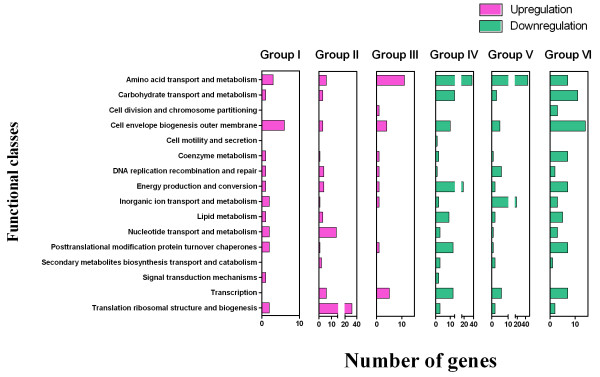
**Functional classification of genes with statistically significant upregulated (*red*) and downregulated (*green*) upon 20 min and 60 min exposures (a total of 431 genes)**. Note that the functional classes of "hypothetical genes", "general function prediction only" and "function unknown" are not included in this figure.

To further identify genes with similar transcription patterns during the time course, we removed 238 genes (including the group of functional unknown (36), hypothetical protein (132) and general function predicted only (70)). We categorized 431 genes with known functions into 6 groups on the basis of their transcription directions (figure 3). Briefly, group I contained 23 genes upregulated upon both exposure times, while group II had 80 genes with increased expression levels at 20 min and no significant changes upon 60 min exposure. Further, group III possessed 26 genes that were upregulated at 60 min exposure. Group IV contained 128 genes downregulated upon both exposure times, whereas 95 genes of group V exhibited downregulation after 20 min. Finally, group VI had 79 genes that were downregulated upon 60 min exposure. Figure 4 displays the number of genes (431) within groups I through VI in each functional class. As indicated above, additional file 2 contains all 669 genes including the genes classified under the functional group designated as "unknown, hypothetical, and general function prediction only".

Since most of the genes discussed in this report are in additional file 2, for further analysis of the data and for the readers convenience, we decided to make table 1 with 138 *S. aureus *genes that were most strongly upregulated or downregulated in response to OPP after 20 and 60 minutes exposure. These genes were also classified under seven groups based on their transcription directions

**Table 1:** List of 138 *S. aureus genes *that were most strongly affected by OPP and are discussed in this report categorized by their related function. The microarray results are the mean of five replicates of each gene.

		**20 min**		**60 min**				
					
**Affymetrix Probe ID**	**ORF no.**	**p-value**	**Fold change**	**p-value**	**Fold change**	**Description**	***Gene symbol***	**Functional class**
**Group I: Upregulation (20 min) – Upregulation (60 min) 18 genes**								

sa_c6812s5946_a_at	SA0265	8.55E-06	2.1	8.55E-06	3.1	peptidoglycan hydrolase (surface antigen)	*lyt*M	Cell envelope biogenesis, outer membrane
sa_c7382s10191_a_at	SA0423*	5.16E-07	8.1	5.16E-07	19.4	hypothetical protein, similar to autolysin (N-acetylmuramoyl-L-alanine amidase)		General function prediction only
sa_c7698s6703_a_at	SA0519	0.00293	2.8	0.00293	2.5	Ser-Asp rich fibrinogen-binding, bone sialoprotein-binding protein	*sdr*C	Cell envelope biogenesis, outer membrane
sa_c8045s7032_at	SA0620	1.27E-06	7.6	1.27E-06	5.6	hypothetical protein, similar to secretory antigen precursor SsaA		General function prediction only
sa_c592s9345_a_at	SA0905	4.83E-08	5.7	4.83E-08	7.0	N-acetylglucosaminidase (major autolysin)	*atl*	Cell envelope biogenesis, outer membrane
sa_c1007s793_a_at	SA1003	7.81E-06	3.7	7.81E-06	4.2	fibrinogen-binding protein precursor		hypothetical protein
sa_c4394s3743_a_at	SA1898	7.99E-05	6.1	7.99E-05	5.8	hypothetical protein, simialr to SceD precursor		hypothetical protein
sa_c4612s9984cs_s_at	SA1972	0.00267	2.2	0.00267	2.7	multidrug resistance protein (efflux transporter)		hypothetical protein
sa_c5066s4362_a_at	SA2093*	0.00025	6.8	0.00025	9.0	hypothetical protein, similar to secretory antigen precursor SsaA	*ssa*A	General function prediction only
sa_c5082s4380_a_at	SA2097*	3.71E-05	6.9	3.71E-05	11.5	hypothetical protein, similar to secretory antigen precursor SsaA		General function prediction only
sa_c342s182_a_at	SA2142	9.36E-07	3.6	9.36E-07	3.6	multidrug resistance protein B (drug efflux transporter)		hypothetical protein
sa_c5274s4572_a_at	SA2143	1.24E-07	3.9	1.24E-07	4.1	multidrug resistance efflux pump		hypothetical protein
sa_c5652s4904_a_at	SA2206	0.00082	2.5	0.00082	2.8	Immunoglobulin G binding protein A precursor	*sbi*	Cell envelope biogenesis, outer membrane
sa_c6151s5333_a_at	SA2332	0.00019	7.8	0.00019	8.6	hypothetical protein, similar to secretory antigen precursor SsaA		General function prediction only
sa_c6250s5428_a_at	SA2353*	1.91E-05	10.2	1.91E-05	13.8	secretory antigen precursor SsaA homolog		General function prediction only
sa_c9402s8223_a_at	SA2355*	3.23E-06	8.5	3.23E-06	9.4	transcriptional regulator, MARR family		hypothetical protein
sa_c6259s5439_a_at	SA2356	3.43E-06	6.5	3.43E-06	6.9	immunodominant antigen A	*isa*A	Cell envelope biogenesis, outer membrane
sa_c6506s5675_a_at	SA2423	0.00148	2.9	0.00148	3.5	fibrinogen-binding protein A, clumping factor	*clf*B	Posttranslational modification, protein turnover, chaperones

**Group II: Upregulation (20 min) – No change (60 min) 28 genes**								

sa_c7511s6531_a_at	SA0459	0.000138	2.3			ribosomal protein L25 (general stress protein Ctc)	*rpl*Y	Translation, ribosomal structure and biogenesis
sa_c7621s6634_a_at	SA0497	7.95E-05	2.2			50S ribosomal protein L10; ribosomal protein L10 (BL5)		Translation, ribosomal structure and biogenesis
sa_c7625s6638_at	SA0498	5.07E-05	3.5			50S ribosomal protein L7:L12; ribosomal protein L7:L12		Translation, ribosomal structure and biogenesis
sa_c1147s928_a_at	SA1041	0.00142	2.8			uracil phosphoribosyltransferase; Pyrimidine operon regulatory protein pyrR		Nucleotide transport and metabolism
sa_c1151s932_a_at	SA1042	0.000143	6.7			uracil permease (uracil transporter)	*pyr*P	Nucleotide transport and metabolism
sa_c9991s8687_a_at	SA1043	7.66E-05	6.5			aspartate carbamoyltransferase catalytic chain (Aspartate transcarbamylase) (ATCase)	*pyr*B	Nucleotide transport and metabolism
sa_c1155s937_a_at	SA1044	0.000101	6.0			dihydroorotase, dihydroorotase-like	*pry*C	Nucleotide transport and metabolism
sa_c1159s942_a_at	SA1045	0.000161	5.1			carbamoyl-phosphate synthase, arginine-specific, small chain	*pyr*AA	Amino acid transport and metabolism, Nucleotide transport and metabolism
sa_c1165s946_a_at	SA1046	0.00018	3.3			carbamoyl-phosphate synthase, arginine-specific, large chain	*car*B	Amino acid transport and metabolism, Nucleotide transport and metabolism
sa_c1167s950_a_at	SA1047	0.000788	3.3			orotidine 5-phosphate decarboxylase	*pyr*F	Nucleotide transport and metabolism
sa_c9989s8682_a_at	SA1048	0.00107	3.0			orotate phosphoribosyltransferase	*pyr*E	Nucleotide transport and metabolism
sa_c1302s1077_a_at	SA1084	0.000221	2.7			50S ribosomal protein L19; ribosomal protein L19	*rpl*S	Translation, ribosomal structure and biogenesis
sa_c4792s4098_at	SA2022	0.000417	2.1			50S ribosomal protein L17; ribosomal protein L17	*rpl*Q	Translation, ribosomal structure and biogenesis
sa_c4824s4130_a_at	SA2029	2.76E-05	2.1			50S ribosomal protein L15; ribosomal protein L15	*rpl*O	Translation, ribosomal structure and biogenesis
sa_c4836s4142_at	SA2032	0.00024	2.1			50S ribosomal protein L18; ribosomal protein L18	*rpl*R	Translation, ribosomal structure and biogenesis
sa_c9951s8647_at	SA2033	0.000342	2.6			50S ribosomal protein L6; ribosomal protein L6 (BL8)	*rpl*F	Translation, ribosomal structure and biogenesis
sa_c4848s4156_at	SA2035	0.0021	2.1			50S ribosomal protein L5; ribosomal protein L5 (BL6)	*rps*N	Translation, ribosomal structure and biogenesis
sa_c4852s4158_at	SA2036	0.00267	2.1			50S ribosomal protein L24; ribosomal protein L24 (BL23)	*rpl*X	Translation, ribosomal structure and biogenesis
sa_c10191s8871_a_at	SA2038	0.0026	2.8			30S ribosomal protein S17; ribosomal protein S17 (BS16)	*rps*Q	Translation, ribosomal structure and biogenesis
sa_c4860s4166_at	SA2039	0.00204	2.5			50S ribosomal protein L29; ribosomal protein L29	*rpm*C	Translation, ribosomal structure and biogenesis
sa_c4864s4170_at	SA2040	0.000749	2.7			50S ribosomal protein L16; ribosomal protein L16	*rpl*P	Translation, ribosomal structure and biogenesis
sa_c4868s4175_a_at	SA2041	0.0024	2.8			30S ribosomal protein S3; ribosomal protein S3 (BS3)	*rps*C	Translation, ribosomal structure and biogenesis
sa_c4876s4184_at	SA2043	0.00405	2.8			30S ribosomal protein S19; ribosomal protein S19 (BS19)	*rps*S	Translation, ribosomal structure and biogenesis
sa_c9959s8654_a_at	SA2044	0.000888	2.5			50S ribosomal protein L2; ribosomal protein L2 (BL2)	*rpl*B	Translation, ribosomal structure and biogenesis
sa_c10192s8875_a_at	SA2045	0.00434	2.7			50S ribosomal protein L23; ribosomal protein L23	*rpl*W	Translation, ribosomal structure and biogenesis
sa_c4880s4187_at	SA2046	0.00283	2.8			50S ribosomal protein L4; ribosomal protein L4	*rpl*D	Translation, ribosomal structure and biogenesis
sa_c4888s4195_a_at	SA2047	0.00069	2.3			50S ribosomal protein L3; ribosomal protein L3 (BL3)	*rpl*C	Translation, ribosomal structure and biogenesis
sa_c9963s8658_a_at	SA2048	0.00245	2.4			30S ribosomal protein S10; ribosomal protein S10 (BS13)	*rps*J	Translation, ribosomal structure and biogenesis

**Group III: No change (20 min) – Upregulation (60 min) 8 genes**								

sa_c10571s9056_a_at	SA0845			0.000581	2.5	putative oligopeptide ABC transporter integral membrane protein (fragment)	*opp*B	Amino acid transport and metabolism, Inorganic ion transport and metabolism
sa_c324s166_a_at	SA0846			0.00019	2.4	probable peptide ABC transporter permease ABC transporter protein	*opp*C	Amino acid transport and metabolism, Inorganic ion transport and metabolism
sa_c328s170_a_at	SA0847			0.00293	2.2	probable peptide ABC transporter ATP-binding ABC transporter protein	*opp*D	Amino acid transport and metabolism, Inorganic ion transport and metabolism
sa_c332s172_a_at	SA0848			9.13E-05	2.2	probable peptide ABC transporter ATP-binding ABC transporter protein	*opp*F	Amino acid transport and metabolism
sa_c5349s4625_a_at	SA0950			0.0022	2.2	ABC transporter ATP-binding protein – spermidine:putrescine transport	*pot*A	Amino acid transport and metabolism
sa_c795s596_a_at	SA0952			0.00536	2.2	ABC transporter membrane-spanning permease – spermidine:putrescine transport	*pot*C	Amino acid transport and metabolism
sa_c803s604_a_at	SA0953			0.00987	2.2	spermidine:putrescine ABC transporter, spermidine: putrescine-binding periplasmic protein (potD) homolog	*pot*D	Amino acid transport and metabolism
sa_c8848s7783_a_at	SA1601			0.000923	2.3	CRCB, CrcB-like protein		Cell division and chromosome partitioning

**Group IV: Downregulation (20 min) – Downregulation (60 min) 27 genes**								

sa_c5061s4360_a_at	SA0229	4.85E-07	-5.8	4.85E-07	-4.8	dipeptide ABC transporter, periplasmic dipeptide-binding protein (dppA)		Amino acid transport and metabolism
sa_c736s544_a_at	SA0937	0.00365	-2.9	0.00365	-3.5	cytochrome D ubiquinol oxidase subunit I		Energy production and conversion
sa_c740s548_a_at	SA0938	0.00375	-2.9	0.00375	-3.4	cytochrome D ubiquinol oxidase subunit II homolog		Energy production and conversion
sa_c1659s1395_a_at	SA1164*	0.000431	-8.9	0.000431	-3.4	homoserine dehydrogenase (HDH)	*dho*M	Amino acid transport and metabolism
sa_c1665s1401_a_at	SA1165	0.00011	-8.3	0.00011	-3.2	threonine synthase (EC 4.2.3.1) homolog thrC	*thr*C	Amino acid transport and metabolism
sa_c1669s1406_a_at	SA1166	5.06E-05	-11.5	5.06E-05	-3.8	homoserine kinase (thrB)	*thr*B	Amino acid transport and metabolism
sa_c1872s1598_a_at	SA1213	0.00127	-2.8	0.00127	-2.4	probable peptide ABC transporter permease ABC transporter protein	*opp-*2C	Amino acid transport and metabolism, Inorganic ion transport and metabolism
sa_c1876s1602_a_at	SA1214	8.58E-06	-3.7	8.58E-06	-3.0	putative oligopeptide ABC transporter integral membrane protein (fragment)	*opp*-2B	Amino acid transport and metabolism, Inorganic ion transport and metabolism
sa_c1912s1635_a_at	SA1225*	8.90E-06	-54.6	8.90E-06	-7.7	aspartokinase II in bifunctional enxyme: aspartokinase II; homoserine dehydrogenase II	*lys*C	Amino acid transport and metabolism
sa_c1918s1640_a_at	SA1226*	1.78E-05	-21.5	1.78E-05	-4.3	aspartate-semialdehyde dehydrogenase (ASA dehydrogenase) (ASA DH)	*asd*	Amino acid transport and metabolism
sa_c1922s1644_a_at	SA1227*	2.75E-07	-27.3	2.75E-07	-5.2	dihydrodipicolinate synthase (DHDPS)	*dap*A	Amino acid transport and metabolism, Cell envelope biogenesis, outer membrane
sa_c1924s1648_a_at	SA1228*	1.17E-07	-31.4	1.17E-07	-5.1	dihydrodipicolinate reductase (DHPR)	*dap*B	Amino acid transport and metabolism
sa_c1928s1652_a_at	SA1229*	2.55E-07	-23.5	2.55E-07	-4.4	2,3,4,5-tetrahydropyridine-2,6-dicarboxylate N-succinyltransferase	*dap*D	Amino acid transport and metabolism
sa_c1936s1659_a_at	SA1231	8.94E-08	-18.5	8.94E-08	-3.0	Ala_racemase, Alanine racemase		Cell envelope biogenesis, outer membrane
sa_c3202s2750_a_at	SA1544	2.74E-05	-15.8	2.74E-05	-3.8	serine-pyruvate aminotransferase; alanine-glyoxylate aminotransferase (Serine-pyruvate aminotransferase)		Amino acid transport and metabolism
sa_c3603s3083_a_at	SA1659	7.54E-06	-2.4	7.54E-06	-3.5	parvulin-like PPIase precursor (Peptidyl-prolyl cis-trans isomerase plp) (Rotamase plp)	*prs*A	Posttranslational modification, protein turnover, chaperones
sa_c10591s11046_s_at	SA1814	1.99E-05	-3.3	1.99E-05	-2.9	succinyl-diaminopimelate desuccinylase (dapE)		Amino acid transport and metabolism
sa_c5023s4322_at	SA2082	6.38E-07	-3.5	6.38E-07	-5.5	urease gamma chain (urea amidohydrolase)	*ure*A	Amino acid transport and metabolism
sa_c5029s4326_a_at	SA2083	2.01E-06	-4.3	2.01E-06	-7.6	URE2_staxy urease beta subunit	*ure*B	Amino acid transport and metabolism
sa_c5031s4330_a_at	SA2084	1.84E-07	-3.0	1.84E-07	-4.4	urease alpha chain (urea amidohydrolase)	*ure*C	Amino acid transport and metabolism
sa_c5035s4334_at	SA2085	1.71E-05	-2.9	1.71E-05	-4.2	urease accessory protein UreE	*ure*E	Posttranslational modification, protein turnover, chaperones
sa_c5039s4340_a_at	SA2086	1.40E-05	-2.3	1.40E-05	-3.8	urease accessory protein UreF	*ure*F	Posttranslational modification, protein turnover, chaperones
sa_c9293s8136_a_at	SA2088	0.000118	-2.2	0.000118	-2.8	urease accessory protein UreD	*ure*D	Posttranslational modification, protein turnover, chaperones
sa_c5303s4583_a_at	SA2149	1.28E-07	-35.8	1.28E-07	-64.9	probable peptide ABC transporter ATP-binding ABC transporter protein		hypothetical protein
sa_c5307s4587_at	SA2150	9.33E-07	-36.5	9.33E-07	-69.0	ABC-type transporter, permease component		hypothetical protein
sa_c5777s5020_a_at	SA2235	2.82E-05	-5.1	2.82E-05	-3.5	putative ABC transporter; osmoprotectant-binding protein,	*opu*CC	Cell envelope biogenesis, outer membrane
sa_c6435s5604_a_at	SA2409	0.000134	-4.6	0.000134	-2.2	anaerobic ribonucleoside-triphosphate reductase activating protein		Posttranslational modification, protein turnover, chaperones

**Group V: Downregulation (20 min) – No change (60 min) 35 genes**								

sa_c37s34_a_at	SA0010	2.44E-06	-4.7			branched-chain amino acid permease		Amino acid transport and metabolism
sa_c4055s3432_a_at	SA0201	0.00111	-2.3			RGD-containing lipoprotein	*rlp*	Amino acid transport and metabolism
sa_c7055s6165_a_at	SA0331	0.000236	-3.0			probable lipoprotein		Inorganic ion transport and metabolism
sa_c7100s6210_a_at	SA0344	2.80E-06	-15.2			methyltetrahydropteroyltriglutamate – homocysteine methyltransferase (vitamin-B12-independent methionine synthase isozyme)	*met*E	Amino acid transport and metabolism
sa_c5418s4689_a_at	SA0420	7.55E-05	-2.8			probable amino acid ABC transporter, ATP-binding protein (abc)		Inorganic ion transport and metabolism
sa_c7374s6406_a_at	SA0421	6.63E-07	-3.3			putative amino acid ABC transporter, permease protein, glutamine transport system		Inorganic ion transport and metabolism
sa_c5431s4700_a_at	SA0769	2.32E-06	-5.4			probable amino acid ABC transporter, ATP-binding protein		Inorganic ion transport and metabolism
sa_c8512s7471_a_at	SA0770	1.42E-05	-6.3			permease protein of ABC transporter system		Inorganic ion transport and metabolism
sa_c8518s7475_a_at	SA0771	5.12E-06	-7.5			probable D-methionine-binding lipoprotein metQ precursor (Outer membrane lipoprotein1)		Inorganic ion transport and metabolism
sa_c350s191_a_at	SA0849	8.12E-05	-2.1			oligopeptide ABC transporter, periplasmic oligopeptide-binding protein (oppA-2)		Amino acid transport and metabolism
sa_c352s195_a_at	SA0850	0.000757	-3.4			periplasmic oligopeptide-binding protein of oligopeptide ABC transporter		Amino acid transport and metabolism
sa_c1820s1547_a_at	SA1200	0.00698	-2.1			para-aminobenzoate synthetase glutamine amidotransferase component II		Amino acid transport and metabolism, Coenzyme metabolism
sa_c1828s1551_a_at	SA1201	0.000803	-2.8			pir|AE0653 anthranilate synthase component II,	*trp*D	Amino acid transport and metabolism
sa_c1832s1558_a_at	SA1202	0.00239	-4.5			anthranilate synthase; indole-glycerol phosphate synthase;	*trp*C	Amino acid transport and metabolism
sa_c1836s1562_at	SA1203	0.00279	-4.3			indole-3-glycerolphosphate synthetase	*trp*F	Amino acid transport and metabolism
sa_c1840s1566_a_at	SA1204	0.000663	-3.6			tryptophan synthase beta chain; tryptophan synthase (beta subunit)	*trp*B	Amino acid transport and metabolism
sa_c1844s1570_a_at	SA1205	5.52E-05	-2.6			tryptophan synthase alpha chain; tryptophan synthase	*trp*A	Amino acid transport and metabolism
sa_c1866s1587_a_at	SA1211	0.000158	-2.3			ATP-binding ABC transporter protein	*opp*-2F	Amino acid transport and metabolism
sa_c4209s3561_a_at	SA1858	0.000144	-6.2			dihydroxy-acid dehydratase (DAD)	*ilv*D	Amino acid transport and metabolism, Coenzyme metabolism
sa_c4213s3565_a_at	SA1859	0.000431	-8.0			acetolactate synthase isozyme III large subunit (AHAS-III)	*ilv*B	Amino acid transport and metabolism, Coenzyme metabolism
sa_c9931s8627_a_at	SA1861	0.000255	-10.3			ketol-acid reductoisomerase (Acetohydroxy-acid isomeroreductase)	*ilv*C	Amino acid transport and metabolism, Coenzyme metabolism
sa_c4223s3575_a_at	SA1862	4.06E-05	-9.8			2-isopropylmalate synthase	*leu*A	Amino acid transport and metabolism
sa_c4225s3576_a_at	SA1863	5.85E-05	-11.0			3-isopropylmalate dehydrogenase	*leu*B	Energy production and conversion, Amino acid transportand metabolism
sa_c4229s3580_a_at	SA1864	1.39E-05	-13.2			3-isopropylmalate dehydratase large subunit	*leu*C	Amino acid transport and metabolism
sa_c4239s3588_a_at	SA1865	6.09E-05	-13.1			3-isopropylmalate dehydratase small subunit	*leu*D	Amino acid transport and metabolism
sa_c4243s3594_a_at	SA1866	1.58E-05	-7.3			threonine dehydratase biosynthetic (Threonine deaminase) (TD)	*ilv*A	Amino acid transport and metabolism
sa_c9447s10370cs_s_at	SA2464*	0.00225	-10.5			histidine biosynthesis bifunctional protein	*his*I	Amino acid transport and metabolism
sa_c6696s10090cs_s_at	SA2465*	0.00199	-10.6			Imidazole glycerol phosphate synthase subunit hisF	*his*F	Amino acid transport and metabolism
sa_c6706s5846_a_at	SA2466*	0.00148	-14.5			phosphoribosylformimino-5-aminoimidazole carboxamide ribotide isomerase		Amino acid transport and metabolism
sa_c6708s5850_a_at	SA2467	0.00213	-14.6			Imidazole glycerol phosphate synthase subunit hisH	*his*H	Amino acid transport and metabolism
sa_c6714s5853_a_at	SA2468*	0.00639	-13.6			Imidazoleglycerol-phosphate dehydratase (IGPD)	*his*B	Amino acid transport and metabolism
sa_c6718s5857_a_at	SA2469	0.00106	-19.8			histidinol-phosphate aminotransferase		Amino acid transport and metabolism
sa_c6728s5871_a_at	SA2472	0.0059	-14.5			ATPphosphoribosyltransferase regulatory subunit	*his*Z	Amino acid transport and metabolism
sa_c6740s5882_a_at	SA2475	0.000217	-6.6			ABC transporter membrane-spanning permease – unknown substrate		Inorganic ion transport and metabolism
sa_c5397s4673_a_at	SA2476	0.000459	-7.6			putative ABC transporter; ATP-binding protein; possible cobalt transport system		Inorganic ion transport and metabolism

**Group VI: No change (20 min) – Downregulation (60 min) 19 genes**								

sa_c2346s1974_a_at	SA0144			4.02E-05	-3.1	capsular polysaccharide synthesis enzyme Cap5A	*cap*A	Cell envelope biogenesis, outer membrane
sa_c2385s1987_a_at	SA0145			4.55E-06	-2.9	capsular polysaccharide synthesis enzyme Cap5B	*cap*B	Cell division and chromosome partitioning
sa_c2399s1991_a_at	SA0146			0.00055	-2.6	capsular polysaccharide synthesis enzyme Cap8C	*cap*C	Cell envelope biogenesis, outer membrane, Carbohydrate transport and metabolism
sa_c2413s1997_a_at	SA0147			0.0001	-2.6	capsular polysaccharide synthesis enzyme Cap5D	*cap*D	Cell envelope biogenesis, outer membrane, Carbohydrate transport and metabolism
sa_c9546s8318_a_at	SA0148			9.77E-05	-2.8	putative UDP-glucose 4-epimerase (Galactowaldenase) (UDP-galactose 4-epimerase)	*cap*E	Cell envelope biogenesis, outer membrane, Carbohydrate transport and metabolism
sa_c2479s2056_a_at	SA0149			2.40E-06	-3.1	capsular polysaccharide synthesis enzyme Cap5F	*cap*F	Cell envelope biogenesis, outer membrane, Carbohydrate transport and metabolism
sa_c2516s2092_a_at	SA0150			5.92E-05	-2.4	UDP-N-acetylglucosamine 2-epimerase (UDP-GlcNAc-2-epimerase)	*cap*G	Cell envelope biogenesis, outer membrane
sa_c10086s8810_a_at	SA0151			0.00025	-2.8	chloramphenicol acetyltransferase (Xenobiotic acetyltransferase) (XAT)	*cap*H	General function prediction only
sa_c10087s8814_a_at	SA0152			0.00017	-2.7	capsular polysaccharide synthesis enzyme Cap5I	*cap*I	Cell envelope biogenesis, outer membrane
sa_c10089s8822_a_at	SA0154			0.00103	-2.1	capsular polysaccharide synthesis enzyme Cap5K	*cap*K	Cell envelope biogenesis, outer membrane
sa_c898s698_a_at	SA0977			0.00044	-2.7	29-kDa cell surface protein	*isd*A	Cell envelope biogenesis, outer membrane
sa_c906s704_a_at	SA0978			0.00538	-2.2	hypothetical protein SirD	*isd*C	Cell envelope biogenesis, outer membrane
sa_c3380s9339_a_at	SA1586			1.41E-05	-2.4	6,7-dimethyl-8-ribityllumazine synthase (DMRL synthase)	*rib*H	Coenzyme metabolism
sa_c3387s2918_a_at	SA1587			6.15E-06	-2.8	probable riboflavin biosynthesis bifunctional protein	*rib*A	Coenzyme metabolism
sa_c3391s2919_a_at	SA1588			3.10E-06	-2.7	riboflavin synthase alpha chain	*rib*B	Coenzyme metabolism
sa_c3395s2925_a_at	SA1589			5.10E-07	-3.0	riboflavin specific deaminase	*rib*D	Coenzyme metabolism
sa_c4369s3721_at	SA1894			0.000661	-2.3	thiamine-phosphate pyrophosphorylase	*thi*E	Coenzyme metabolism
sa_c4373s3725_a_at	SA1895			3.05E-05	-2.5	hydroxyethylthiazole kinase	*thi*M	Coenzyme metabolism
sa_c4379s3726_a_at	SA1896			0.000137	-2.8	bifunctional enzyme: hydroxy-phosphomethylpyrimidine kinase	*thi*D	Coenzyme metabolism

**Group VII: Downregulation (20 min) – Upregulation (60 min) 3 genes**								

sa_c9442s8255_a_at	SA2459*	0.0016	-2.5	0.0016	2.5	intercellular adhesion protein IcaA	*ica*A	Cell envelope biogenesis, outer membrane
sa_c6677s5830_a_at	SA2460*	0.0136	-2.0	0.0136	4.7	IcaD	*ica*D	Cell envelope biogenesis, outer membrane
sa_c6681s9106_a_at	SA2461*	3.83E-06	-2.1	3.83E-06	3.4	intercellular adhesion protein IcaB	*ica*B	Cell envelope biogenesis, outer membrane

* Genes were validated by real-time PCR.

### Group I: genes upregulated upon 20 and 60 min exposures

Group I of table 1 contains 18 genes associated with virulence in *S. aureus*. Interestingly, five of these genes encode the secretory antigen precursor, SsaA. The *ssa*A gene potentially regulated by the YycG/YycF system encodes proteins involved in cell wall metabolism, membrane-bound transport systems, and pathogenicity, including two major antigenic proteins, SsaA and IsaA. YycF has also been shown to bind specifically to the promoter regions of two genes, encoding the IsaA antigen and the LytM peptidoglycan hydrolase. This is in agreement with the proposed role of this system in controlling virulence and cell wall metabolism [20].

In this study, OPP also upregulated the *clf*B (clumping factor B) gene expression upon both 20 and 60 min exposure. In our previous results, triclosan upregulated the expression of SA2423 encoding ClfB, which binds fibrinogen [12]. The results of this study show that the production of virulence factors in *S. aureus *may be a secondary effect of OPP and this may provide new insight into the protective response of *S. aureus *to OPP.

### Group II: genes upregulated upon 20 min exposure

Group II of table 1 indicates that the class of "translation, ribosomal structure and biogenesis" which is responsible for the synthesis of ribosomal proteins was upregulated after 20 minutes. In group II of table 1, for instance four genes encode 30S ribosomal proteins and 15 genes code for 50S ribosomal proteins. In addition, SA0459 (*rplY*) which encoded general stress protein was upregulated at 20 min. Ribosomal proteins are required for protein translation. Such early response of these ribosomal protein genes may reflect a stress response during exposure. The upregulation of ribosomal protein genes might enhance the translation process or help proper ribosome functioning under stress conditions as exposure to OPP. The suggestion that the expression of ribosomal proteins is activated upon exposure to OPP is surprising as this presumably reflects increased growth rate or virulence.

In group II (table 1), we also observed genes related to primary metabolism that mainly belonged to the functional classes of "purines, pyrimidines, nucleosides, and nucleotides". The gene cluster: SA1041–SA1048 (*pyr*RPBCAAABFE) which is homologous to the pyrimidine biosynthetic (*pyr*) operon of *Bacillus subtilis *[21] was upregulated at 20 min.

### Group III: genes upregulated upon 60 min exposure

In group III of table 1, there were some genes from amino acid transport and metabolism, an ATP-binding cassette (ABC) transporters and transcription. The oligopeptide transport system (Opp) of *S. aureus *is an ABC transporter that transports amino acids, cations- and iron-carrying compounds and peptides with a broad specificity [22]. The peptides are mainly used as nutrients by the multiple amino acid auxotrophic *S. aureus*. The Opp system consists of four different proteins: OppB and OppC, OppD and OppF. Interestingly, 4 of the 26 genes, including SA0845–SA0848 that code for proteins associated with amino acid transport were upregulated (table 1). Therefore, the suggestion that the expression of these proteins is activated upon exposure to OPP is surprising as this presumably reflects increased growth rate or recovery. We found that a putative operon containing four open reading frames (ORFs) (*pot*ABCD) was upregulated (table 1). The *pot*ABCD operon encodes a periplasmic binding protein dependent ABC transport systems from Gram-positive bacteria [23]. The SA0950–SA0952–SA0953 shows homology to the genes encoding this *pot*ABCD transport system (*pot*A, *pot*C and *pot*D), which are involved in the transport of spermidine and putrescine. Further, we showed the upregulation of ABC transport systems-related genes, which accompanied the growth recovery.

Of further importance was that group III contained genes related to integral membrane protein, which belonged to the functional class of "cell division and chromosome partitioning". SA1601 (*crc*B) is a putative integral membrane protein possibly involved in chromosome condensation (table 1).

### Group IV: genes downregulated upon 20 and 60 min exposures

In group IV in table 1, we noted that genes belonging to the functional classes of "amino acid transport and metabolism", "carbohydrate transport and metabolism", "energy production and conversion", "posttranslational modification protein turnover chaperones", "transcription" classes and putative lipoproteins were downregulated upon both exposure times.

Intriguingly, we observed the high downregulation of SA2149 and SA2150 (*hrt *A and B), the heme-regulated transport system, which consist of a novel transport system which plays a critical role in staphylococcal heme metabolism (table 1). Among the genes in the class of "amino acid transport and metabolism", SA1225 (*lys*C)-SA1226 (*asd*)-SA1227 (*dap*A)-SA1228 (*dap*B)-SA1229 (*dap*D), and SA1814 (*dap*E) fall within a predicted operon and are all involved in diaminopimelate (DAP) biosynthesis (table 1). The disruption of biosynthetic pathways involved in building up bacterial cell wall components is a common mode of action of antibiotics [24]. Penicillins [25], methicillin [26], cephalosporins [27] and glycopeptide drugs such as vancomycin [28] are all drugs that inhibit major steps in the construction of the peptidoglycan layer of bacterial cell walls. Lysine or its biosynthetic precursor, DAP [29], are essential to most bacteria for the synthesis of the peptidoglycan layer of the cell wall [30-33]. Since mammals neither make nor use DAP and require L-lysine is an essential amino acid that is supplied through dietary intake, inhibitors of the DAP biosynthetic pathway will probably not result in mammalian toxicity. Decisively SA1225 (*lys*C)-SA1226 (*asd*)-SA1227 (*dap*A)-SA1228 (*dap*B) and SA1229 (*dap*D) show fold highest decreases as -54.6, -21.5, -27.3, -31.4, and -23.5 folds at 20 min and -7.7, -4.3, -5.2, -5.1, and -4.4 folds at 60 min in this experiment (see also table 2). Our findings suggest that the mode of action of OPP may be related to bacterial biosynthesis of amino acids. Other genes of amino acids, including methionine, threonine, histidine and lysine were also highly down regulated at -15, -7, -19 and -54 folds. Therefore, this outcome in conjunction with the extensive downregulation of the genes encoding DAP biosynthesis suggests that OPP may inhibit construction of the peptidoglycan in cell wall of *S. aureus*. These genes were less downregulated at 60 min than at 20 min. These results suggest that OPP inhibits the growth of *S. aureus *at 20 min and that growth recovery occurs at 60 minutes, indicating a possible mechanism of action of OPP in *S. aureus*. In a similar study carried out using *Pseudomonas aeruginosa *treated with 0.82 mM OPP, we did not observe extensive downregulation of the genes involved in amino acid biosynthesis and specifically lysine biosynthesis (data not shown). This suggests that the mechanisms of action of 0.82 mM OPP on *P. aeruginosa *and *S. aureus *may differ.

**Table 2:** Transcript level comparison of *S. aureus *genes between real-time PCR and microarray analyses.

	mRNA level change with microarray		mRNA level change with real-time PCR			
			
Gene	Fold change		Fold change		Sense primer sequence	Antisense primer sequence
			
	20 min	60 min	20 min	60 min		
SA0423	8.1	19.4	9.1(± 1.4)	18.6(± 1.2)	5'-CGG GTG AAT CAG TGT GGG CAA TTT-3'	5'-TAT GAT CCG CCA CCT GAG TTC GTT-3'
SA1164	-8.9	-3.4	-40.9(± 5.0)	-2.1(± 0.1)	5'-TAT GAT CCG CCA CCT GAG TTC GTT-3'	5'-GAG TGT AGC AGG TGG TAT TCC GAT-3'
SA1225	-54.6	-7.7	-265.0(± 18.9)	-58.0(± 1.4)	5'-GAG TGT AGC AGG TGG TAT TCC GAT-3'	5'-TCA TCA GTT GGA TCC GCT TCA GCA-3'
SA1226	-21.5	-4.3	-14.9(± 3.3)	-2.1(± 0.7)	5'-TCA TCA GTT GGA TCC GCT TCA GCA-3'	5'-ACT TTA GGC AGA GGC GGT TCT GAT-3'
SA1227	-27.3	-5.2	-58.0(± 1.5)	-2.8(± 0.1)	5'-ACT TTA GGC AGA GGC GGT TCT GAT-3'	5'-AGT CTT GGG TCA GTG GCA TAC ACA-3'
SA1228	-31.4	-5.1	-35.3(± 1.6)	-1.6(± 0.4)	5'-AGT CTT GGG TCA GTG GCA TAC ACA-3'	5'-TGG GTG CAA CAG GAT TAG TAG GCA-3'
SA1229	-23.5	-4.4	-7.4(± 2.0)	-1.2(± 0.2)	5'-TGG GTG CAA CAG GAT TAG TAG GCA-3'	5'-TTC AAC TTC TTG CCC TGC AGA ACG-3'
SA2093	6.8	9.0	15.7(± 1.2)	4.6(± 0.5)	5'-TTC AAC TTC TTG CCC TGC AGA ACG-3'	5'-TAT TTG AGG GTG TTG GCG TTG CAC-3'
SA2097	6.9	11.5	5.5(± 1.8)	24.5(± 1.2)	5'-TAT TTG AGG GTG TTG GCG TTG CAC-3'	5'-AGG GCT CTC AGC AGT AGT TCC ATT-3'
SA2353	10.2	13.8	8.0(± 1.6)	16.4(± 1.9)	5'-AGG GCT CTC AGC AGT AGT TCC ATT-3'	5'-ATT CGT GGA GGT ACG ATT GTC GGT-3'
SA2355	8.5	9.4	5.3(± 1.3)	20.1(± 1.7)	5'-ATT CGT GGA GGT ACG ATT GTC GGT-3'	5'-GCT GCT TGT ATA GCA CCA TTC GCA-3'
SA2459^c^	-2.5	2.5	-3.1(± 0.2.)	2.0(± 0.3)	5'-TTG TCG ACG TTG GCT ACT GGG ATA-3'	5'-TGG AAC CAA CAT CCA ACA CAT GGC-3'
SA2460^c^	-2.0	4.7	-1.8(± 0.3)	2.5(± 0.3)	5'-ATG GTC AAG CCC AGA CAG AGG GAA TA-3'	5'-CAC ACG ATA TAG CGA TAA GTG CTG TT-3'
SA2461^c^	-2.1	3.4	-2.6(± 0.5)	2.1(± 0.5)	5'-AGC AGT CAC TCC GAA CTC CAA TGA-3'	5'-TCA TGG AAT CCG TCC CAT CTC T-3'
SA2464^b^	-10.5	-	-5.5(± 1.4)	-	5'-GCT GCT TGT ATA GCA CCA TTC GCA-3'	5'-GAT CGT CGC AAT TCT GCC ATT CCA-3'
SA2465^b^	-10.6	-	-13.0(± 1.4)	-	5'-GAT CGT CGC AAT TCT GCC ATT CCA-3'	5'-TTG TTG CGC CCA TCA TAA CGA CAG-3'
SA2466^b^	-14.5	-	-5.7(± 1.3)	-	5'-TTG TTG CGC CCA TCA TAA CGA CAG-3'	5'-ACC GTA CTG GTG GTT TAG GTG CAA-3'
SA2468^b^	-13.6	-	-58.1(± 1.3)	-	5'-ACC GTA CTG GTG GTT TAG GTG CAA-3'	5'-TGA ACG GCC ATT TGA TGA TGG AGC-3'
SA0727^a^	1.00	1.00	1.00	1.00	5'-GAT GGT GGT TTC CGC GTA AAT GGT-3'	5'-GCG CCT GCT TCA ATA TGA GCT TGT-3'

The real time PCR results are the mean of three biological replicates with three technical replicates for each gene. The microarray results are the mean of five replicates of each gene.

^a^SA0727 was glyceraldehyde 3-phosphate dehydrogenase (GAPDH) and used as the house-keeping gene. ^b^SA2464, SA2465, SA2466, and SA2468 were downregulated at 20 min with no change at 60 min. ^c^SA2459, SA2460, and SA2461 were downregulated after 20 min and upregulated after 60 min exposure.

Additional amino acid biosynthesis genes including: SA1164 (*dho*M)-SA1165 (*thr*C)-SA1166 (*thr*B) involved in threonine biosynthesis were also in this group (table 1). Further, SA2082–SA2083–SA2084–SA2085–SA2086–SA2088 (*ure*ABCEFD), which make CO_2 _and NH_3 _from urea and encode urea amidohydrolase and urease accessory proteins and SA1544 which codes for serine-pyruvate aminotransferase were downregulated at 20 and 60 min (table 1).

Group IV of table 1 also shows that the functional class of "cell envelope biogenesis, outer membrane" was distinctive. In particular, SA1231 which shows an -18-fold decrease after 20 min encodes an alanine racemase that catalyses the conversion of L-alanine into D-alanine, a key component of bacterial peptidoglycan [34]. Additionally, the putative lipoproteins: SA0229 (*dpp*A), SA1213 (*opp-*2C)-SA1214 (*opp-*2B), SA1659 (*prs*A), SA2235 (*opu*CC), and SA2409 which are cell wall anchoring surface proteins were downregulated in response to OPP (table 1).

In group IV, we also observed genes related to primary metabolism that mainly belonged to the functional classes of "energy metabolism", "lipid metabolism", and "transcription". For example, cytochrome *bd *complex: SA0937–SA0938 (*cyd*AB) was downregulated upon both 20 min and 60 min exposure (table 1). Cytochrome *bd *complex is one of two terminal oxidases in the bacterial respiratory chain that reduce molecular oxygen to water, utilizing intermediates shuttled through the electron transport chain [35]. Cytochrome *d *oxidase catalyses the last step of oxygen respiration and prevails under oxygen-limiting conditions [36]. Interestingly, it was speculated that cytochrome *d *oxidase is required under conditions of environmental stress and may have crucial roles in cellular physiology other than acting as an oxidase [37]. However, prior studies revealed that the *cyd*AB genes were strongly upregulated upon exposure to hydrogen peroxide strengthens the confidence of the prior assignments about the role of cytochrome *d *oxidase in oxidative protection processes of both Gram positive and Gram negative bacteria [10,13].

### Group V: genes downregulated upon 20 min exposures

In group V in table 1, the most dominant class was "amino acid transport and metabolism", which contained half of the genes in that group. Further, SA1858 (*ilv*D)-SA1859 (*ilv*B) and SA1861 (*ilv*C)-SA1862 (*leu*A)-SA1863 (*leu*B)-SA1864 (*leu*C)-SA1865 (*leu*D)-SA1866 (*ilv*A) which were downregulated (table 1) are possibly parts of an operon homologous to the *ilv*-*leu *operon encoding enzymes of branched-chain amino acid biosynthesis in *Bacillus subtilis *[38]. In addition, SA2464 (*his*I)-SA2465 (*his*F)-SA2466–SA2467 (*his*H)-SA2468 (*his*B)-SA2469 and SA2472 (*his*Z) which are possibly parts of an operon homologous to the histidine biosynthesis were highly downregulated on 20 min (table 1). SA1200–SA1201 (*trp*D)-SA1202 (*trp*C)-SA1203 (*trp*F)-SA1204 (*trp*B)-AS1205 (*trp*A) which are also possibly parts of an operon homologous to the tryptophan biosynthesis operon are downregulated on 20 min (table 1). This result along with the downregulation of 46 genes involved in amino acid biosynthesis in group V suggests that amino acid synthesis was repressed upon 20 min exposure to OPP in *S. aureus*.

Group V shows that 17 genes in the functional class of "inorganic ion transport and metabolism" were downregulated at 20 min. First, SA0771 (*met*Q) codes for probable D-methionine-binding lipoprotein (outer membrane lipoprotein 1). The proteins encoded by SA0344 (*met*E)-SA0769 (*met*N)-SA0770 (*met*I)-SA0420 (*met*N)-SA0421 (*met*I) are involved in D-methionine transporter of *S. aureus *ABC transporter (table 1). Interestingly, group V contained lipoproteins such as SA0010, SA0331, SA0849, SA0850, SA0201 and SA1211 (table 1). These results, along with downregulation of all the genes of lipoproteins of *S. aureus *in group IV and V, suggest that OPP exposure may decrease stability of the staphylococcal membrane. Secondly, the proteins encoded by SA2475 (*cbi*Q)-SA2476 (*cbi*O) are involved in cobalt and nickel transport (table 1).

### Group VI: genes downregulated upon 60 min exposures

Table 1 illustrates that the functional classes of group VI in general contained more downregulated genes at 60 min. In particular, the functional classes of "cell envelope biogenesis outer membrane", "carbohydrate transport and metabolism", "amino acid transport and metabolism", "coenzyme metabolism", "energy production and conversion" and "posttranslational modification protein turnover chaperones" had significantly more downregulated genes at 60 min (see also figure 4). This result suggests that the functional class profiles were notably different between 20 and 60 min.

One of the characteristics of group VI of table 1 was the downregulation of 12 genes belonging to the functional class of "cell wall/membrane/envelope biogenesis". In particular, genes related to envelope biogenesis were distinctive: SA0144 (*cap*A)-SA0145 (*cap*B)-SA0146 (*cap*C)-SA0147 (*cap*D)-SA0148 (*cap*E)-SA0149 (*cap*F)-SA0150 (*cap*G)-SA0151 (*cap*H)-SA0152 (*cap*I)-SA0154 (*cap*K) were downregulated at 60 min. These genes share homology with the capsular polysaccharide synthesis enzyme (*cap*) operon which in turn is essential for virulence by impeding phagocytosis [39]. This finding is congruent with the previous outcome that triclosan downregulates several virulence factor-related genes (SA0144–SA0153 (*cap*ABCDEFGHIJ)) in *S. aureus *[12]. Moreover, IsdAC encoded by SA0977 and SA0978, the iron-regulated surface determinant (Isd) system, was downregulated at 60 min (table 1). Identification of the Isd system in *S. aureus *has demonstrated the importance of cell-wall sorted proteins in heme binding and transport [40]. To date, the Isd system comprises the only known heme-iron utilization pathway in *S. aureus*. Cell-wall sorted proteins of the *S. aureus *iron-regulated surface determinant system bind human hemoproteins, remove the heme molecule, and transport heme through the cell wall and plasma membrane for accumulation in the bacterial cytoplasm [41].

Particularly important was that many of the genes in the class of "coenzyme metabolism" were also members of group VI (figure 4 and table 1). Intriguingly, the genes were all involved in the riboflavin biosynthesis. SA1586 (*rib*H)-SA1587 (*rib*A)-Sa1588 (*rib*B)-SA1589 (*rib*D) was downregulated at 60 min exposure (table 1). Riboflavin (vitamin B2) is an essential component of the basic metabolism, being a precursor of coenzymes flavin adenine dinucleotide (FAD) and flavin mononucleotide (FMN). The best studied system of the riboflavin biosynthesis in bacteria is the *rib *operon of *Bacillus subtilis *encoding a pyrimidine deaminase/reductase, α-subunit of riboflavin synthase, GTP cyclohydrolase/3,4-dihydroxy 2-butanone 4-phosphate (3,4-DHBP) synthase, and β-subunit of riboflavin synthase [42]. These enzymes form a pathway that creates one riboflavin molecule from one molecule of GTP and two molecules of ribulose 5-phosphate [43]. The proteins encoded by SA1894 (*thi*E)-SA1895 (*thi*M)-SA1896 (*thi*D) were involved in thiamine biosynthesis of coenzyme metabolism at 60 min (table 1). Methicillin-resistant *S. aureus *small colony variants are frequently auxotrophic for hemin, menadione, thiamine, and CO_2 _involved in biosynthesis of the electron transport chain element. This phenotype grows slowly, and forms very small, nonhemolytic colonies in routine culture, so it may lead to the misidentification of this organism. As discussed above, group IV also had SA2149 (*hrt*A)-SA2150 (*hrt*B), which exhibited expression level decreases upon 20 and 60 min exposures in chorus with the repression of the genes of thiamine biosynthesis. Therefore, this result suggests that growth inhibition was accompanied with the repression of many coenzyme metabolism-related genes.

### Group VII: genes downregulated upon 20 min and upregulated upon 60 min exposures

Note that group VII has been included only in table 1 in order to discuss the aberrant behaviour of the *ica *genes. Group VII is not indicated on figure 3 and 4. Intriguingly, we observed that SA2459, SA2460 and SA2461 (*ica*ADB) which make up the intercellular adhesion (*ica*) operon and contribute to virulence in *S. aureus *were downregulated after 20 min and upregulated after 60 min of exposure to OPP (table 1 and table 2). The intercellular adhesion operon (*ica*RADBC) mediates polysaccharide intercellular adhesion in *S. aureus*, which leads to cell-cell adhesion and is required for biofilm formation [44] Prior studies have demonstrated that polysaccharide intercellular adhesin/hemagglutinin production is involved in the pathogenesis of *S. epidermidis *[45,46], and is also upregulated by subinhibitory concentrations of certain antibiotics [47]. Therefore, this finding proposes that biofilm formation may not occur after 20 min of exposure to OPP but is possibly favoured as a protective response as exposure time increases to 60 minutes.

### Validation of array data by real-time PCR

As an independent measure of differential gene expression, we examined the relative levels of 18 genes with a range of fold changes (-265.0- to 24.5-fold) by real-time PCR analysis, which were specifically involved in the pathogenesis or metabolism of *S. aureus*. Table 2 shows that our microarray results were corroborated with real-time PCR analysis, which provides independent verification of transcript level changes of the genes that we discuss in this report.

## Conclusion

In this paper, we demonstrated how OPP upregulated and downregulated genes in *S. aureus*, for the first time, by utilizing whole-genome microarrays. Moreover, we presented how the transcriptome profile of *S. aureus *was shifted during its cellular response to OPP, which involved the growth inhibition. To our knowledge, this is the first study demonstrating the activation of fermentative metabolism after OPP treatment in *S. aureus*. In summary, we revealed that amino acid metabolism genes were selectively downregulated between 20 and 60 minutes when exposed to 0.82 mM OPP. We also found that the growth inhibition was accompanied by the downregulation of many membrane function-related genes; however, the majority of these genes returned to normal transcription levels during the growth resumption. Further, we showed that the repression of the iron-regulated surface determinant (Isd) system, hemin and thiamine-related genes accompanied with the growth inhibition. Notably, we discovered the upregulation of virulence genes and ribosomal genes while the cells returned to normal growth. These results suggest that *S. aureus *might be arrested upon exposure to OPP.

In this study, OPP treatment led to the downregulation of several genes involved in amino acid anabolism. The genes involved in the DAP and lysine biosynthetic pathways were most significantly downregulated. Lysine and DAP are essential for building up the peptidoglycan cell wall. This finding proposes that the mode of action of the antimicrobial, OPP in *S. aureus *might be attributed to the inhibition of genes of lysine biosynthesis and subsequently peptidoglycan biosynthesis. We can therefore, conclude, that the mode of action of OPP is similar to the mechanism of action of some antibiotics. This study has revealed novel information on the mechanism of action of OPP in *S. aureus *which will benefit further antimicrobial research on *S. aureus*.

## Methods

### Bacterial strains and growth conditions

In this study, we used *S. aureus *NCTC 8325 obtained from the Network on Antimicrobial Resistance in *S. aureus *(NARSA). As previously described [10-12], we initiated and maintained *S. aureus *cultures at 37°C with shaking at 250 rpm using sterilized Lurria-Bertani (LB) broth. For growth inhibition, 0.14 mg/L (0.82 mM) of OPP (Aldrich Chemical Co., St. Louis, MO) was dissolved in DMSO and used for the microarray study and added immediately after OD_600 _reached 0.8. OD_600 _was measured by using Lambda 25 spectrophotometer (PerkinElmer, Inc., MA). Note that the pH of *S. aureus *cultures was around 7.0 at 37°C after the exposure [48].

### RNA isolation

Total RNA was isolated after 20 and 60 min incubation with and without (control) OPP using the RiboPure – Bacteria kit (Ambion, Inc., Austin, TX) [11]. The quantity of eluted RNA was determined using the NanoDrop spectrophotometer (NanoDrop Technologies, Inc., Wilmington, DE). RNA 6000 Nano LabChip with an Agilent 2100 Bioanalyzer (Agilent Technologies, Palo Alto, CA).

### cDNA synthesis, labeling, hybridization, staining, and scanning

cDNA synthesis, cDNA fragmentation, labeling, hybridization, staining and washing steps were performed according to the manufacturer's protocol for the Affymetrix *S. aureus *GeneChip arrays (Affymetrix, Inc., Santa Clara, CA).

### Affymetrix *S. aureus *genechip analysis

The arrays were scanned with the Affymetrix GeneChip Scanner 3000. To analyze the array data, GeneChip Operating Software (GCOS) v. 1.2 (Affymetrix, Inc., Santa Clara, CA) and GeneSpring GX v. 7.3 (Agilent Technologies, Inc., Santa Clara, CA) were utilized with the following parameters: *alpha *1, 0.04; *alpha *2, 0.06; *tau*, 0.015; target signal, 500. Fold changes were calculated as the ratio between the signal averages of five biological controls (untreated) and five biological experimental (OPP-treated) for 20 and 60 min exposures.

### Real-time PCR analysis

To determine the validity of the array data, transcript level changes obtained with the microarray analysis were compared with those from quantitative real-time PCR. Genes and primer sequences employed for the real-time PCR analysis are listed in table 2. The housekeeping gene, glyceraldehyde 3-phosphate dehydrogenase (GAPDH), was used as an endogenous control. The real-time PCR was performed by employing iCycler iQ Real-Time PCR Detection System with iScript cDNA Synthesis Kit and IQ SYBR Green Supermix (BioRad Laboratories, Inc., Hercules, CA). For each gene, three biological replicates with three technical replicates each were employed. Reaction mixtures were initially incubated for 3 min at 95.0°C, followed by 40 cycles of 10 s at 95.0°C, 30 s at 55.0°C, and 20 s at 72.0°C. PCR efficiencies were derived from standard curve slopes in the iCycler software v. 3.1 (BioRad Laboratories, Inc., Hercules, CA). Melt-curve analysis was also performed to evaluate PCR specificity and resulted in single primer-specific melting temperatures. In this report, relative quantification based on the relative expression of a target gene versus GAPDH gene was utilized to determine transcript level changes.

## Authors' contributions

HJ performed microarray experiments, and data analysis, and drafted the manuscript. FT initiated and supervised the study, and reviewed the manuscript. CN and WEB reviewed the manuscript.

## Supplementary Material

Additional file 1 **The raw data of 7,775 genes control (0 min) and experimental (after 20 and 60 min. exposure of OPP)**. It has been also deposited in NCBI's Gene Expression Omnibus  and is accessible through GEO Series accession number GSE10605 . Click here for file

Additional file 2 ***Staphylococcus aureus *669 genes that showed statistically significant mRNA level changes upon either 20 or 60 min exposure to OPP**. The genes were grouped based on their regulation directions upon 20 and 60 min exposures. Click here for file
