# Supplementary material for: Microarray analysis of toxicogenomic effects of Ortho-phenylphenol in Staphylococcus aureus
Source: BMC Genomics. 2008 Sep 15;9:411. doi: 10.1186/1471-2164-9-411 (PMC2562396; doi:10.1186/1471-2164-9-411)
Supplement: Additional file 2 — Staphylococcus aureus 669 genes that showed statistically significant mRNA level changes upon either 20 or 60 min exposure to OPP. The genes were grouped based on their regulation directions upon 20 and 60 min exposures. [file 1471-2164-9-411-S2.doc]

### Additional file 2 – *Staphylococcus aureus* 669 genes that showed statistically significant mRNA level changes upon either 20 or 60 min exposure to OPP. The genes were grouped based on their regulation directions upon 20 and 60 min exposures.

|  |  | 20 min | | 60 min | |  |  |  |
| --- | --- | --- | --- | --- | --- | --- | --- | --- |
| Affymetrix Probe ID | ORF no. | p-value | Fold change | p-value | Fold change | Description | *Gene symbol* | Functional class |
|  |  |  |  |  |  |  |  |  |
| **Group I: Upregulation (20min) - Upregulation (60 min) 56 genes** | | | | | | |  |  |
| sa_c9748s8493_a_at | SA1271 | 0.00212 | 2.528 | 0.00212 | 2.763 | Threonine dehydratase biosynthetic (Threonine deaminase) |  | Amino acid transport and metabolism |
| sa_c5246s4544_a_at | SA2135 | 0.00154 | 2.744 | 0.00154 | 2.208 | Sodium:glutamate symport carrier protein (Glutamate permease) |  | Amino acid transport and metabolism |
| sa_c4765s4076_a_at | SA2008 | 0.00609 | 2.285 | 0.00609 | 2.028 | Acetolactate synthase isozyme III large subunit (AHAS-III) | *alsS* | Amino acid transport and metabolism, Coenzyme metabolism |
| sa_c7144s6255_a_at | SA0354 | 1.40E-05 | 3.818 | 1.40E-05 | 2.509 | Ribosomal_S18, Ribosomal protein S18 |  | Carbohydrate transport and metabolism |
| sa_c6812s5946_a_at | SA0265 | 8.55E-06 | 2.138 | 8.55E-06 | 3.052 | peptidoglycan hydrolase (surface antigen) | *lyt M* | Cell envelope biogenesis, outer membrane |
| sa_c7698s6703_a_at | SA0519 | 0.00293 | 2.81 | 0.00293 | 2.454 | Ser-Asp rich fibrinogen-binding, bone sialoprotein-binding protein | *sdrC* | Cell envelope biogenesis, outer membrane |
| sa_c592s9345_a_at | SA0905 | 4.83E-08 | 5.742 | 4.83E-08 | 6.981 | N-acetylglucosaminidase (major autolysin) | *atl* | Cell envelope biogenesis, outer membrane |
| sa_c5652s4904_a_at | SA2206 | 0.000815 | 2.525 | 0.000815 | 2.758 | Immunoglobulin G binding protein A precursor | *sbi* | Cell envelope biogenesis, outer membrane |
| sa_c6259s5439_a_at | SA2356 | 3.43E-06 | 6.465 | 3.43E-06 | 6.936 | immunodominant antigen A | *isaA* | Cell envelope biogenesis, outer membrane |
| sa_c6427s5598_a_at | SA2408 | 9.32E-05 | 2.525 | 9.32E-05 | 2.436 | choline-glycine betaine transporter | *cudT* | Cell envelope biogenesis, outer membrane |
| sa_c8206s7186_a_at | SA0666 | 0.000517 | 2.525 | 0.000517 | 3.894 | Putative 6-pyruvoyl tetrahydrobiopterin synthase (PTPS) (PTP synthase) |  | Coenzyme metabolism |
| sa_c4309s3661_a_at | SA1885 | 0.000568 | 2.43 | 0.000568 | 2.031 | Cold-shock DEAD-box protein A homolog (ATP-dependent RNA helicase deaD homolog) |  | DNA replication, recombination and repair, Transcription, and Translation, ribosomal structure and biogenesis |
| sa_c5522s4780_a_at | SA2172 | 2.16E-06 | 2.345 | 2.16E-06 | 3.276 | Excitatory amino acid transporter 1 (Sodium-dependent glutamate:aspartate transporter) | *gltT* | Energy production and conversion |
| sa_c525s350_at | SA0890 | 0.000282 | 2.239 | 0.000282 | 4.779 | hypothetical protein predicted by GeneMark |  | Function unknown |
| sa_c3621s3099_a_at | SA1664 | 1.81E-05 | 2.2 | 1.81E-05 | 2.967 | hypothetical protein predicted by GeneMark |  | Function unknown |
| sa_c7333s6374_a_at | SA0410 | 0.000568 | 2.875 | 0.000568 | 4.107 | cobalamin synthesis related protein CobW |  | General function prediction only |
| sa_c7382s10191_a_at | SA0423 | 5.16E-07 | 8.071 | 5.16E-07 | 19.36 | hypothetical protein, similar to autolysin (N-acetylmuramoyl-L-alanine amidase) |  | General function prediction only |
| sa_c7467s6491_a_at | SA0445 | 1.83E-06 | 2.877 | 1.83E-06 | 2.212 | SAM-dependent methyltransferase |  | General function prediction only |
| sa_c8045s7032_at | SA0620 | 1.27E-06 | 7.618 | 1.27E-06 | 5.641 | hypothetical protein, similar to secretory antigen precursor SsaA |  | General function prediction only |
| sa_c8938s7854_at | SA0667 | 0.000629 | 2.485 | 0.000629 | 4.594 | putative ATPase, confers aluminum resistance |  | General function prediction only |
| sa_c5066s4362_a_at | SA2093 | 0.000246 | 6.786 | 0.000246 | 8.962 | hypothetical protein, similar to secretory antigen precursor SsaA | *ssaA* | General function prediction only |
| sa_c5082s4380_a_at | SA2097 | 3.71E-05 | 6.895 | 3.71E-05 | 11.49 | hypothetical protein, similar to secretory antigen precursor SsaA |  | General function prediction only |
| sa_c5199s4501_a_at | SA2126 | 2.66E-06 | 3.553 | 2.66E-06 | 3.155 | DNA topoisomerase IV subunit A |  | General function prediction only |
| sa_c6151s5333_a_at | SA2332 | 0.000192 | 7.789 | 0.000192 | 8.646 | hypothetical protein, similar to secretory antigen precursor SsaA |  | General function prediction only |
| sa_c6250s5428_a_at | SA2353 | 1.91E-05 | 10.23 | 1.91E-05 | 13.8 | secretory antigen precursor SsaA homolog |  | General function prediction only |
| sa_c904s700_a_at | SA0100 | 0.000405 | 3.866 | 0.000405 | 4.461 | Renal sodium-dependent phosphate transport protein 2 |  | hypothetical protein |
| sa_c7491s6511_x_at | SA0452 | 0.000291 | 2.038 | 0.000291 | 2.105 | hypothetical protein veg |  | hypothetical protein |
| sa_c6849s5983_a_at | SA0268 | 0.000583 | 3.472 | 0.000583 | 2.388 | Conserved hypothetical ORF |  | hypothetical protein |
| sa_c6853s5989_a_at | SA0269 | 9.74E-06 | 4.955 | 9.74E-06 | 3.657 | Cylicin I (Multiple-band polypeptide I) |  | hypothetical protein |
| sa_c7756s6760_a_at | SA0536 | 0.000564 | 2.332 | 0.000564 | 2.669 | Conserved hypothetical ORF |  | hypothetical protein |
| sa_c8928s7841_a_at | SA0651 | 3.13E-07 | 3.692 | 3.13E-07 | 7.447 | Conserved hypothetical ORF |  | hypothetical protein |
| sa_c8469s7429_a_at | SA0751 | 3.53E-05 | 2.316 | 3.53E-05 | 2.077 | Conserved hypothetical ORF |  | hypothetical protein |
| sa_i7490dr_x_at | SA0775 | 9.93E-06 | 2.022 | 9.93E-06 | 2.186 | reverse complement of intergenic downstream of ORF sa_c8532s7490 |  | hypothetical protein |
| sa_c8720s7662_a_at | SA0830 | 0.000837 | 2.267 | 0.000837 | 2.841 | hypothetical protein predicted by GeneMark |  | hypothetical protein |
| sa_c517s346_a_at | SA0889 | 0.000154 | 2.191 | 0.000154 | 3.335 | Conserved hypothetical ORF |  | hypothetical protein |
| sa_c1007s793_a_at | SA1003 | 7.81E-06 | 3.664 | 7.81E-06 | 4.201 | Fibrinogen-binding protein precursor |  | hypothetical protein |
| sa_c3471s3000_a_at | SA1616 | 0.0107 | 2.149 | 0.0107 | 2.123 | Conserved hypothetical ORF |  | hypothetical protein |
| sa_c3485s3012_a_at | SA1619 | 1.24E-06 | 7.676 | 1.24E-06 | 6.127 | Conserved hypothetical ORF |  | hypothetical protein |
| sa_c3487s9161_a_at | SA1620 | 0.00684 | 2.786 | 0.00684 | 2.497 | Conserved hypothetical ORF |  | hypothetical protein |
| sa_c4394s3743_a_at | SA1898 | 7.99E-05 | 6.116 | 7.99E-05 | 5.817 | hypothetical protein, simialr to SceD precursor |  | hypothetical protein |
| sa_c10171s8867_at | SA1944 | 1.05E-05 | 2.13 | 1.05E-05 | 2.304 | Conserved hypothetical ORF |  | hypothetical protein |
| sa_c4612s9984cs_s_at | SA1972 | 0.00267 | 2.209 | 0.00267 | 2.657 | multidrug resistance protein (efflux transporter) |  | hypothetical protein |
| sa_c342s182_a_at | SA2142 | 9.36E-07 | 3.583 | 9.36E-07 | 3.562 | multidrug resistance protein B (drug efflux transporter) |  | hypothetical protein |
| sa_c5274s4572_a_at | SA2143 | 1.24E-07 | 3.853 | 1.24E-07 | 4.077 | multidrug resistance efflux pump |  | hypothetical protein |
| sa_c9402s8223_a_at | SA2355 | 3.23E-06 | 8.537 | 3.23E-06 | 9.354 | TRANSCRIPTIONAL REGULATOR, MARR FAMILY |  | hypothetical protein |
| sa_c6641s5799_a_at | SA2450 | 0.000392 | 2.06 | 0.000392 | 2.41 | Conserved hypothetical ORF |  | hypothetical protein |
| sa_c2626s2200_a_at | SA1384 | 0.000763 | 2.141 | 0.000763 | 2.089 | ABC transporter membrane-spanning permease - Zinc (Zn2+) transport |  | Inorganic ion transport and metabolism |
| sa_c2630s2204_a_at | SA1385 | 0.00113 | 2.56 | 0.00113 | 2.287 | ATP-binding protein MtsB, ABC transporter (ATP-binding protein) |  | Inorganic ion transport and metabolism |
| sa_c9605s8367_a_at | SA1104 | 1.74E-05 | 2.96 | 1.74E-05 | 2.28 | CTP:phosphatidate cytidylyltransferase 2 | *cdsA* | Lipid metabolism |
| sa_c7261s6323_a_at | SA0374 | 0.00089 | 2.452 | 0.00089 | 2.165 | uracil permease (uracil transporter) | *pbuX* | Nucleotide transport and metabolism |
| sa_i10498dr_x_at | SA1172 | 1.31E-06 | 3.257 | 1.31E-06 | 2.018 | reverse complement of intergenic downstream of ORF sa_c10045s10498 |  | Nucleotide transport and metabolism |
| sa_c8202s7182_a_at | SA0665 | 0.000446 | 2.51 | 0.000446 | 4.175 | coenzyme PQQ synthesis homologue |  | Posttranslational modification, protein turnover, chaperones |
| sa_c6506s5675_a_at | SA2423 | 0.00148 | 2.879 | 0.00148 | 3.457 | fibrinogen-binding protein A, clumping factor | *clfB* | Posttranslational modification, protein turnover, chaperones |
| sa_c831s632_a_at | SA0959 | 2.42E-05 | 2.99 | 2.42E-05 | 2.091 | GTP-binding translation elongation factor homolog TypA:BipA |  | Signal transduction mechanisms |
| sa_c1294s1067_a_at | SA1082 | 0.000107 | 2.792 | 0.000107 | 2.388 | RIMM_CHLTE Probable 16S rRNA processing protein rimM |  | Translation, ribosomal structure and biogenesis |
| sa_c1300s1073_a_at | SA1083 | 0.000727 | 2.631 | 0.000727 | 2.397 | tRNA (Guanine-N(1)-)-methyltransferase (M1G-methyltransferase) (tRNA [GM37] methyltransferase) | *trmD* | Translation, ribosomal structure and biogenesis |
|  |  |  |  |  |  |  |  |  |
| **Group II: Upregulation (20min) - No change (60 min) 94 genes** | | | | | | |  |  |
| sa_c1159s942_a_at | SA1045 | 0.000161 | 5.136 |  |  | Carbamoyl-phosphate synthase, arginine-specific, small chain (Arginine-specific carbamoyl-phosphate synthetase, glutamine chain) (CPS-A) | *pyrAA* | Amino acid transport and metabolism, Nucleotide transport and metabolism |
| sa_c1165s946_a_at | SA1046 | 0.00018 | 3.331 |  |  | Carbamoyl-phosphate synthase, arginine-specific, large chain (Arginine-specific carbamoyl-phosphate synthetase, ammonia chain) | *carB* | Amino acid transport and metabolism, Nucleotide transport and metabolism |
| sa_c1536s1314_a_at | SA1150 | 0.000917 | 2.019 |  |  | glutamine synthetase (glutamate-ammonia ligase); glutamine synthetase (glutamate--ammonia ligase) | *glnA* | Amino acid transport and metabolism |
| sa_i8493d_x_at | SA1270 | 0.00491 | 2.126 |  |  | intergenic downstream of ORF sa_c9748s8493 |  | Amino acid transport and metabolism |
| sa_c6088s5276_a_at | SA2318 | 2.22E-05 | 2.276 |  |  | Probable L-serine dehydratase, alpha chain (L-serine deaminase) (SDH) (L-SD) |  | Amino acid transport and metabolism |
| sa_c6092s5283_a_at | SA2319 | 6.44E-05 | 2.475 |  |  | Probable L-serine dehydratase, beta chain (L-serine deaminase) (SDH) (L-SD) |  | Amino acid transport and metabolism |
| sa_c6096s5287_a_at | SA2320 | 1.25E-05 | 2.611 |  |  | regulatory Clostridium protein PfoR |  | Carbohydrate transport and metabolism |
| sa_c8169s7149_a_at | SA0654 | 0.00226 | 2.22 |  |  | Fructose-1-phosphate kinase and related fructose-6-phosphate kinase (PfkB) | *fruB* | Carbohydrate transport and metabolism |
| sa_c4523s3869_a_at | SA1945 | 8.39E-05 | 2.042 |  |  | MANNOSE-6-PHOSPHATE ISOMERASE (PHOSPHOMANNOSE ISOMERASE) (PMI) (PHOSPHOHEXOMUTASE) |  | Carbohydrate transport and metabolism |
| sa_c1139s920_a_at | SA1039 | 3.79E-05 | 2.509 |  |  | Lipoprotein signal peptidase (Prolipoprotein signal peptidase) (Signal peptidase II) (SPase II) | *lsp* | Cell envelope biogenesis, outer membrane |
| sa_c10649s11102cv_s_at | SA1183 | 0.000272 | 2.36 |  |  | Choline-glycine betaine transporter and EctP protein | *opuD* | Cell envelope biogenesis, outer membrane |
| sa_c9023s7924_a_at | SA0796 | 0.000234 | 2.041 |  |  | undecaprenol-phosphate-poly(glycerophosphate chain) D-alanine transfer protein dltD | *dltD* | Cell envelope biogenesis, outer membrane |
| sa_c2964s2521_a_at | SA1487 | 0.000209 | 2.011 |  |  | multifunctional folylpolyglutamate synthase; dihydrofolate synthase, also has formylTHF polyglutamate synthase activity | *folC* | Coenzyme metabolism |
| sa_c2887s2452_a_at | SA1462 | 0.00105 | 2.247 |  |  | SINGLE-STRANDED-DNA-SPECIFIC EXONUCLEASE RECJ (recJ) |  | DNA replication, recombination and repair |
| sa_c1643s1381_at | SA1160 | 0.000799 | 2.134 |  |  | Thermonuclease precursor (TNase) (Micrococcal nuclease) (Staphylococcal nuclease) |  | DNA replication, recombination and repair |
| sa_c7471s6495_at | SA0446 | 2.86E-05 | 2.096 |  |  | Hypothetical 12.2 kDa protein in VLF1-GP41 intergenic region |  | DNA replication, recombination and repair |
| sa_c2120s1823_a_at | SA1277 | 0.000151 | 2.043 |  |  | Predicted N6-adenine-specific DNA methylase |  | DNA replication, recombination and repair |
| sa_c8677s7626_a_at | SA0820 | 0.000463 | 3.529 |  |  | putaitve glycerophophosdyl diester phosphodiesterase (fragment) (putative secreted protein); putative glycerophosphoryl diester phosphodiesterase (fragment) (putative secreted protein) | *glpQ* | Energy production and conversion |
| sa_c31s28_a_at | SA0008 | 0.00747 | 3.454 |  |  | histidine ammonia-lyase; Histidine ammonia-lyase (histidase) | *hutH* | Energy production and conversion |
| sa_c2497s2076_a_at | SA1347 | 0.00106 | 2.086 |  |  | 2-oxoisovalerate dehydrogenase beta subunit (Branched-chain alpha-keto acid dehydrogenase E1 component beta chain) (BCKDH E1-beta) | *bfmBAB* | Energy production and conversion |
| sa_c2493s2074_a_at | SA1346 | 0.000182 | 2.006 |  |  | Dihydrolipoamide branched chain transacylase (E2 component of branched chain keto acid dehydrogenase complex) | *bmfBB* | Energy production and conversion |
| sa_c7463s6487_a_at | SA0444 | 0.000165 | 2.028 |  |  | hypothetical protein predicted by GeneMark |  | Function unknown |
| sa_c3028s2585_at | SA1502 | 2.30E-05 | 2.914 |  |  | 50S ribosomal protein L20; ribosomal protein L20 |  | General function prediction only |
| sa_c6135s5319_a_at | SA2329 | 0.00406 | 2.534 |  |  | murein hydrolase exporter |  | General function prediction only |
| sa_c5227s4529_a_at | SA2132 | 0.000921 | 2.212 |  |  | ATP-binding transport protein natA (Na+ ABC transporter) |  | General function prediction only |
| sa_c1908s1632_a_at | SA1224 | 5.31E-06 | 2.143 |  |  | probable ATP-binding component of ABC transporter |  | General function prediction only |
| sa_c4896s4204_a_at | SA2050 | 0.000225 | 2.067 |  |  | uracil permease (uracil transporter) |  | General function prediction only |
| sa_c2112s1816cs_s_at | SA1276 | 5.68E-06 | 2.349 |  |  | Conserved hypothetical ORF |  | hypothetical protein |
| sa_c6847s5978_a_at | SA0267 | 0.0021 | 2.259 |  |  | Conserved hypothetical ORF |  | hypothetical protein |
| sa_c10550s11011_s_at | SA0745 | 0.000551 | 2.21 |  |  | truncated secreted von Willebrand factor-binding protein VWbp |  | hypothetical protein |
| sa_c1011s797_a_at | SA1004 | 0.00111 | 2.107 |  |  | hypothetical protein, similar to fibrinogen-binding protein |  | hypothetical protein |
| sa_c3481s3008_a_at | SA1618 | 0.000893 | 2.103 |  |  | Conserved hypothetical ORF |  | hypothetical protein |
| sa_c1175s958_a_at | SA1049 | 0.00886 | 2.083 |  |  | Conserved hypothetical ORF |  | hypothetical protein |
| sa_c8296s7275_a_at | SA0694 | 0.00664 | 2.032 |  |  | Glutamate-rich protein grpB |  | hypothetical protein |
| sa_c2757s2330_a_at | SA1423 | 0.0145 | 2.014 |  |  | similar to dihydrodipicolinate reductase |  | hypothetical protein |
| sa_c8909s7826_a_at | SA0619 | 0.00152 | 2.12 |  |  | Low-affinity inorganic phosphate transporter 1 |  | Inorganic ion transport and metabolism |
| sa_c9611s8371_a_at | SA1103 | 0.000146 | 2.279 |  |  | UNDECAPRENYL PYROPHOSPHATE SYNTHETASE (UPP SYNTHETASE) (DI-TRANS-POLY-CIS-DECAPRENYLCISTRANSFERASE) (UNDECAPRENYL DIPHOSPHATE SYNTHASE) (UDS) | *uppS* | Lipid metabolism |
| sa_c10332s10718_s_at | SA1099 | 0.000214 | 2.263 |  |  | Ribosomal_S2, Ribosomal protein S2 | *rpsB* | Lipid metabolism |
| sa_c4770s4080_a_at | SA2016 | 0.000141 | 2.003 |  |  | Ribosomal_S9, Ribosomal protein S9:S16 | *rpsI* | Lipid metabolism |
| sa_c65s61_a_at | SA0016 | 9.89E-06 | 2.6 |  |  | Adenylosuccinate synthetase (IMP--aspartate ligase) (AdSS) (AMPSase) | *purA* | Nucleotide transport and metabolism |
| sa_c7257s6317_a_at | SA0373 | 0.000474 | 2.193 |  |  | xanthine phosphoribosyltransferase |  | Nucleotide transport and metabolism |
| sa_c7543s6563_at | SA0468 | 0.00172 | 2.149 |  |  | Hypoxanthine-guanine phosphoribosyltransferase (HGPRT) (HGPRTase) (HPRT A) |  | Nucleotide transport and metabolism |
| sa_c8803s7743_a_at | SA0479 | 0.0153 | 2.264 |  |  | Sodium:nucleoside cotransporter 2 (Na(+):nucleoside cotransporter 2) (Sodium-coupled nucleoside transporter 2) (Concentrative nucleoside transporter 2) (CNT 2) (Sodium:purine nucleoside co-transporter) (SPNT) | *nupC* | Nucleotide transport and metabolism |
| sa_c1147s928_a_at | SA1041 | 0.00142 | 2.792 |  |  | Uracil phosphoribosyltransferase; Pyrimidine operon regulatory protein pyrR |  | Nucleotide transport and metabolism |
| sa_c1151s932_a_at | SA1042 | 0.000143 | 6.655 |  |  | URACIL PERMEASE (URACIL TRANSPORTER) | *pyrP* | Nucleotide transport and metabolism |
| sa_c9991s8687_a_at | SA1043 | 7.66E-05 | 6.54 |  |  | Aspartate carbamoyltransferase catalytic chain (Aspartate transcarbamylase) (ATCase) | *pyrB* | Nucleotide transport and metabolism |
| sa_c1155s937_a_at | SA1044 | 0.000101 | 6.037 |  |  | Dihydroorotase, Dihydroorotase-like | *pryC* | Nucleotide transport and metabolism |
| sa_c1167s950_a_at | SA1047 | 0.000788 | 3.268 |  |  | Orotidine 5-phosphate decarboxylase (OMP decarboxylase) (OMPDCase) (OMPdecase) | *pyrF* | Nucleotide transport and metabolism |
| sa_c9989s8682_a_at | SA1048 | 0.00107 | 3.003 |  |  | Orotate phosphoribosyltransferase (OPRT) (OPRTase) | *pyrE* | Nucleotide transport and metabolism |
| sa_c9613s8375_a_at | SA1101 | 8.91E-06 | 2.082 |  |  | Uridylate kinase (UK) (Uridine monophosphate kinase) (UMP kinase) |  | Nucleotide transport and metabolism |
| sa_c1697s1432_a_at | SA1172 | 1.40E-06 | 2.863 |  |  | guanosine monophosphate reductase 2; guanosine monophosphate reductase isolog; GMP reductase 2; guanosine 5-monophosphate oxidoreductase 2 |  | Nucleotide transport and metabolism |
| sa_c2883s2448_at | SA1461 | 8.47E-05 | 2.102 |  |  | Adenine phosphoribosyltransferase (APRT) | *apt* | Nucleotide transport and metabolism |
| sa_c9829s8568_a_at | SA1921 | 0.000232 | 2.22 |  |  | thymidine kinase 1, soluble; Thymidine kinase-1 | *tdk* | Nucleotide transport and metabolism |
| sa_c474s9402_a_at | SA0879 | 0.00037 | 2.152 |  |  | periplasmic serine protease Do; heat shock protein HtrA | *htrA* | Posttranslational modification, protein turnover, chaperones |
| sa_c8601s7554_a_at | SA0793 | 1.93E-05 | 2.108 |  |  | D-alanine--poly(phosphoribitol)ligase subunit 1 (D-alanine-activating enzyme) (DAE) (D-alanine-D-alanyl carrier protein ligase) (DCL) | *dltA* | Secondary metabolites biosynthesis, transport and catabolism |
| sa_c8607s7564_at | SA0795 | 6.58E-06 | 2.033 |  |  | D-alanine--poly(phosphoribitol)ligase subunit 2 (D-alanyl carrier protein) (DCP) |  | Secondary metabolites biosynthesis, transport and catabolism |
| sa_c7620s6631_a_at | SA0496 | 3.27E-06 | 2.794 |  |  | 50S ribosomal protein L1; ribosomal protein L1 (BL1) | *rplA* | Transcription |
| sa_c1948s1673_at | SA1234 | 0.000924 | 2.266 |  |  | COLD SHOCK PROTEIN CSPB (MAJOR COLD SHOCK PROTEIN) |  | Transcription |
| sa_c1530s1310_at | SA1149 | 9.24E-05 | 2.176 |  |  | transcriptional regulator; glutamine synthetase repressor |  | Transcription |
| sa_c7637s6652_a_at | SA0501 | 0.00199 | 2.038 |  |  | DNA-directed RNA polymerase beta chain (Transcriptase beta chain) (RNA polymerase beta subunit) | *rpoC* | Transcription |
| sa_c4796s4102_at | SA2023 | 2.76E-05 | 2.022 |  |  | DNA-directed RNA polymerase alpha chain (RNAP alpha subunit) (Transcriptase alpha chain) (RNA polymerase alpha subunit) | *rpoA* | Transcription |
| sa_c8164s7148_a_at | SA0653 | 0.000831 | 2.186 |  |  | putative DEOR-type transcriptional regulator of aga operon |  | Transcription, Carbohydrate transport and metabolism |
| sa_c511s337_a_at | SA0085 | 0.00233 | 2.11 |  |  | transcriptional regulator (NifR3:Smm1 family) |  | Translation, ribosomal structure and biogenesis |
| sa_c7511s6531_a_at | SA0459 | 0.000138 | 2.299 |  |  | Ribosomal protein L25 (general stress protein Ctc) | *rplY* | Translation, ribosomal structure and biogenesis |
| sa_c7621s6634_a_at | SA0497 | 7.95E-05 | 2.205 |  |  | 50S ribosomal protein L10; ribosomal protein L10 (BL5) |  | Translation, ribosomal structure and biogenesis |
| sa_c7625s6638_at | SA0498 | 5.07E-05 | 3.472 |  |  | 50S ribosomal protein L7:L12; ribosomal protein L7:L12 |  | Translation, ribosomal structure and biogenesis |
| sa_c8822s7758_at | SA0499 | 7.67E-06 | 3.291 |  |  | Protein methyltransferase hemK (Protein-glutamine N-methyltransferase hemK) (Protein-(glutamine-N5) MTase hemK) (M.StyLTHemKP) |  | Translation, ribosomal structure and biogenesis |
| sa_c1143s924_a_at | SA1040 | 0.000177 | 2.068 |  |  | ribosomal large chain pseudouridine synthase A (pseudouridylate synthase) (uracil hydrolyase) |  | Translation, ribosomal structure and biogenesis |
| sa_c1302s1077_a_at | SA1084 | 0.000221 | 2.698 |  |  | 50S ribosomal protein L19; ribosomal protein L19 | *rplS* | Translation, ribosomal structure and biogenesis |
| sa_c1362s1136_a_at | SA1102 | 2.64E-06 | 2.128 |  |  | Probable ribosome recycling factor (Ribosome releasing factor) (RRF) | *frr* | Translation, ribosomal structure and biogenesis |
| sa_c4460s3803_a_at | SA1919 | 7.30E-05 | 2.725 |  |  | HemK protein, probable protoporphyrinogen oxidase hemK |  | Translation, ribosomal structure and biogenesis |
| sa_c4462s3807_at | SA1920 | 1.83E-05 | 2.468 |  |  | peptide chain release factor 1 in translation prfA | *prfA* | Translation, ribosomal structure and biogenesis |
| sa_c4466s3812_a_at | SA1922 | 0.00133 | 2.111 |  |  | Ribosomal_L31, Ribosomal protein L31 | *rpmE2* | Translation, ribosomal structure and biogenesis |
| sa_c4792s4098_at | SA2022 | 0.000417 | 2.062 |  |  | 50S ribosomal protein L17; ribosomal protein L17 | *rplQ* | Translation, ribosomal structure and biogenesis |
| sa_c4824s4130_a_at | SA2029 | 2.76E-05 | 2.115 |  |  | 50S ribosomal protein L15; ribosomal protein L15 | *rplO* | Translation, ribosomal structure and biogenesis |
| sa_c4828s4134_at | SA2030 | 0.000866 | 2.32 |  |  | Ribosomal_L30, Ribosomal protein L30p:L7e | *rpmD* | Translation, ribosomal structure and biogenesis |
| sa_c4832s4138_at | SA2031 | 0.000227 | 2.221 |  |  | similar to ribosomal protein S2; 40S ribosomal protein S2 | *rpsE* | Translation, ribosomal structure and biogenesis |
| sa_c4836s4142_at | SA2032 | 0.00024 | 2.087 |  |  | 50S ribosomal protein L18; ribosomal protein L18 | *rplR* | Translation, ribosomal structure and biogenesis |
| sa_c9951s8647_at | SA2033 | 0.000342 | 2.582 |  |  | 50S ribosomal protein L6; ribosomal protein L6 (BL8) | *rplF* | Translation, ribosomal structure and biogenesis |
| sa_c4840s4147_a_at | SA2034 | 0.000182 | 2.574 |  |  | Ribosomal_S8, Ribosomal protein S8 | *rpsH* | Translation, ribosomal structure and biogenesis |
| sa_c4848s4156_at | SA2035 | 0.0021 | 2.133 |  |  | 50S ribosomal protein L5; ribosomal protein L5 (BL6) | *rpsN* | Translation, ribosomal structure and biogenesis |
| sa_c4852s4158_at | SA2036 | 0.00267 | 2.081 |  |  | 50S ribosomal protein L24; ribosomal protein L24 (BL23) | *rplX* | Translation, ribosomal structure and biogenesis |
| sa_c9955s8651_a_at | SA2037 | 0.000564 | 2.62 |  |  | ribosomal protein L23; 60S ribosomal protein L23 | *rplN* | Translation, ribosomal structure and biogenesis |
| sa_c10191s8871_a_at | SA2038 | 0.0026 | 2.795 |  |  | 30S ribosomal protein S17; ribosomal protein S17 (BS16) | *rpsQ* | Translation, ribosomal structure and biogenesis |
| sa_c4860s4166_at | SA2039 | 0.00204 | 2.508 |  |  | 50S ribosomal protein L29; ribosomal protein L29 | *rpmC* | Translation, ribosomal structure and biogenesis |
| sa_c4864s4170_at | SA2040 | 0.000749 | 2.676 |  |  | 50S ribosomal protein L16; ribosomal protein L16 | *rplP* | Translation, ribosomal structure and biogenesis |
| sa_c4868s4175_a_at | SA2041 | 0.0024 | 2.828 |  |  | 30S ribosomal protein S3; ribosomal protein S3 (BS3) | *rpsC* | Translation, ribosomal structure and biogenesis |
| sa_c4872s4181_at | SA2042 | 0.0021 | 2.519 |  |  | Ribosomal_L22, Ribosomal protein L22p:L17e | *rplV* | Translation, ribosomal structure and biogenesis |
| sa_c4876s4184_at | SA2043 | 0.00405 | 2.809 |  |  | 30S ribosomal protein S19; ribosomal protein S19 (BS19) | *rpsS* | Translation, ribosomal structure and biogenesis |
| sa_c9959s8654_a_at | SA2044 | 0.000888 | 2.535 |  |  | 50S ribosomal protein L2; ribosomal protein L2 (BL2) | *rplB* | Translation, ribosomal structure and biogenesis |
| sa_c10192s8875_a_at | SA2045 | 0.00434 | 2.689 |  |  | 50S ribosomal protein L23; ribosomal protein L23 | *rplW* | Translation, ribosomal structure and biogenesis |
| sa_c4880s4187_at | SA2046 | 0.00283 | 2.834 |  |  | 50S ribosomal protein L4; ribosomal protein L4 | *rplD* | Translation, ribosomal structure and biogenesis |
| sa_c4888s4195_a_at | SA2047 | 0.00069 | 2.314 |  |  | 50S ribosomal protein L3; ribosomal protein L3 (BL3) | *rplC* | Translation, ribosomal structure and biogenesis |
| sa_c9963s8658_a_at | SA2048 | 0.00245 | 2.388 |  |  | 30S ribosomal protein S10; ribosomal protein S10 (BS13) | *rpsJ* | Translation, ribosomal structure and biogenesis |
|  |  |  |  |  |  |  |  |  |
| **Group III: No change (20 min) - Upregulation (60min) 44 genes** | | | | | | |  |  |
| sa_c7410s6434_a_at | SA0430 |  |  | 0.000217 | 5.444 | Glutamate synthase [NADPH] large chain precursor (Glutamate synthase alpha subunit) (NADPH-GOGAT) (GLTS alpha chain) | *gltB* | Amino acid transport and metabolism |
| sa_c7412s6438_a_at | SA0431 |  |  | 0.00033 | 3.444 | Glutamate synthase [NADPH] small chain (Glutamate synthase beta subunit) (NADPH-GOGAT) (GLTS beta chain) | *gltD* | Amino acid transport and metabolism |
| sa_c8536s7494_a_at | SA0776 |  |  | 3.16E-05 | 2.056 | nifS family enzyme (cysteine desulfurase:cysteine sulfinate desulfinase) |  | Amino acid transport and metabolism |
| sa_c10571s9056_a_at | SA0845 |  |  | 0.000581 | 2.494 | putative oligopeptide ABC transporter integral membrane protein (fragment); putative peptide ABC transporter integral membrane protein (fragment) | *oppB* | Amino acid transport and metabolism, Inorganic ion transport and metabolism |
| sa_c324s166_a_at | SA0846 |  |  | 0.00019 | 2.407 | PROBABLE PEPTIDE ABC TRANSPORTER PERMEASE ABC TRANSPORTER PROTEIN |  | Amino acid transport and metabolism, Inorganic ion transport and metabolism |
| sa_c328s170_a_at | SA0847 |  |  | 0.00293 | 2.188 | PROBABLE PEPTIDE ABC TRANSPORTER ATP-BINDING ABC TRANSPORTER PROTEIN | *oppD* | Amino acid transport and metabolism, Inorganic ion transport and metabolism |
| sa_c332s172_a_at | SA0848 |  |  | 9.13E-05 | 2.171 | PROBABLE ABC TRANSPORTER ATP BINDING ABC TRANSPORTER PROTEIN | *oppF* | Amino acid transport and metabolism |
| sa_c5349s4625_a_at | SA0950 |  |  | 0.0022 | 2.226 | ABC transporter ATP-binding protein - spermidine:putrescine transport |  | Amino acid transport and metabolism |
| sa_c795s596_a_at | SA0952 |  |  | 0.00536 | 2.196 | ABC transporter membrane-spanning permease - spermidine:putrescine transport | *potC* | Amino acid transport and metabolism |
| sa_c803s604_a_at | SA0953 |  |  | 0.00987 | 2.185 | spermidine:putrescine ABC transporter, spermidine: putrescine-binding periplasmic protein (potD) homolog | *potD* | Amino acid transport and metabolism |
| sa_c5638s4893_a_at | SA2202 |  |  | 0.000882 | 2.401 | putative amino acid ABC transporter, periplasmic amino acid-binding protein |  | Amino acid transport and metabolism, Signal transduction mechanisms |
| sa_c1139s920_a_at | SA1039 |  |  | 3.79E-05 | 2.245 | Lipoprotein signal peptidase (Prolipoprotein signal peptidase) (Signal peptidase II) (SPase II) | *lsp* | Cell envelope biogenesis, outer membrane |
| sa_c8848s7783_a_at | SA1601 |  |  | 0.000923 | 2.348 | CRCB, CrcB-like protein |  | Cell division and chromosome partitioning |
| sa_c9442s8255_a_at | SA2459 |  |  | 0.0016 | 2.452 | intercellular adhesion protein IcaA | *icaA* | Cell envelope biogenesis, outer membrane |
| sa_c6677s5830_a_at | SA2460 |  |  | 0.0136 | 4.697 | IcaD | *icaD* | Cell envelope biogenesis, outer membrane |
| sa_c6681s9106_a_at | SA2461 |  |  | 3.83E-06 | 3.421 | intercellular adhesion protein IcaB | *icaB* | Cell envelope biogenesis, outer membrane |
| sa_c2711s2285_a_at | SA1412 |  |  | 0.000428 | 2.106 | Oxygen-independent coproporphyrinogen III oxidase (Coproporphyrinogenase) (Coprogen oxidase) | *hemN* | Coenzyme metabolism |
| sa_c1344s1117_a_at | SA1092 |  |  | 0.000396 | 2.13 | DNA processing protein (Smf family) smf |  | DNA replication, recombination and repair |
| sa_c5070s4366_a_at | SA2094 |  |  | 0.000106 | 2.554 | Na(+):H(+) antiporter (Sodium:proton antiporter) |  | Energy production and conversion |
| sa_c6861s5997_a_at | SA0272 |  |  | 0.00117 | 2.412 | hypothetical protein, similar to transmembrane protein Tmp7 |  | Function unknown |
| sa_c2066s1777_a_at | SA1265 |  |  | 0.0269 | 2.3 | putative integral membrane protein |  | Function unknown |
| sa_c6262s5443_a_at | SA2357 |  |  | 1.29E-05 | 2.645 | hypothetical protein, similar to regulatory protein (pfoS:R) |  | General function prediction only |
| sa_c4628s3951_a_at | SA1975 |  |  | 0.00313 | 2.189 | conserved hypotehtical protein |  | General function prediction only |
| sa_c2647s2221_a_at | SA1389 |  |  | 0.000536 | 2.095 | hypothetical protein predicted by GeneMark |  | General function prediction only |
| sa_c1980s1705_a_at | SA1241 |  |  | 0.0101 | 2.027 | nitric oxide reductase NorQ protein |  | General function prediction only |
| sa_c5005s4307_a_at | SA2077 |  |  | 0.00249 | 2.013 | biotin synthase |  | General function prediction only |
| sa_c8528s7487_a_at | SA0773 |  |  | 0.00134 | 2.997 | predicted membrane protein |  | hypothetical protein |
| sa_c10682s11132_s_at | SA1972 |  |  | 0.000599 | 2.982 | putative multidrug transporter |  | hypothetical protein |
| sa_c446s279_a_at | SA2092 |  |  | 0.00107 | 2.806 | hypothetical protein, similar to transcription regulator |  | hypothetical protein |
| sa_c995s782_a_at | SA1000 |  |  | 0.0265 | 2.798 | hypothetical protein, similar to fibrinogen-binding protein |  | hypothetical protein |
| sa_c336s179_a_at | SA1970 |  |  | 0.000273 | 2.654 | multidrug resistance protein B (drug efflux transporter) |  | hypothetical protein |
| sa_c346s186_a_at | SA2203 |  |  | 0.00106 | 2.359 | multidrug resistance protein (efflux transporter) |  | hypothetical protein |
| sa_c6638s5795_a_at | SA2449 |  |  | 0.00487 | 2.14 | Conserved hypothetical ORF |  | hypothetical protein |
| sa_c10551s9053_a_at | SA0775 |  |  | 1.60E-05 | 2.097 | Iron-regulated ABC-type transporter membrane component (SufB) |  | hypothetical protein |
| sa_c9334s8169_a_at | SA2173 |  |  | 0.000476 | 2.071 | Conserved hypothetical ORF |  | hypothetical protein |
| sa_c7491s6511_at | SA0452 |  |  | 0.000406 | 2.053 | hypothetical protein veg | *veg* | hypothetical protein |
| sa_c5936s5156_a_at | SA0403 |  |  | 0.00061 | 2.044 | hypothetical protein [Pathogenicity island SaPIn2] | *lpl7* | hypothetical protein |
| sa_c3767s3239_at | SA1709 |  |  | 0.00281 | 2.133 | Ferritin, middle subunit (Ferritin M) (Ferritin X) (Ferritin H) |  | Inorganic ion transport and metabolism |
| sa_c9004s7911_at | SA0774 |  |  | 3.65E-05 | 2.014 | Predicted CDS, ABC transporter with ABC transporter transmembrane region family member |  | Posttranslational modification, protein turnover, chaperones |
| sa_c5529s4783_a_at | SA2174 |  |  | 0.000565 | 2.742 | transcriptional regulator OhrR |  | Transcription |
| sa_c5056s4355_a_at | SA2091 |  |  | 0.0011 | 2.449 | iraD |  | Transcription |
| sa_c10502s10951cv_s_at | SA2147 |  |  | 0.0332 | 2.15 | TcaR transcription regulator tcaR |  | Transcription |
| sa_c791s594_at | SA0949 |  |  | 0.00327 | 2.134 | Predicted transcriptional regulator |  | Transcription |
| sa_c1108s889_at | SA0108 |  |  | 0.000543 | 2.111 | staphylococcal accessory regulator A homologue |  | Transcription |
|  |  |  |  |  |  |  |  |  |
| **Group IV: Downregulation (20min) - Downregulation (60 min) 201 genes** | | | | | | |  |  |
| sa_c5061s4360_a_at | SA0229 | 4.85E-07 | -5.75 | 4.85E-07 | -4.76 | dipeptide ABC transporter, periplasmic dipeptide-binding protein (dppA) |  | Amino acid transport and metabolism |
| sa_c6957s6079_a_at | SA0303 | 0.00192 | -2.45 | 0.00192 | -7.58 | human solute carrier family 5, member 3, Sodium:myo-inositol cotransporter |  | Amino acid transport and metabolism |
| sa_c6959s6083_a_at | SA0304 | 0.000882 | -3.50 | 0.000882 | -9.90 | Dihydrodipicolinate synthase:N-acetylneuraminate lyase | *nanA* | Amino acid transport and metabolism, Cell envelope biogenesis, outer membrane |
| sa_c7036s9080_a_at | SA0326 | 2.85E-08 | -12.80 | 2.85E-08 | -7.30 | similar to ABC transporter (binding protein) |  | Amino acid transport and metabolism |
| sa_c7669s6683_a_at | SA0512 | 7.37E-08 | -3.30 | 7.37E-08 | -2.07 | D-alanine aminotransferase (D-aspartate aminotransferase) (D-amino acid aminotransferase) (D-amino acid transaminase) (DAAT) | *ilvE* | Amino acid transport and metabolism, Coenzyme metabolism |
| sa_c8673s7622_a_at | SA0818 | 0.00289 | -2.35 | 0.00289 | -2.76 | Ornithine aminotransferase (Ornithine--oxo-acid aminotransferase) | *rocD* | Amino acid transport and metabolism |
| sa_c574s400_a_at | SA0902 | 1.85E-06 | -4.17 | 1.85E-06 | -2.08 | Aspartate aminotransferase (Transaminase A) (AspAT) |  | Amino acid transport and metabolism |
| sa_c9581s8342_a_at | SA1163 | 4.27E-07 | -17.39 | 4.27E-07 | -3.94 | Lysine-sensitive aspartokinase III (Aspartate kinase III) |  | Amino acid transport and metabolism |
| sa_c1659s1395_a_at | SA1164 | 0.000431 | -8.93 | 0.000431 | -3.38 | Homoserine dehydrogenase (HDH) | *dhoM* | Amino acid transport and metabolism |
| sa_c1665s1401_a_at | SA1165 | 0.00011 | -8.26 | 0.00011 | -3.23 | threonine synthase (EC 4.2.3.1) homolog thrC | *thrC* | Amino acid transport and metabolism |
| sa_c1669s1406_a_at | SA1166 | 5.06E-05 | -11.49 | 5.06E-05 | -3.80 | homoserine kinase (thrB) | *thrB* | Amino acid transport and metabolism |
| sa_c1800s1530_a_at | SA1194 | 0.000634 | -3.79 | 0.000634 | -3.77 | Peptide methionine sulfoxide reductase msrA 1 (Protein-methionine-S-oxide reductase 1) (Peptide Met(O) reductase 1) | *msrA* | Amino acid transport and metabolism |
| sa_c1870s1592_a_at | SA1212 | 0.000109 | -2.70 | 0.000109 | -2.43 | Dipeptide transport ATP-binding protein dppD; Dipeptide transport ATP-binding protein dppF |  | Amino acid transport and metabolism, Inorganic ion transport and metabolism |
| sa_c1872s1598_a_at | SA1213 | 0.00127 | -2.79 | 0.00127 | -2.40 | PROBABLE PEPTIDE ABC TRANSPORTER PERMEASE ABC TRANSPORTER PROTEIN | *opp-2C* | Amino acid transport and metabolism, Inorganic ion transport and metabolism |
| sa_c1876s1602_a_at | SA1214 | 8.58E-06 | -3.65 | 8.58E-06 | -2.95 | putative oligopeptide ABC transporter integral membrane protein (fragment); putative peptide ABC transporter integral membrane protein (fragment) | *opp-2B* | Amino acid transport and metabolism, Inorganic ion transport and metabolism |
| sa_c1912s1635_a_at | SA1225 | 8.90E-06 | -54.64 | 8.90E-06 | -7.69 | aspartokinase II in bifunctional enxyme: aspartokinase II; homoserine dehydrogenase II | *lysC* | Amino acid transport and metabolism |
| sa_c1918s1640_a_at | SA1226 | 1.78E-05 | -21.46 | 1.78E-05 | -4.27 | ASPARTATE-SEMIALDEHYDE DEHYDROGENASE (ASA DEHYDROGENASE) (ASA DH) | *asd* | Amino acid transport and metabolism |
| sa_c1922s1644_a_at | SA1227 | 2.75E-07 | -27.32 | 2.75E-07 | -5.18 | Dihydrodipicolinate synthase (DHDPS) | *dapA* | Amino acid transport and metabolism, Cell envelope biogenesis, outer membrane |
| sa_c1924s1648_a_at | SA1228 | 1.17E-07 | -31.35 | 1.17E-07 | -5.08 | Dihydrodipicolinate reductase (DHPR) | *dapB* | Amino acid transport and metabolism |
| sa_c1928s1652_a_at | SA1229 | 2.55E-07 | -23.47 | 2.55E-07 | -4.39 | 2,3,4,5-tetrahydropyridine-2,6-dicarboxylate N-succinyltransferase (Tetrahydrodipicolinate N-succinyltransferase) (THP succinyltransferase) (Tetrahydropicolinate succinylase) | *dapD* | Amino acid transport and metabolism |
| sa_c1940s1663_at | SA1232 | 7.12E-08 | -5.71 | 7.12E-08 | -2.37 | diaminopimelate decarboxylase (dap decarboxylase) (lysA) | *lysA* | Amino acid transport and metabolism |
| sa_c2473s2053_a_at | SA1343 | 1.35E-05 | -3.23 | 1.35E-05 | -3.42 | Peptidase T (Tripeptide aminopeptidase) (Aminotripeptidase) (Tripeptidase) |  | Amino acid transport and metabolism |
| sa_c10655s11108_s_at | SA1505 | 6.21E-06 | -6.80 | 6.21E-06 | -2.95 | VALINE AMINO-ACID PERMEASE (BRANCHED-CHAIN AMINO-ACID PERMEASE 3) | *lysP* | Amino acid transport and metabolism |
| sa_c3144s2693_a_at | SA1530 | 4.57E-05 | -2.02 | 4.57E-05 | -2.62 | Xaa-Pro dipeptidase (X-Pro dipeptidase) (Proline dipeptidase) (Prolidase) (Imidodipeptidase) (Peptidase 4) |  | Amino acid transport and metabolism |
| sa_c3202s2750_a_at | SA1544 | 2.74E-05 | -15.82 | 2.74E-05 | -3.79 | serine-pyruvate aminotransferase; Alanine-glyoxylate aminotransferase (Serine-pyruvate aminotransferase) |  | Amino acid transport and metabolism |
| sa_c3262s2810_a_at | SA1558 | 2.13E-08 | -5.24 | 2.13E-08 | -2.23 | 2-dehydro-3-deoxyphosphooctonate aldolase (Phospho-2-dehydro-3-deoxyoctonate aldolase) (3-deoxy-D-manno-octulosonic acid 8-phosphate synthetase) (KDO-8-phosphate synthetase) (KDO 8-P synthase) |  | Amino acid transport and metabolism |
| sa_c10591s11046_s_at | SA1814 | 1.99E-05 | -3.33 | 1.99E-05 | -2.86 | SUCCINYL-DIAMINOPIMELATE DESUCCINYLASE (dapE) |  | Amino acid transport and metabolism |
| sa_c4601s3932_a_at | SA1968 | 0.0288 | -2.83 | 0.0288 | -2.79 | arginase 2; arginase type II | *arg* | Amino acid transport and metabolism |
| sa_c5023s4322_at | SA2082 | 6.38E-07 | -3.48 | 6.38E-07 | -5.52 | urease gamma chain (urea amidohydrolase) | *ureA* | Amino acid transport and metabolism |
| sa_c5029s4326_a_at | SA2083 | 2.01E-06 | -4.31 | 2.01E-06 | -7.63 | URE2_STAXY UREASE BETA SUBUNIT (UREA AMIDOHYDROLASE) | *ureB* | Amino acid transport and metabolism |
| sa_c5031s4330_a_at | SA2084 | 1.84E-07 | -3.02 | 1.84E-07 | -4.42 | urease alpha chain (urea amidohydrolase) | *ureC* | Amino acid transport and metabolism |
| sa_c9348s9368_a_at | SA2226 | 0.00765 | -2.54 | 0.00765 | -5.85 | Aromatic amino acid transport protein aroP (General aromatic amino acid permease) |  | Amino acid transport and metabolism |
| sa_c5773s5016_at | SA2234 | 7.30E-06 | -4.24 | 7.30E-06 | -2.99 | probable glycine betaine:carnitine:choline ABC transporter (membrane p) opuCD |  | Amino acid transport and metabolism |
| sa_c5781s5024_a_at | SA2236 | 0.000325 | -6.17 | 0.000325 | -4.26 | glycine betaine:carnitine:choline ABC transporter, permease protein, putative | *opuCB* | Amino acid transport and metabolism |
| sa_c5355s4632_a_at | SA2237 | 1.72E-05 | -7.04 | 1.72E-05 | -4.88 | putative ABC transporter, ATP-binding protein, proline:glycine betaine transport system | *opuCA* | Amino acid transport and metabolism |
| sa_c6046s9222_a_at | SA2307 | 5.33E-06 | -6.33 | 5.33E-06 | -3.47 | hypothetical protein, similar to ABC transporter (binding protein) |  | Amino acid transport and metabolism |
| sa_c6224s5400_a_at | SA2347 | 2.26E-06 | -6.67 | 2.26E-06 | -3.21 | aspartate aminotransferase A (transaminase A) (aspat) |  | Amino acid transport and metabolism |
| sa_c5270s4569_a_at | SA0233 | 3.63E-06 | -8.26 | 3.63E-06 | -9.17 | PTS SYSTEM, GLUCOSE-SPECIFIC IIABC COMPONENT (EIIABC-GLC) (GLUCOSE-PERMEASE IIABC COMPONENT) (PHOSPHOTRANSFERASE ENZYME II, ABC COMPONENT) (EII-GLC:EIII-GLC) [Mycoplasma pulmonis] match=9.88% identical |  | Carbohydrate transport and metabolism |
| sa_c6517s5684_a_at | SA0258 | 1.77E-05 | -2.67 | 1.77E-05 | -6.13 | ribokinase; pfkB family carbohydrate kinase | *rbsK* | Carbohydrate transport and metabolism |
| sa_c6561s5728_a_at | SA0259 | 8.33E-06 | -2.55 | 8.33E-06 | -5.92 | membrane-associated component of D-ribose high-affinity transport system | *rbsD* | Carbohydrate transport and metabolism |
| sa_c9259s8103_a_at | SA0260 | 1.48E-05 | -2.28 | 1.48E-05 | -5.99 | hypothetical protein, similar to ribose transporter RbsU |  | Carbohydrate transport and metabolism |
| sa_c6943s6067_a_at | SA0298 | 7.93E-05 | -3.31 | 7.93E-05 | -2.00 | hypothetical protein, similar to regulatory protein PfoR |  | Carbohydrate transport and metabolism |
| sa_c9189s8052_a_at | SA0307 | 0.000141 | -3.34 | 0.000141 | -6.71 | N-acetylmannosamine-6-phosphate 2-epimerase : N-acetylmannosamine kinase |  | Carbohydrate transport and metabolism |
| sa_c7724s6726_a_at | SA0527 | 9.16E-06 | -2.79 | 9.16E-06 | -2.52 | GLUCOSAMINE-6-PHOSPHATE ISOMERASE (GLUCOSAMINE-6-PHOSPHATE DEAMINASE) (GNPDA) (GLCN6P DEAMINASE)glucosamine | *nagB* | Carbohydrate transport and metabolism |
| sa_c7997s6983_at | SA0605 | 0.000345 | -2.24 | 0.000345 | -4.55 | Dihydroxyacetone kinase (Glycerone kinase) (DHA kinase) |  | Carbohydrate transport and metabolism |
| sa_c2458s2040_a_at | SA1338 | 0.0401 | -2.12 | 0.0401 | -2.33 | Glucan 1,6-alpha-glucosidase (Dextran glucosidase) (Exo-1,6-alpha-glucosidase) (Glucodextranase) | *malA* | Carbohydrate transport and metabolism |
| sa_c5150s4447_a_at | SA2114 | 0.00243 | -2.06 | 0.00243 | -7.81 | PTS system, glucose-specific IIABC component (EIIABC-GLC) (Glucose-permease IIABC component) (Phosphotransferase enzyme II, ABC component) (EII-GLC:EIII-GLC) | *glvC* | Carbohydrate transport and metabolism |
| sa_c5644s4894_a_at | SA2204 | 0.000186 | -2.58 | 0.000186 | -3.33 | Phosphoglycerate mutase 2 (Phosphoglycerate mutase isozyme M) (PGAM-M) (BPG-dependent PGAM 2) (Muscle-specific phosphoglycerate mutase) |  | Carbohydrate transport and metabolism |
| sa_c6036s5235_a_at | SA2304 | 8.29E-05 | -3.10 | 8.29E-05 | -5.29 | fructose-bisphosphatase (EC 3.1.3.11) | *fbp* | Carbohydrate transport and metabolism |
| sa_c6760s5903_a_at | SA2480 | 0.000349 | -3.18 | 0.000349 | -10.37 | Drp35 | *drp35* | Carbohydrate transport and metabolism |
| sa_c4120s3473_a_at | SA0205 | 1.10E-05 | -8.55 | 1.10E-05 | -3.21 | Lysostaphin precursor (Glycyl-glycine endopeptidase) |  | Cell envelope biogenesis, outer membrane |
| sa_c6776s5917_a_at | SA0264 | 5.56E-05 | -2.51 | 5.56E-05 | -2.14 | Choloylglycine hydrolase (Conjugated bile acid hydrolase) (CBAH) (Bile salt hydrolase) |  | Cell envelope biogenesis, outer membrane |
| sa_c7280s6339_a_at | SA0381 | 5.82E-05 | -4.63 | 5.82E-05 | -3.31 | conserved hypothetical protein [Pathogenicity island SaPIn2] |  | Cell envelope biogenesis, outer membrane, Carbohydrate transport and metabolism |
| sa_c8184s7162_a_at | SA0659 | 1.43E-05 | -2.49 | 1.43E-05 | -2.26 | Probable dolichol-phosphate mannosyltransferase (Dolichol-phosphate mannose synthase) (Dolichyl-phosphate beta-D-mannosyltransferase) (Mannose-P-dolichol synthase) (MPD synthase) (DPM synthase) |  | Cell envelope biogenesis, outer membrane |
| sa_c980s770_a_at | SA0997 | 0.000129 | -2.77 | 0.000129 | -2.15 | PROBABLE GLUTAMATE RACEMASE, REQUIRED FOR BIOSYNTHESIS OF D-GLUTAMATE AND PEPTIDOGLYCAN PROTEIN | *murI* | Cell envelope biogenesis, outer membrane |
| sa_c1936s1659_a_at | SA1231 | 8.94E-08 | -18.52 | 8.94E-08 | -3.00 | Ala_racemase, Alanine racemase |  | Cell envelope biogenesis, outer membrane |
| sa_c4747s4060_a_at | SA2004 | 2.90E-05 | -3.11 | 2.90E-05 | -3.26 | Peptidase_M37, Peptidase family M23:M37 |  | Cell envelope biogenesis, outer membrane |
| sa_c9325s8164_a_at | SA2157 | 9.83E-06 | -2.33 | 9.83E-06 | -2.55 | hypothetical protein, similar to teichoic acid biosynthesis protein F |  | Cell envelope biogenesis, outer membrane |
| sa_c5777s5020_a_at | SA2235 | 2.82E-05 | -5.10 | 2.82E-05 | -3.51 | putative ABC transporter; osmoprotectant-binding protein, glycine betaine:carnitine:choline ABC transporter | *opuCC* | Cell envelope biogenesis, outer membrane |
| sa_c6541s5711_at | SA2431 | 0.00033 | -2.16 | 0.00033 | -4.22 | immunodominant antigen B | *isaB* | Cell envelope biogenesis, outer membrane |
| sa_c6571s5739_a_at | SA2437 | 2.07E-05 | -2.65 | 2.07E-05 | -2.40 | Probable N-acetylmuramidase precursor (Peptidoglycan hydrolase) (Autolysin) (Lysosyme) |  | Cell motility and secretion |
| sa_c3204s2753_a_at | SA1545 | 8.56E-07 | -17.33 | 8.56E-07 | -4.31 | Phosphoglycerate dehydrogenase and related dehydrogenases or D-3-phosphoglycerate dehydrogenase | *serA* | Coenzyme metabolism, Amino acid transport and metabolism |
| sa_c3669s3147_a_at | SA1679 | 3.31E-06 | -2.92 | 3.31E-06 | -2.43 | phosphoglycerate dehydrogenase or related dehydrogenase |  | Coenzyme metabolism, Amino acid transport and metabolism |
| sa_c1246s1026_a_at | SA1070 | 0.000194 | -2.39 | 0.000194 | -2.13 | ATP-DEPENDENT DNA HELICASE RECG (recG) | *recG* | DNA replication, recombination and repair, Transcription |
| sa_c2918s2481_a_at | SA0162 | 0.00954 | -4.07 | 0.00954 | -6.25 | aldehyde dehydrogenase family 1, subfamily A2; retinaldehyde dehydrogenase 2; alcohol dehydrogenase family 1, subfamily A7; alcohol dehydrogenase family 1, subfamily A2; retinaldehyde dehydrogenase | *aldA* | Energy production and conversion |
| sa_c5164s4463_a_at | SA0231 | 0.000568 | -6.37 | 0.000568 | -6.76 | Flavohemoprotein (Hemoglobin-like protein) (Flavohemoglobin) (Dihydropteridine reductase) (Ferrisiderophore reductase B) (Nitric oxide dioxygenase) (NOD) |  | Energy production and conversion |
| sa_c7041s6150_a_at | SA0327 | 1.45E-08 | -8.93 | 1.45E-08 | -5.49 | Alkanal monooxygenase alpha chain |  | Energy production and conversion |
| sa_c7234s6298_a_at | SA0367 | 5.53E-05 | -3.79 | 5.53E-05 | -2.79 | NADPH-flavin oxidoreductase (Flavin reductase P) (NADPH-FMN oxidoreductase) |  | Energy production and conversion |
| sa_c7663s6675_a_at | SA0510 | 0.000211 | -2.39 | 0.000211 | -4.12 | Glycerol kinase (ATP:glycerol 3-phosphotransferase) (Glycerokinase) (GK) | *araB* | Energy production and conversion |
| sa_c8493s7455_a_at | SA0757 | 0.000393 | -2.04 | 0.000393 | -2.86 | putative nitroreductase |  | Energy production and conversion |
| sa_c8671s7618_a_at | SA0817 | 4.63E-06 | -4.93 | 4.63E-06 | -6.10 | PROBABLE NADH-DEPENDENT FLAVIN OXIDOREDUCTASE OXIDOREDUCTASE PROTEIN |  | Energy production and conversion |
| sa_c736s544_a_at | SA0937 | 0.00365 | -2.92 | 0.00365 | -3.51 | Cytochrome D ubiquinol oxidase subunit I (Cytochrome BD-I oxidase subunit I) |  | Energy production and conversion |
| sa_c740s548_a_at | SA0938 | 0.00375 | -2.87 | 0.00375 | -3.38 | cytochrome D ubiquinol oxidase subunit II homolog |  | Energy production and conversion |
| sa_c2847s9197_a_at | SA1451 | 1.89E-06 | -5.85 | 1.89E-06 | -3.55 | Alkanal monooxygenase alpha chain (Bacterial luciferase alpha chain) |  | Energy production and conversion |
| sa_c3194s2739_a_at | SA1542 | 4.80E-06 | -5.32 | 4.80E-06 | -6.80 | Glycerophosphoryl diester phosphodiesterase, periplasmic precursor (Glycerophosphodiester phosphodiesterase) |  | Energy production and conversion |
| sa_c3890s3360_a_at | SA1736 | 5.76E-05 | -4.00 | 5.76E-05 | -3.18 | aldehyde dehydrogenase 3A2; aldehyde dehydrogenase 10; aldehyde dehydrogenase 3 family, member A2; fatty aldehyde dehydrogenase | *aldH* | Energy production and conversion |
| sa_c4999s4299_a_at | SA2075 | 0.000667 | -3.64 | 0.000667 | -2.41 | required for formate dehydrogenase activity narQ | *fdhD* | Energy production and conversion |
| sa_c6062s5256_a_at | SA2311 | 6.07E-06 | -3.16 | 6.07E-06 | -3.95 | NADPH-flavin oxidoreductase |  | Energy production and conversion |
| sa_c6108s9090_a_at | SA2324 | 7.65E-06 | -4.81 | 7.65E-06 | -2.61 | hypothetical protein, similar to thioredoxin |  | Energy production and conversion |
| sa_c9395s8219_a_at | SA2341 | 0.0336 | -2.07 | 0.0336 | -2.40 | aldehyde dehydrogenase family 1, subfamily A2; retinaldehyde dehydrogenase 2; alcohol dehydrogenase family 1, subfamily A7; alcohol dehydrogenase family 1, subfamily A2; retinaldehyde dehydrogenase |  | Energy production and conversion |
| sa_c6220s5396_a_at | SA2346 | 1.07E-05 | -6.76 | 1.07E-05 | -3.37 | D-lactate dehydrogenase (D-LDH) (Fermentative lactate dehydrogenase) |  | Energy production and conversion, Coenzyme metabolism |
| sa_c9413s8229_a_at | SA2395 | 0.00012 | -2.47 | 0.00012 | -2.48 | lactate dehydrogenase 3, C chain; lactate dehydrogenase 3, C chain, sperm specific |  | Energy production and conversion |
| sa_c1758s1494_a_at | SA1186 | 0.000916 | -2.21 | 0.000916 | -2.30 | hypothetical protein predicted by GeneMark |  | Function unknown |
| sa_c2779s2349_a_at | SA1431 | 0.000451 | -2.91 | 0.000451 | -2.14 | MloA |  | Function unknown |
| sa_c3224s2774_a_at | SA0170 | 0.000186 | -2.99 | 0.000186 | -4.02 | hypothetical protein predicted by GeneMark |  | Function unknown |
| sa_c401s238_a_at | SA0862 | 0.000333 | -2.39 | 0.000333 | -2.56 | hypothetical protein predicted by GeneMark |  | Function unknown |
| sa_c4515s3860_a_at | SA1942 | 4.34E-05 | -6.85 | 4.34E-05 | -3.72 | putative membrane protein |  | Function unknown |
| sa_c5102s4400_at | SA0230 | 7.67E-05 | -9.52 | 7.67E-05 | -7.25 | probable uroporphyrin-III c-methyltransferase (EC 2.1.1.107) |  | Function unknown |
| sa_c5492s4755_a_at | SA2164 | 6.72E-07 | -16.98 | 6.72E-07 | -6.62 | hypothetical protein, similar to phage infection protein precursor |  | Function unknown |
| sa_c5729s4971_a_at | SA2224 | 6.16E-05 | -4.42 | 6.16E-05 | -2.28 | hypothetical protein predicted by GeneMark |  | Function unknown |
| sa_c6029s5227_a_at | SA0427 | 4.06E-05 | -2.43 | 4.06E-05 | -2.42 | Uncharacterized conserved membrane protein |  | Function unknown |
| sa_c7276s6334_a_at | SA0380 | 7.34E-05 | -4.10 | 7.34E-05 | -3.13 | conserved hypothetical protein [Pathogenicity island SaPIn2] |  | Function unknown |
| sa_c7400s6425_a_at | SA0428 | 0.000317 | -2.72 | 0.000317 | -2.33 | Uncharacterized conserved membrane protein |  | Function unknown |
| sa_c1934s1655_a_at | SA1230 | 7.86E-09 | -18.73 | 7.86E-09 | -3.08 | IAA-Ala hydrolase; IAA-amino acid hydrolase |  | General function prediction only |
| sa_c2448s2032_a_at | SA1333 | 0.00106 | -2.30 | 0.00106 | -2.80 | 3-oxoacyl-[acyl-carrier protein] reductase (3-ketoacyl-acyl carrier protein reductase) |  | General function prediction only |
| sa_c259s100_a_at | SA2366 | 6.80E-07 | -5.03 | 6.80E-07 | -2.81 | aminocarboxymuconate semialdehyde decarboxylase; 2-amino-3-carboxymuconate-6-semialdehyde decarboxylase |  | General function prediction only |
| sa_c3433s2963_a_at | SA1606 | 1.55E-05 | -5.88 | 1.55E-05 | -7.30 | aldo-keto reductase family 1, member B11 (aldose reductase-like) |  | General function prediction only |
| sa_c4138s3489_a_at | SA1840 | 6.65E-08 | -6.99 | 6.65E-08 | -6.33 | AGR_pAT_287p |  | General function prediction only |
| sa_c4140s3492_a_at | SA1841 | 6.18E-05 | -3.92 | 6.18E-05 | -2.58 | CN_hydrolase, Carbon-nitrogen hydrolase |  | General function prediction only |
| sa_c4687s4008_a_at | SA1989 | 3.38E-05 | -2.46 | 3.38E-05 | -2.46 | Quinone oxidoreductase (NADPH:quinone reductase) (Zeta-crystallin) |  | General function prediction only |
| sa_c4749s4064_a_at | SA2005 | 5.72E-05 | -3.02 | 5.72E-05 | -3.98 | hypothetical protein predicted by GeneMark |  | General function prediction only |
| sa_c6044s5243_a_at | SA2306 | 7.96E-05 | -3.48 | 7.96E-05 | -2.18 | abhydrolase_2, Phospholipase:Carboxylesterase |  | General function prediction only |
| sa_c6058s5252_a_at | SA2310 | 9.74E-08 | -6.41 | 9.74E-08 | -5.62 | predicted ring-cleavage extradiol dioxygenase |  | General function prediction only |
| sa_c6112s5297_a_at | SA2325 | 0.000109 | -5.46 | 0.000109 | -3.07 | 4-hydroxybenzoyl-CoA thioesterase |  | General function prediction only |
| sa_c6186s5364_a_at | SA2339 | 4.08E-06 | -5.95 | 4.08E-06 | -4.42 | PROBABLE TRANSPORT PROTEIN, SIMILAR TO ANTIBIOTIC TRANSPORT-ASSOCIATED PROTEIN ACTII IN STREPTOMYCES COELICOLOR. |  | General function prediction only |
| sa_c6202s5382_a_at | SA2342 | 2.76E-05 | -6.80 | 2.76E-05 | -5.15 | Galactoside acetyltransferase (EC 2.3.1.18) (Thiogalactoside acetyltransferase). |  | General function prediction only |
| sa_c6299s5473_a_at | SA2367 | 1.86E-06 | -4.41 | 1.86E-06 | -2.90 | conserevd hypothetical protein |  | General function prediction only |
| sa_c6353s5521_a_at | SA2380 | 5.38E-05 | -3.30 | 5.38E-05 | -2.90 | glutaryl 7-ACA acylase precursor |  | General function prediction only |
| sa_c6688s5833_a_at | SA2463 | 0.00806 | -2.99 | 0.00806 | -4.72 | Lipase precursor (Triacylglycerol lipase) | *lip* | General function prediction only |
| sa_c7043s6153_a_at | SA0328 | 1.05E-08 | -7.87 | 1.05E-08 | -4.46 | NADH-dependent FMN reductase |  | General function prediction only |
| sa_c7365s6401_a_at | SA0417 | 4.25E-05 | -16.67 | 4.25E-05 | -5.65 | Sodium- and chloride-dependent betaine transporter (Na+:Cl-betaine:GABA transporter) (Sodium- and chloride-dependent GABA transporter 2) (GAT2) |  | General function prediction only |
| sa_c7649s6662_a_at | SA0507 | 2.88E-05 | -4.39 | 2.88E-05 | -2.16 | IAA-Ala hydrolase; IAA-amino acid hydrolase |  | General function prediction only |
| sa_c7679s6686_a_at | SA0513 | 3.83E-06 | -3.98 | 3.83E-06 | -2.29 | HPr(Ser) phosphatase (P-Ser-HPr phosphatase) |  | General function prediction only |
| sa_c8633s7584_a_at | SA0804 | 0.000256 | -10.75 | 0.000256 | -2.15 | putative integral membrane protein |  | General function prediction only |
| sa_c8881s7799_at | SA0558 | 0.000126 | -2.20 | 0.000126 | -2.44 | NADHdh_2, NAD(P)H dehydrogenase (quinone) |  | General function prediction only |
| sa_c8930s7847_a_at | SA0658 | 2.58E-05 | -2.62 | 2.58E-05 | -2.51 | aldo-keto reductase family 1, member B11 (aldose reductase-like) |  | General function prediction only |
| sa_c10102s10518cv_s_at | SA1634 | 3.14E-06 | -2.03 | 3.14E-06 | -2.33 | truncated hypothetical protein [Pathogenicity island SaPIn3] |  | hypothetical protein |
| sa_c10228s8907_a_at | SA2280 | 0.00148 | -2.08 | 0.00148 | -2.63 | Conserved hypothetical ORF |  | hypothetical protein |
| sa_c10316s10702_s_at | SA0129 | 5.14E-05 | -12.45 | 5.14E-05 | -11.21 | Hypothetical ORF |  | hypothetical protein |
| sa_c10359s10751_s_at | SA1332 | 0.00176 | -2.18 | 0.00176 | -2.54 | Conserved hypothetical ORF |  | hypothetical protein |
| sa_c1419s1193_a_at | SA0117 | 0.000111 | -2.54 | 0.000111 | -3.01 | hypothetical protein, similar to rhizobactin siderophore biosynthesisprotein RhsF |  | hypothetical protein |
| sa_c1647s1385_a_at | SA1161 | 8.19E-06 | -2.35 | 8.19E-06 | -2.99 | Conserved hypothetical ORF |  | hypothetical protein |
| sa_c1960s1685_a_at | SA1235 | 7.59E-06 | -4.41 | 7.59E-06 | -2.34 | putative DNA binding protein |  | hypothetical protein |
| sa_c1968s1693_a_at | SA0132 | 0.000372 | -3.12 | 0.000372 | -4.20 | multidrug resistance protein (efflux transporter) |  | hypothetical protein |
| sa_c2432s2017_a_at | SA1327 | 0.000738 | -3.75 | 0.000738 | -2.51 | Hypothetical protein ypuF (ORFX6) |  | hypothetical protein |
| sa_c2942s2505_a_at | SA1476 | 1.23E-07 | -6.54 | 1.23E-07 | -10.10 | Conserved hypothetical ORF |  | hypothetical protein |
| sa_c3244s2793_a_at | SA1555 | 0.000158 | -7.58 | 0.000158 | -8.77 | cetoin dehydrogenase acuA |  | hypothetical protein |
| sa_c395s234_a_at | SA0861 | 7.95E-06 | -2.72 | 7.95E-06 | -2.53 | Bac_globin, Protozoan:cyanobacterial globin |  | hypothetical protein |
| sa_c403s242_a_at | SA0863 | 0.00718 | -2.07 | 0.00718 | -2.00 | hypothetical protein predicted by GeneMark |  | hypothetical protein |
| sa_c4401s3753_at | SA1900 | 0.0014 | -2.12 | 0.0014 | -2.85 | Conserved hypothetical ORF |  | hypothetical protein |
| sa_c4661s3981_a_at | SA1982 | 0.000247 | -2.89 | 0.000247 | -4.31 | ABC transporter membrane-spanning permease - macrolide efflux |  | hypothetical protein |
| sa_c5170s4467_a_at | SA2118 | 0.00431 | -2.77 | 0.00431 | -2.37 | Conserved hypothetical ORF |  | hypothetical protein |
| sa_c5208s4508_a_at | SA2128 | 0.00115 | -2.62 | 0.00115 | -2.50 | hypothetical protein predicted by GeneMark [Bacillus anthracis A2012] match=17.87% identical |  | hypothetical protein |
| sa_c5303s4583_a_at | SA2149 | 1.28E-07 | -35.84 | 1.28E-07 | -64.94 | ABC TRANSPORTER ATP-BINDING PROTEIN : ABC TRANSPORTER PERMEASE PROTEIN |  | hypothetical protein |
| sa_c5307s4587_at | SA2150 | 9.33E-07 | -36.50 | 9.33E-07 | -68.97 | ABC-type transporter, permease component |  | hypothetical protein |
| sa_c542s370_a_at | SA0893 | 0.00498 | -2.83 | 0.00498 | -3.34 | acatyltransferases homolog |  | hypothetical protein |
| sa_c5460s4726_a_at | SA2159 | 0.000435 | -3.82 | 0.000435 | -4.00 | hypothetical protein, simialr to transcription repressor of sporulation, septation and degradation paiA |  | hypothetical protein |
| sa_c5592s4849_a_at | SA2190 | 0.000384 | -3.16 | 0.000384 | -2.35 | conserved hypothetcial protein |  | hypothetical protein |
| sa_c5954s5161_a_at | SA0203 | 0.000529 | -2.18 | 0.000529 | -3.57 | hypothetical protein [Pathogenicity island SaPIn2] |  | hypothetical protein |
| sa_c6005s5207_a_at | SA2297 | 2.14E-05 | -2.01 | 2.14E-05 | -4.50 | weakly similar to GTP-pyrophosphokinase |  | hypothetical protein |
| sa_c6182s5363_at | SA2338 | 0.00195 | -3.65 | 0.00195 | -2.13 | hypothetical protein predicted by GeneMark |  | hypothetical protein |
| sa_c6206s5387_a_at | SA2343 | 6.25E-06 | -21.65 | 6.25E-06 | -23.64 | Conserved hypothetical ORF |  | hypothetical protein |
| sa_c629s448_at | SA0914 | 3.83E-07 | -4.37 | 3.83E-07 | -6.37 | hypothetical protein, similar to chitinase B |  | hypothetical protein |
| sa_c6595s5763_a_at | SA2444 | 1.87E-05 | -2.04 | 1.87E-05 | -3.85 | Conserved hypothetical ORF |  | hypothetical protein |
| sa_c6758s5899_a_at | SA2479 | 6.06E-08 | -12.17 | 6.06E-08 | -12.89 | putative exported protein |  | hypothetical protein |
| sa_c7255s6315_a_at | SA0372 | 0.00113 | -2.38 | 0.00113 | -2.38 | Conserved hypothetical ORF |  | hypothetical protein |
| sa_c7313s9398_a_at | SA0395 | 0.00291 | -2.29 | 0.00291 | -2.37 | hypothetical protein [Pathogenicity island SaPIn2] |  | hypothetical protein |
| sa_c813s612_a_at | SA0955 | 0.000335 | -3.03 | 0.000335 | -2.27 | Conserved hypothetical ORF |  | hypothetical protein |
| sa_c820s622_a_at | SA0957 | 0.00674 | -2.51 | 0.00674 | -2.26 | hypothetical protein predicted by GeneMark |  | hypothetical protein |
| sa_c8491s7451_a_at | SA0756 | 0.000229 | -2.39 | 0.000229 | -2.12 | 3-dehydroquinate dehydratase (3-dehydroquinase) (Type I DHQase) |  | hypothetical protein |
| sa_c8828s7763_a_at | SA1599 | 4.10E-05 | -2.34 | 4.10E-05 | -2.07 | Transaldolase, Transaldolase |  | hypothetical protein |
| sa_c8897s7814_a_at | SA0591 | 5.24E-05 | -3.13 | 5.24E-05 | -3.13 | Conserved hypothetical ORF |  | hypothetical protein |
| sa_c9112s7982_a_at | SA0857 | 2.59E-06 | -2.61 | 0.000126 | -2.11 | NADP-SPECIFIC GLUTAMATE DEHYDROGENASE (NADP-GDH) (NAD(P)H-DEPENDENT GLUTAMATE DEHYDROGENASE) |  | hypothetical protein |
| sa_c9330s9270_a_at | SA2283 | 0.000124 | -3.51 | 0.000124 | -2.58 | hypothetical protein predicted by GeneMark |  | hypothetical protein |
| sa_i2983ur_x_at | SA1613 | 0.00182 | -2.54 | 0.00182 | -2.22 | reverse complement of intergenic upstream of ORF sa_c3455s2983 |  | hypothetical protein |
| sa_c4572s3904_a_at | SA1958 | 6.42E-06 | -3.06 | 0.00175 | -2.84 | PTS system, mannitol-specific IIBC component (EIIBC-Mtl) (Mannitol-permease IIBC component) (Phosphotransferase enzyme II, BC component) (EII-Mtl) |  | Inorganic ion transport and metabolism |
| sa_c815s618cs_s_at | SA0956 | 4.24E-05 | -3.50 | 4.24E-05 | -2.34 | solute carrier family 11 member 2; Natural resistance-associated macrophage protein 2; Solute carrier family 11 member 2 (natural resistance-associated macrophage protein 2) |  | Inorganic ion transport and metabolism |
| sa_c9528s8308_a_at | SA0122 | 1.13E-06 | -5.13 | 1.13E-06 | -2.92 | 3-oxoacyl-[acyl-carrier protein] reductase (3-ketoacyl-acyl carrier protein reductase) | *butA* | Lipid metabolism, Secondary metabolites biosynthesis, transport and catabolism |
| sa_c4097s3454_a_at | SA0204 | 2.11E-07 | -8.93 | 2.11E-07 | -11.01 | Putative acyl carrier protein phosphodiesterase (ACP phosphodiesterase) | *acpD* | Lipid metabolism |
| sa_c4820s4127_a_at | SA0224 | 0.00147 | -3.40 | 0.00147 | -9.71 | Fatty oxidation complex alpha subunit; Enoyl-CoA hydratase; Delta(3)-cis-delta(2)-trans-enoyl-CoA isomerase; 3-hydroxyacyl-CoA dehydrogenase; 3-hydroxybutyryl-CoA epimerase |  | Lipid metabolism |
| sa_c4884s4191_a_at | SA0226 | 2.54E-05 | -9.71 | 2.54E-05 | -13.70 | long-chain fatty-acid-CoA ligase; acid-CoA ligase |  | Lipid metabolism, Secondary metabolites biosynthesis, transport and catabolism |
| sa_c4917s4223_a_at | SA0227 | 1.05E-05 | -8.47 | 1.05E-05 | -7.94 | 3-oxoacid CoA transferase precursor; Succinyl CoA:3-oxoacid CoA transferase; succinyl-CoA:3-ketoacid-CoA transferase precursor |  | Lipid metabolism |
| sa_c3241s2789_a_at | SA1554 | 0.00358 | -5.26 | 0.00358 | -5.78 | acetyl-CoA synthetase isoform b; cytoplasmic acetyl-coenzyme A synthetase; acetate-CoA ligase; acyl-activating enzyme; acetate thiokinase; acetyl-CoA synthetase | *acsA* | Lipid metabolism |
| sa_c6106s5295_a_at | SA2323 | 8.60E-06 | -3.31 | 8.60E-06 | -2.16 | putative esterase:acetyl hydrolase |  | Lipid metabolism |
| sa_c6293s5471_a_at | SA2365 | 1.43E-06 | -8.00 | 1.43E-06 | -3.21 | similar to androgen-regulated short-chain dehydrogenase:reductase 1 |  | Lipid metabolism, Secondary metabolites biosynthesis, transport and catabolism |
| sa_c6401s5571_a_at | SA2402 | 9.67E-07 | -3.19 | 9.67E-07 | -2.60 | Acetyl-coenzyme A synthetase (Acetate--CoA ligase) (Acyl-activating enzyme) (Acetyl-CoA synthase) |  | Lipid metabolism |
| sa_c1898s1619_a_at | SA0131 | 0.000325 | -4.39 | 0.000325 | -7.09 | Purine nucleoside phosphorylase (Inosine phosphorylase) (PNP) | *pnp* | Nucleotide transport and metabolism |
| sa_c6441s5608_a_at | SA2410 | 0.000325 | -4.05 | 0.000325 | -2.39 | anaerobic ribonucleoside-triphosphate reductase | *nrdD* | Nucleotide transport and metabolism |
| sa_c986s774_a_at | SA0998 | 0.000101 | -2.76 | 0.000101 | -2.04 | HAM1 protein homolog; Ham1 |  | Nucleotide transport and metabolism |
| sa_c3196s2743_a_at | SA1543 | 5.04E-05 | -3.91 | 5.04E-05 | -2.74 | organic hydroperoxide resistance protein |  | Posttranslational modification, protein turnover, chaperones |
| sa_c3220s2767_at | SA1549 | 5.65E-06 | -2.03 | 5.65E-06 | -4.63 | periplasmic serine protease Do; heat shock protein HtrA |  | Posttranslational modification, protein turnover, chaperones |
| sa_c3603s3083_a_at | SA1659 | 7.54E-06 | -2.39 | 7.54E-06 | -3.46 | Parvulin-like PPIase precursor (Peptidyl-prolyl cis-trans isomerase plp) (Rotamase plp) | *prsA* | Posttranslational modification, protein turnover, chaperones |
| sa_c3671s3151_a_at | SA1680 | 3.72E-05 | -2.89 | 3.72E-05 | -2.34 | thioredoxin dependent peroxide reductase 1 |  | Posttranslational modification, protein turnover, chaperones |
| sa_c5035s4334_at | SA2085 | 1.71E-05 | -2.88 | 1.71E-05 | -4.18 | urease accessory protein UreE | *ureE* | Posttranslational modification, protein turnover, chaperones |
| sa_c5039s4340_a_at | SA2086 | 1.40E-05 | -2.33 | 1.40E-05 | -3.79 | urease accessory protein UreF | *ureF* | Posttranslational modification, protein turnover, chaperones |
| sa_c9293s8136_a_at | SA2088 | 0.000118 | -2.19 | 0.000118 | -2.78 | urease accessory protein UreD | *ureD* | Posttranslational modification, protein turnover, chaperones |
| sa_c5472s9152_a_at | SA2162 | 0.000211 | -2.15 | 0.000211 | -3.22 | Thioredoxin reductase 1 (NADPH-dependent thioredoxin reductase 1) (NTR 1) |  | Posttranslational modification, protein turnover, chaperones |
| sa_c5530s4787_a_at | SA2175 | 0.00117 | -2.17 | 0.00117 | -2.07 | WHEAT 16.9 KD CLASS I HEAT SHOCK PROTEIN (LOW MOLECULAR WEIGHT HEAT SHOCK PROTEIN) (HEAT SHOCK PROTEIN 17) (HSP 16.9) |  | Posttranslational modification, protein turnover, chaperones |
| sa_c6173s5353_a_at | SA2336 | 1.51E-05 | -4.08 | 1.51E-05 | -6.13 | ATP-dependent protease ATP-binding subunit (fragment); putative ATP-dependent protease ATP-binding subunit | *clpL* | Posttranslational modification, protein turnover, chaperones |
| sa_c6435s5604_a_at | SA2409 | 0.000134 | -4.61 | 0.000134 | -2.22 | Anaerobic ribonucleoside-triphosphate reductase activating protein (Class III anaerobic ribonucleotide reductase small component) |  | Posttranslational modification, protein turnover, chaperones |
| sa_c6467s5637_a_at | SA2414 | 9.52E-05 | -2.04 | 9.52E-05 | -2.33 | glutathione peroxidase 4; sperm nuclei glutathione peroxidase; phospholipid hydroperoxide glutathione peroxidase; 1700027O09Rik |  | Posttranslational modification, protein turnover, chaperones |
| sa_c372s215_a_at | SA2490 | 0.000252 | -4.93 | 0.000252 | -4.12 | Arylamine N-acetyltransferase 2 (Arylamide acetylase 2) (Arylamine N-acetyltransferase, polymorphic) (PNAT) (N-acetyltransferase type 2) (NAT-2) |  | Secondary metabolites biosynthesis, transport and catabolism |
| sa_c3341s2876_a_at | SA0173 | 4.57E-05 | -2.39 | 4.57E-05 | -2.27 | Delta-(L-alpha-aminoadipyl)-L-cysteinyl-D-valine synthetase (ACV synthetase) (ACVS) |  | Secondary metabolites biosynthesis, transport and catabolism |
| sa_c392s9574cs_s_at | SA0860 | 0.000217 | -2.72 | 0.000217 | -2.64 | GTP pyrophosphokinase |  | Secondary metabolites biosynthesis, transport and catabolism |
| sa_c4301s3654_a_at | SA1882 | 0.000912 | -3.37 | 0.000912 | -2.83 | PROBABLE TWO-COMPONENT SENSOR KINASE KDPD TRANSCRIPTION REGULATOR PROTEIN | *kdpD* | Signal transduction mechanisms |
| sa_c8744s7687_a_at | SA1532 | 1.48E-05 | -2.66 | 1.48E-05 | -2.70 | Hypothetical 14.6 kDa protein in QAH:OAS sulfhydrylase 3region |  | Signal transduction mechanisms |
| sa_c6963s6087_a_at | SA0305 | 0.00641 | -2.62 | 0.00641 | -4.08 | ROK family-glucose kinase or transcriptional regulator |  | Transcription, Carbohydrate transport and metabolism |
| sa_c6967s6091_a_at | SA0306 | 0.000104 | -5.95 | 0.000104 | -2.73 | transcriptional regulator (hex regulon repressor) |  | Transcription |
| sa_c7405s6429_a_at | SA0429 | 0.0142 | -2.87 | 0.0142 | -2.06 | transcriptional activator of the glutamate synthase operon (LysR family) | *gltC* | Transcription |
| sa_c8791s7731_a_at | SA0476 | 0.000198 | -4.65 | 0.000198 | -4.07 | similar to transcriptional regulator (GntR family) : aminotransferase (MocR-like) |  | Transcription, Amino acid transport and metabolism |
| sa_c1781s1514_at | SA1191 | 8.18E-05 | -2.54 | 8.18E-05 | -2.18 | transcription antiterminator (BglG family) licT | *glcT* | Transcription |
| sa_c3250s2795_a_at | SA1556 | 0.000561 | -4.95 | 0.000561 | -6.54 | histone deacetylase 9 isoform 1; histone deacetylase 7B; histone deacetylase 7; MEF-2 interacting transcription repressor (MITR) protein | *acuC* | Transcription |
| sa_c4741s4054_a_at | SA2002 | 7.89E-06 | -4.07 | 7.89E-06 | -2.50 | Transcriptional regulator cueR (Copper efflux regulator) (Copper export regulator) |  | Transcription |
| sa_c5496s4759_a_at | SA2165 | 8.19E-06 | -2.82 | 8.19E-06 | -2.13 | TetR family HTH transcriptional regulator |  | Transcription |
| sa_c6050s5246_a_at | SA2308 | 0.000407 | -5.68 | 0.000407 | -3.31 | transcriptional regulator (MarR family) |  | Transcription |
| sa_c6190s5368_a_at | SA2340 | 2.70E-08 | -5.49 | 2.70E-08 | -2.02 | transcriptional regulator, TetR family, putative |  | Transcription |
| sa_c441s275_a_at | SA2364 | 3.54E-07 | -8.06 | 3.54E-07 | -3.41 | transcription regulator, TetR:AcrR family |  | Transcription |
| sa_c6673s5826_a_at | SA2458 | 1.17E-05 | -3.42 | 1.17E-05 | -4.78 | ica operon transcription regulator IcaR | *icaR* | Transcription |
| sa_c7051s6163_a_at | SA0330 | 2.81E-06 | -6.80 | 2.81E-06 | -2.87 | Ribosomal-protein-serine acetyltransferase (EC 2.3.1.-) (Acetylating enzyme for n-terminal of ribosomal protein L7:L12). |  | Translation, ribosomal structure and biogenesis |
| sa_c1691s1429_at | SA1171 | 0.000179 | -2.11 | 0.000179 | -2.29 | 30S ribosomal protein S14; ribosomal protein S14 | *rpsN* | Translation, ribosomal structure and biogenesis |
| sa_c8748s7692_a_at | SA1550 | 5.13E-06 | -3.08 | 5.13E-06 | -2.53 | Tyrosyl-tRNA synthetase (Tyrosine--tRNA ligase) (TyrRS) | *tyrS* | Translation, ribosomal structure and biogenesis |
|  |  |  |  |  |  |  |  |  |
| **Group V: Downregulation (20min) - No change (60 min) 158 genes** | | | | | | |  |  |
| sa_c37s34_a_at | SA0010 | 2.44E-06 | -4.67 |  |  | branched-chain amino acid permease |  | Amino acid transport and metabolism |
| sa_c4055s3432_a_at | SA0201 | 0.00111 | -2.33 |  |  | RGD-containing lipoprotein | *rlp* | Amino acid transport and metabolism |
| sa_c5694s4942_a_at | SA0239 | 0.000141 | -2.05 |  |  | Sorbitol dehydrogenase (L-iditol 2-dehydrogenase) (Glucitol dehydrogenase) |  | Amino acid transport and metabolism |
| sa_c5789s5030_a_at | SA0240 | 0.000114 | -2.13 |  |  | Sorbitol dehydrogenase (L-iditol 2-dehydrogenase) (Glucitol dehydrogenase) |  | Amino acid transport and metabolism |
| sa_c5865s5108_a_at | SA0242 | 0.000113 | -2.07 |  |  | Sorbitol dehydrogenase (L-iditol 2-dehydrogenase) (Glucitol dehydrogenase) |  | Amino acid transport and metabolism |
| sa_c6930s6051_a_at | SA0294 | 0.000593 | -2.34 |  |  | Branched-chain amino acid transport system carrier protein brnQ (Branched-chain amino acid uptake carrier brnQ) |  | Amino acid transport and metabolism |
| sa_c7100s6210_a_at | SA0344 | 2.80E-06 | -15.15 |  |  | methyltetrahydropteroyltriglutamate--homocysteine methyltransferase (Vitamin-B12-independent methionine synthase isozyme) (Cobalamin-independent methionine synthase isozyme) | *metE* | Amino acid transport and metabolism |
| sa_c7102s6213_a_at | SA0345 | 5.38E-06 | -13.64 |  |  | 5-methyltetrahydrofolate-homocysteine methyltransferase; 5-methyltetrahydrofolate-homocysteine methyltransferase 1 |  | Amino acid transport and metabolism |
| sa_c7106s6219_a_at | SA0346 | 5.10E-06 | -11.83 |  |  | cystathionine gamma-synthase (O-succinylhomoserine (thiol)-lyase) metB |  | Amino acid transport and metabolism |
| sa_c7112s6224_a_at | SA0347 | 2.45E-06 | -9.35 |  |  | cystathionine gamma-synthase (O-succinylhomoserine (thiol)-lyase) metB |  | Amino acid transport and metabolism |
| sa_c7410s6434_a_at | SA0430 | 0.000217 | -2.58 |  |  | Glutamate synthase [NADPH] large chain precursor (Glutamate synthase alpha subunit) (NADPH-GOGAT) (GLTS alpha chain) | *gltB* | Amino acid transport and metabolism |
| sa_c7412s6438_a_at | SA0431 | 0.00033 | -3.00 |  |  | Glutamate synthase [NADPH] small chain (Glutamate synthase beta subunit) (NADPH-GOGAT) (GLTS beta chain) | *gltD* | Amino acid transport and metabolism |
| sa_c5343s4616_a_at | SA0677 | 0.00115 | -2.27 |  |  | putative ABC transporter, ATP-binding protein, proline:glycine betaine transport system |  | Amino acid transport and metabolism |
| sa_c8240s7220_a_at | SA0679 | 3.62E-06 | -5.75 |  |  | histidinol-phosphate aminotransferase; histidinol-phosphate aminotransferase:tyrosine and phenylalanine aminotransferase |  | Amino acid transport and metabolism |
| sa_c350s191_a_at | SA0849 | 8.12E-05 | -2.11 |  |  | oligopeptide ABC transporter, periplasmic oligopeptide-binding protein (oppA-2) homolog |  | Amino acid transport and metabolism |
| sa_c352s195_a_at | SA0850 | 0.000757 | -3.42 |  |  | periplasmic oligopeptide-binding protein of oligopeptide ABC transporter |  | Amino acid transport and metabolism |
| sa_c434s266_a_at | SA0871 | 6.99E-07 | -7.19 |  |  | amino acid carrier protein (sodium:alanine symporter) |  | Amino acid transport and metabolism |
| sa_c1810s1538_a_at | SA1197 | 0.000524 | -2.31 |  |  | AROGENATE DEHYDROGENASE : PREPHENATE DEHYDROGENASE | *tyrA* | Amino acid transport and metabolism |
| sa_c1820s1547_a_at | SA1200 | 0.00698 | -2.09 |  |  | para-aminobenzoate synthetase glutamine amidotransferase component II:anthranilate synthase component II; para-aminobenzoate:anthranilate synthase glutamine amidotransferase component II |  | Amino acid transport and metabolism, Coenzyme metabolism |
| sa_c1828s1551_a_at | SA1201 | 0.000803 | -2.82 |  |  | pir|AE0653 anthranilate synthase component II, anthranilate phosphoribosyltransferase | *trpD* | Amino acid transport and metabolism |
| sa_c1832s1558_a_at | SA1202 | 0.00239 | -4.46 |  |  | anthranilate synthase; indole-glycerol phosphate synthase; phosphoribosyl anthranilate isomerase | *trpC* | Amino acid transport and metabolism |
| sa_c1836s1562_at | SA1203 | 0.00279 | -4.27 |  |  | N-(5-phosphoribosyl)anthranilate isomerase and indole-3-glycerolphosphate synthetase | *trpF* | Amino acid transport and metabolism |
| sa_c1840s1566_a_at | SA1204 | 0.000663 | -3.64 |  |  | tryptophan synthase beta chain; tryptophan synthase (beta subunit) | *trpB* | Amino acid transport and metabolism |
| sa_c1844s1570_a_at | SA1205 | 5.52E-05 | -2.63 |  |  | tryptophan synthase alpha chain; tryptophan synthase (alpha subunit) | *trpA* | Amino acid transport and metabolism |
| sa_c1866s1587_a_at | SA1211 | 0.000158 | -2.30 |  |  | PROBABLE ABC TRANSPORTER ATP BINDING ABC TRANSPORTER PROTEIN | *opp-2F* | Amino acid transport and metabolism |
| sa_c9702s8459_a_at | SA1334 | 0.00411 | -2.02 |  |  | pyrroline-5-carboxylate reductase 1 isoform 2; P5C reductase |  | Amino acid transport and metabolism |
| sa_c2466s2048_a_at | SA1340 | 0.016 | -2.10 |  |  | Conserved hypothetical ORF |  | Amino acid transport and metabolism |
| sa_c4209s3561_a_at | SA1858 | 0.000144 | -6.17 |  |  | Dihydroxy-acid dehydratase (DAD) | *ilvD* | Amino acid transport and metabolism, Coenzyme metabolism |
| sa_c4213s3565_a_at | SA1859 | 0.000431 | -8.00 |  |  | Acetolactate synthase isozyme III large subunit (AHAS-III) (Acetohydroxy-acid synthase III large subunit) (ALS-III) | *ilvB* | Amino acid transport and metabolism, Coenzyme metabolism |
| sa_c4217s3569_at | SA1860 | 0.000487 | -11.82 |  |  | Acetolactate synthase small subunit (AHAS) (Acetohydroxy-acid synthase small subunit) (ALS) |  | Amino acid transport and metabolism |
| sa_c9931s8627_a_at | SA1861 | 0.000255 | -10.31 |  |  | Ketol-acid reductoisomerase (Acetohydroxy-acid isomeroreductase) (Alpha-keto-beta-hydroxylacil reductoisomerase) | *ilvC* | Amino acid transport and metabolism, Coenzyme metabolism |
| sa_c4223s3575_a_at | SA1862 | 4.06E-05 | -9.80 |  |  | 2-isopropylmalate synthase (Alpha-isopropylmalate synthase) (Alpha-IPM synthetase) [] | *leuA* | Amino acid transport and metabolism |
| sa_c4229s3580_a_at | SA1864 | 1.39E-05 | -13.18 |  |  | 3-isopropylmalate dehydratase large subunit (Isopropylmalate isomerase) (Alpha-IPM isomerase) (IPMI) | *leuC* | Amino acid transport and metabolism |
| sa_c4239s3588_a_at | SA1865 | 6.09E-05 | -13.05 |  |  | 3-isopropylmalate dehydratase small subunit (Isopropylmalate isomerase) (Alpha-IPM isomerase) (IPMI) | *leuD* | Amino acid transport and metabolism |
| sa_c4243s3594_a_at | SA1866 | 1.58E-05 | -7.25 |  |  | Threonine dehydratase biosynthetic (Threonine deaminase) (TD) | *ilvA* | Amino acid transport and metabolism |
| sa_c5795s5035_a_at | SA2239 | 3.97E-05 | -3.32 |  |  | solute carrier family 7 (cationic amino acid, transporter, y+ system), member 11 |  | Amino acid transport and metabolism |
| sa_c5817s5059_a_at | SA2248 | 0.000117 | -2.51 |  |  | Glutamate synthase [NADPH] large chain precursor (Glutamate synthase alpha subunit) (NADPH-GOGAT) (GLTS alpha chain) |  | Amino acid transport and metabolism |
| sa_c6378s5547_a_at | SA2396 | 3.90E-05 | -5.18 |  |  | solute carrier family 7 (cationic amino acid transporter, y+ system), member 1 |  | Amino acid transport and metabolism |
| sa_c6385s5555_a_at | SA2397 | 2.20E-06 | -11.86 |  |  | Diaminobutyrate--2-oxoglutarate aminotransferase (L-diaminobutyric acid transaminase) (Diaminobutyrate transaminase) (DABA aminotransferase) (DABA-AT) (L-2,4-diaminobutyrate:2-ketoglutarate 4-aminotransferase) |  | Amino acid transport and metabolism |
| sa_c9447s10370cs_s_at | SA2464 | 0.00225 | -10.50 |  |  | histidine biosynthesis bifunctional protein(phosphoribosyl-AMP cyclohydrolase:phosphoribosyl-ATP pyrophosphohydrolase); histidine biosynthesis bifunctional protein | *hisI* | Amino acid transport and metabolism |
| sa_c6696s10090cs_s_at | SA2465 | 0.00199 | -10.55 |  |  | Imidazole glycerol phosphate synthase subunit hisF (IGP synthase cyclase subunit) (IGP synthase subunit hisF) (ImGP synthase subunit hisF) (IGPS subunit hisF) | *hisF* | Amino acid transport and metabolism |
| sa_c6706s5846_a_at | SA2466 | 0.00148 | -14.45 |  |  | (5-phosphoribosyl)-5-[(5-phosphoribosylamino)methylideneamino] imidazole-4-carboxamide isomerase (Phosphoribosylformimino-5-aminoimidazole carboxamide ribotide isomerase) |  | Amino acid transport and metabolism |
| sa_c6708s5850_a_at | SA2467 | 0.00213 | -14.64 |  |  | Imidazole glycerol phosphate synthase subunit hisH (IGP synthase glutamine amidotransferase subunit) (IGP synthase subunit hisH) (ImGP synthase subunit hisH) (IGPS subunit hisH) | *hisH* | Amino acid transport and metabolism |
| sa_c6714s5853_a_at | SA2468 | 0.00639 | -13.55 |  |  | Imidazoleglycerol-phosphate dehydratase (IGPD) | *hisB* | Amino acid transport and metabolism |
| sa_c6718s5857_a_at | SA2469 | 0.00106 | -19.84 |  |  | histidinol-phosphate aminotransferase; histidinol-phosphate aminotransferase:tyrosine and phenylalanine aminotransferase |  | Amino acid transport and metabolism |
| sa_c6728s5871_a_at | SA2472 | 0.0059 | -14.45 |  |  | hypothetical protein, similar to ATP phosphoribosyltransferase regulatory subunit | *hisZ* | Amino acid transport and metabolism |
| sa_c5110s4406_a_at | SA2104 | 8.38E-05 | -2.11 |  |  | Myo-inositol-1(or 4)-monophosphatase (IMPase) (IMP) (Inositol monophosphatase) (Lithium-sensitive myo-inositol monophosphatase A1) |  | Carbohydrate transport and metabolism |
| sa_c9698s8456_a_at | SA1336 | 5.55E-06 | -2.11 |  |  | glucose-6-phosphate dehydrogenase 2; glucose-6-phosphate dehydrogenase X-linked, pseudogene 1 |  | Carbohydrate transport and metabolism |
| sa_c2030s1741_a_at | SA1255 | 1.69E-05 | -2.04 |  |  | PTS system, glucose-specific IIA component (EIIA-GLC) (Glucose-permease IIA component) (Phosphotransferase enzyme II, A component) (EIII-GLC) |  | Carbohydrate transport and metabolism |
| sa_c5893s5128_a_at | SA0243 | 0.000756 | -2.26 |  |  | hypothetical protein, similar to teichoic acid biosynthesis protein B |  | Cell envelope biogenesis, outer membrane |
| sa_c6285s5463_a_at | SA2362 | 0.000596 | -3.27 |  |  | HSCARG protein |  | Cell envelope biogenesis, outer membrane, Carbohydrate transport and metabolism |
| sa_c9442s8255_a_at | SA2459 | 0.0016 | -2.50 |  |  | intercellular adhesion protein IcaA | *icaA* | Cell envelope biogenesis, outer membrane |
| sa_c6677s5830_a_at | SA2460 | 0.0136 | -2.00 |  |  | IcaD | *icaD* | Cell envelope biogenesis, outer membrane |
| sa_c6681s9106_a_at | SA2461 | 3.83E-06 | -2.07 |  |  | intercellular adhesion protein IcaB | *icaB* | Cell envelope biogenesis, outer membrane |
| sa_c5875s5111_a_at | SA2261 | 4.32E-05 | -3.34 |  |  | Putative efflux pump component MtrF |  | Coenzyme metabolism |
| sa_c3784s3254_a_at | SA1711 | 0.000599 | -2.58 |  |  | polymerase (DNA directed), kappa; DinB homolog 1 (E. coli); DNA damage-inducible proetin b; DNA damage-inducible protein b; polymerase (DNA directed) kappa |  | DNA replication, recombination and repair |
| sa_c6167s5349_a_at | SA2335 | 0.000226 | -2.40 |  |  | Methylated-DNA--protein-cysteine methyltransferase (6-O-methylguanine-DNA methyltransferase) (O-6-methylguanine-DNA-alkyltransferase) |  | DNA replication, recombination and repair |
| sa_c3774s3246_a_at | SA1710 | 0.00156 | -2.09 |  |  | DNA-directed DNA polymerase (EC 2.7.7.7) III epsilon chain |  | DNA replication, recombination and repair |
| sa_c2154s1855_a_at | SA1286 | 8.86E-05 | -2.08 |  |  | hypothetical protein, similar to chromosome replication initiation protein dnaD |  | DNA replication, recombination and repair |
| sa_c8705s9226_a_at | SA0827 | 5.57E-05 | -2.08 |  |  | ATP-dependent deoxyribonuclease (chain B) homolog addB |  | DNA replication, recombination and repair |
| sa_c2146s1847_at | SA1285 | 0.000639 | -2.07 |  |  | nth endonuclease III-like 1 (E. coli); nth (E.coli endonuclease III)-like 1 | *nth* | DNA replication, recombination and repair |
| sa_c853s654_a_at | SA0963 | 0.0024 | -2.18 |  |  | acetyl-CoA carboxylase alpha chain : propionyl-CoA carboxylase alpha chain | *pycA* | Energy production and conversion |
| sa_c4225s3576_a_at | SA1863 | 5.85E-05 | -11.03 |  |  | 3-isopropylmalate dehydrogenase (Beta-IPM dehydrogenase) (IMDH) (3-IPM-DH) | *leuB* | Energy production and conversion, Amino acid transport and metabolism |
| sa_c503s329_a_at | SA0886 | 0.000146 | -4.98 |  |  | hypothetical protein, similar to lactococcin 972 immunity factor |  | Function unknown |
| sa_c5299s4579_a_at | SA2148 | 0.000105 | -4.27 |  |  | putative membrane protein |  | Function unknown |
| sa_c47s43_a_at | SA0011 | 4.27E-07 | -4.26 |  |  | Probable homoserine O-acetyltransferase (Homoserine O-trans-acetylase) |  | Function unknown |
| sa_c6289s5467_a_at | SA2363 | 2.43E-05 | -4.26 |  |  | Conserved hypothetical ORF |  | Function unknown |
| sa_c8244s7224_a_at | SA0680 | 6.39E-05 | -3.98 |  |  | 5(3)-deoxyribonucleotidase |  | Function unknown |
| sa_c9378s8205_a_at | SA2301 | 0.00101 | -2.66 |  |  | alkaline phosphatase-like protein |  | Function unknown |
| sa_c8320s7299_a_at | SA0703 | 0.000252 | -2.29 |  |  | proline dipeptidase (pepQ) |  | Function unknown |
| sa_c10323s10711_s_at | SA1020 | 0.000462 | -2.20 |  |  | Hypothetical ORF |  | Function unknown |
| sa_c10305s8989_a_at | SA0864 | 0.000105 | -2.17 |  |  | GTP pyrophosphokinase (ATP:GTP 3-pyrophosphotransferase) (ppGpp synthetase I) ((p)ppGpp synthetase) |  | Function unknown |
| sa_c1201s981_a_at | SA1057 | 0.0213 | -2.11 |  |  | Predicted membrane protein |  | Function unknown |
| sa_c10641s11095_s_at | SA0968 | 0.000102 | -2.07 |  |  | putative vacuolating cytotoxin paralog |  | Function unknown |
| sa_c5148s4444_a_at | SA2112 | 3.05E-07 | -13.25 |  |  | SODIUM:TAUROCHOLATE COTRANSPORTING POLYPEPTIDE, ILEAL |  | General function prediction only |
| sa_c7094s6206_a_at | SA0343 | 3.94E-05 | -4.72 |  |  | hypothetical protein predicted by GeneMark |  | General function prediction only |
| sa_c6262s5443_a_at | SA2357 | 1.29E-05 | -4.29 |  |  | hypothetical protein, similar to regulatory protein (pfoS:R) |  | General function prediction only |
| sa_c3210s2757_a_at | SA1546 | 7.15E-06 | -3.86 |  |  | Conserved hypothetical ORF |  | General function prediction only |
| sa_c7070s6181_a_at | SA0336 | 0.000346 | -3.75 |  |  | Conserved hypothetical ORF |  | General function prediction only |
| sa_c5142s4440_at | SA2111 | 3.50E-05 | -3.66 |  |  | HPr(Ser) phosphatase (P-Ser-HPr phosphatase) |  | General function prediction only |
| sa_c6783s5921_a_at | SA2487 | 0.000188 | -3.48 |  |  | chloramphenicol-sensitive protein RarD |  | General function prediction only |
| sa_c10529s9046_a_at | SA2361 | 0.00035 | -3.24 |  |  | predicted lactoylglutathione lyase |  | General function prediction only |
| sa_c6027s5223_a_at | SA0753 | 0.000387 | -3.13 |  |  | LysE family transporter lysE |  | General function prediction only |
| sa_c5899s5133_a_at | SA2266 | 4.75E-05 | -3.03 |  |  | 3-oxoacyl-[acyl-carrier protein] reductase (EC 1.1.1.100) (3-ketoacyl- acyl carrier protein reductase). |  | General function prediction only |
| sa_c990s778_a_at | SA0999 | 4.83E-06 | -3.01 |  |  | VPS29-like phosphoesterase-related protein ysnB |  | General function prediction only |
| sa_c8049s7036_at | SA0621 | 2.06E-05 | -2.98 |  |  | conserved hypothetical integral membrane protein |  | General function prediction only |
| sa_c5733s4975_a_at | SA2225 | 0.000123 | -2.78 |  |  | putative phosphoesterase |  | General function prediction only |
| sa_c1671s1408_a_at | SA1167 | 1.49E-05 | -2.72 |  |  | Hydrolase, haloacid dehalogenase-like hydrolase |  | General function prediction only |
| sa_c9864s8601_a_at | SA1690 | 1.05E-05 | -2.67 |  |  | uncharacterized protein recX | *recX* | General function prediction only |
| sa_c5176s4476_a_at | SA2120 | 8.31E-06 | -2.58 |  |  | IAA-Ala hydrolase; IAA-amino acid hydrolase |  | General function prediction only |
| sa_c4914s4219_a_at | SA2054 | 0.00108 | -2.51 |  |  | Predicted permease |  | General function prediction only |
| sa_c4560s3890_a_at | SA1957 | 4.59E-05 | -2.41 |  |  | Hydrolase, haloacid dehalogenase-like hydrolase |  | General function prediction only |
| sa_c8372s7348_a_at | SA0720 | 4.50E-06 | -2.15 |  |  | Predicted P-loop containing kinase, similar to B.subtilis yvcJ |  | General function prediction only |
| sa_c10341s9014_s_at | SA1192 | 0.00205 | -2.07 |  |  | conserved putative membrane protein, possibly a permease |  | General function prediction only |
| sa_c4494s3841_a_at | SA1935 | 0.000158 | -2.04 |  |  | IAA-Ala hydrolase; IAA-amino acid hydrolase | *hmrA* | General function prediction only |
| sa_c3692s3173_at | SA1686 | 4.27E-05 | -2.04 |  |  | hypothetical protein predicted by GeneMark |  | General function prediction only |
| sa_c6720s5861_a_at | SA2470 | 0.00134 | -24.15 |  |  | Histidinol_dh, Histidinol dehydrogenase |  | hypothetical protein |
| sa_c6274s5452_at | SA2359 | 4.37E-06 | -9.17 |  |  | Conserved hypothetical ORF |  | hypothetical protein |
| sa_c5329s4605_a_at | SA2154 | 2.54E-06 | -7.58 |  |  | Conserved hypothetical ORF |  | hypothetical protein |
| sa_c6278s5456_a_at | SA2360 | 1.96E-06 | -7.35 |  |  | hypothetical protein predicted by GeneMark |  | hypothetical protein |
| sa_c6752s5895_a_at | SA2478 | 0.000189 | -6.62 |  |  | 316aa long hypothetical protein |  | hypothetical protein |
| sa_c10265s8943_a_at | SA0335 | 0.000105 | -3.56 |  |  | membrane protein |  | hypothetical protein |
| sa_c7069s6178_a_at | SA0334 | 0.00195 | -3.36 |  |  | Sec-independent protein translocase protein TatC |  | hypothetical protein |
| sa_c6732s5872_a_at | SA2473 | 0.00112 | -3.34 |  |  | Conserved hypothetical ORF |  | hypothetical protein |
| sa_c1944s1667_a_at | SA1233 | 0.000304 | -3.18 |  |  | Conserved hypothetical ORF |  | hypothetical protein |
| sa_i8772ur_x_at | SA1268 | 0.000288 | -3.08 |  |  | reverse complement of intergenic upstream of ORF sa_c10068s8772 | *ebhB* | hypothetical protein |
| sa_c515s343_a_at | SA0888 | 0.00321 | -2.62 |  |  | ABC transporter ATP-binding protein - amino acid transport |  | hypothetical protein |
| sa_c5885s5122_a_at | SA2265 | 0.00193 | -2.60 |  |  | Conserved hypothetical ORF |  | hypothetical protein |
| sa_c507s335_a_at | SA0887 | 0.00137 | -2.56 |  |  | Conserved hypothetical ORF |  | hypothetical protein |
| sa_c2026s1737_a_at | SA1254 | 2.49E-05 | -2.38 |  |  | similar to hypoyhetical protein |  | hypothetical protein |
| sa_c5769s5011_a_at | SA2233 | 0.000987 | -2.36 |  |  | putative efflux membrane protein; putative efflux protein, MFS transporter, DHA2 family, multidrug resistance protein |  | hypothetical protein |
| sa_c9858s8597_a_at | SA1689 | 5.88E-05 | -2.33 |  |  | hypothetical protein predicted by GeneMark |  | hypothetical protein |
| sa_c1860s1583_a_at | SA1210 | 7.65E-05 | -2.31 |  |  | Conserved hypothetical ORF |  | hypothetical protein |
| sa_c8025s7012_a_at | SA0612 | 0.00088 | -2.25 |  |  | Predicted acetyltransferase |  | hypothetical protein |
| sa_c10301s8984_a_at | SA0800 | 0.00225 | -2.25 |  |  | hypothetical protein predicted by GeneMark |  | hypothetical protein |
| sa_c3310s2853_a_at | SA0172 | 0.000127 | -2.25 |  |  | integral membrane protein LmrP |  | hypothetical protein |
| sa_c4892s4201_a_at | SA2049 | 2.84E-05 | -2.17 |  |  | Conserved hypothetical ORF |  | hypothetical protein |
| sa_c7188s10159_s_at | SA0356 | 0.00876 | -2.16 |  |  | integrase |  | hypothetical protein |
| sa_c8147s7131_a_at | SA0648 | 0.000866 | -2.16 |  |  | conserved hypothetical integral membrane protein |  | hypothetical protein |
| sa_c9889s8623_at | SA1743 | 0.00271 | -2.14 |  |  | hypothetical protein predicted by GeneMark |  | hypothetical protein |
| sa_c3459s2987_a_at | SA1613 | 0.00159 | -2.13 |  |  | alpha-hemolysin (hlyA) |  | hypothetical protein |
| sa_c9694s8452_a_at | SA1337 | 0.000539 | -2.12 |  |  | transcription regulator AraC:XylS family homolog |  | hypothetical protein |
| sa_c8143s7126_at | SA0647 | 0.00742 | -2.07 |  |  | Conserved hypothetical ORF |  | hypothetical protein |
| sa_c485s314_at | SA0883 | 0.0185 | -2.04 |  |  | Conserved hypothetical ORF |  | hypothetical protein |
| sa_c5980s5185_a_at | SA2291 | 0.0151 | -2.03 |  |  | Ser-Asp rich fibrinogen-binding bone sialoprotein-binding protein | *fnb* | hypothetical protein |
| sa_c5262s4561_a_at | SA2139 | 0.00378 | -2.02 |  |  | Conserved hypothetical ORF |  | hypothetical protein |
| sa_c7055s6165_a_at | SA0331 | 0.000236 | -3.00 |  |  | probable lipoprotein |  | Inorganic ion transport and metabolism |
| sa_c7061s6172_a_at | SA0332 | 0.000226 | -3.00 |  |  | Hypothetical protein ywbN precursor |  | Inorganic ion transport and metabolism |
| sa_c7063s6174_a_at | SA0333 | 0.00044 | -3.03 |  |  | putative integral membrane protein |  | Inorganic ion transport and metabolism |
| sa_c5418s4689_a_at | SA0420 | 7.55E-05 | -2.84 |  |  | probable amino acid ABC transporter, ATP-binding protein (abc) |  | Inorganic ion transport and metabolism |
| sa_c7374s6406_a_at | SA0421 | 6.63E-07 | -3.34 |  |  | putative amino acid ABC transporter, permease protein, glutamine transport system |  | Inorganic ion transport and metabolism |
| sa_c7945s6932_a_at | SA0585 | 4.77E-05 | -2.87 |  |  | similar to Sodium:hydrogen exchanger 1 (Na(+):H(+) exchanger 1) (NHE-1) (Na+:H+ antiporter, amiloride-sensitive) (APNH) |  | Inorganic ion transport and metabolism |
| sa_c5431s4700_a_at | SA0769 | 2.32E-06 | -5.43 |  |  | probable amino acid ABC transporter, ATP-binding protein (abc) |  | Inorganic ion transport and metabolism |
| sa_c8512s7471_a_at | SA0770 | 1.42E-05 | -6.33 |  |  | permease protein of ABC transporter system |  | Inorganic ion transport and metabolism |
| sa_c8518s7475_a_at | SA0771 | 5.12E-06 | -7.52 |  |  | Probable D-methionine-binding lipoprotein metQ precursor (Outer membrane lipoprotein 1) |  | Inorganic ion transport and metabolism |
| sa_c9012s7913_a_at | SA0780 | 9.93E-05 | -2.54 |  |  | TlyC; hemolysin |  | Inorganic ion transport and metabolism |
| sa_c439s269_a_at | SA0872 | 0.000309 | -2.43 |  |  | hypothetical protein predicted by GeneMark |  | Inorganic ion transport and metabolism |
| sa_i622d_x_at | SA0956 | 5.60E-05 | -2.41 |  |  | intergenic downstream of ORF sa_c820s622 |  | Inorganic ion transport and metabolism |
| sa_c4110s3463_a_at | SA1815 | 0.000211 | -2.82 |  |  | hypothetical protein, similar to Na+-transporting ATP synthase |  | Inorganic ion transport and metabolism |
| sa_c5500s4763_a_at | SA2166 | 0.000568 | -2.38 |  |  | hypothetical protein, simialr to cationic transporter |  | Inorganic ion transport and metabolism |
| sa_c5739s4980_a_at | SA2228 | 0.000903 | -2.12 |  |  | probable sodium:hydrogen antiporter |  | Inorganic ion transport and metabolism |
| sa_c6212s5389_a_at | SA2344 | 1.84E-05 | -3.60 |  |  | Cu-transporting P1-type ATPase; copper resistance determinant 1; Crd1p; CPx-type ATPase; type I ATPase | *copA* | Inorganic ion transport and metabolism |
| sa_c6740s5882_a_at | SA2475 | 0.000217 | -6.58 |  |  | ABC transporter membrane-spanning permease - unknown substrate |  | Inorganic ion transport and metabolism |
| sa_c5397s4673_a_at | SA2476 | 0.000459 | -7.63 |  |  | putative ABC transporter; ATP-binding protein; possible cobalt transport system |  | Inorganic ion transport and metabolism |
| sa_c5829s5070_a_at | SA0241 | 0.000321 | -2.04 |  |  | 2-C-methyl-D-erythritol 4-phosphate cytidylyltransferase (4-diphosphocytidyl-2C-methyl-D-erythritol synthase) (MEP cytidylyltransferase) (MCT) (CDP-ME synthetase) | *ispD* | Lipid metabolism |
| sa_c4492s3837_a_at | SA1934 | 0.000731 | -2.06 |  |  | Conserved hypothetical ORF |  | Lipid metabolism, Amino acid transport and metabolism, Nucleotide transport and metabolism |
| sa_c483s311_a_at | SA0881 | 0.000223 | -2.69 |  |  | 5-nucleotidase:2,3-cyclic phosphodiesterase related enzyme |  | Nucleotide transport and metabolism |
| sa_c8497s7459_a_at | SA0758 | 0.000517 | -2.52 |  |  | thiored, Thioredoxin |  | Posttranslational modification, protein turnover, chaperones, Energy production and conversion |
| sa_c8637s7585_a_at | SA0805 | 0.000646 | -3.61 |  |  | ComA operon protein 2 |  | Secondary metabolites biosynthesis, transport and catabolism |
| sa_c10346s10731_s_at | SA0173 | 0.000872 | -2.08 |  |  | hypothetical protein, similar to surfactin synthetase |  | Secondary metabolites biosynthesis, transport and catabolism |
| sa_c9305s8148_a_at | SA2105 | 3.65E-05 | -3.21 |  |  | HTH_DEOR, helix_turn_helix, Deoxyribose operon repressor |  | Transcription |
| sa_c6424s5594_a_at | SA2407 | 0.00202 | -2.72 |  |  | putative regulatory protein |  | Transcription |
| sa_c4939s4249_a_at | SA2062 | 0.00136 | -2.36 |  |  | staphylococcal accessory regulator A homolog |  | Transcription |
| sa_c3365s2900_a_at | SA1583 | 0.00259 | -2.11 |  |  | repressor of toxins Rot | *rot* | Transcription |
| sa_c3252s2802_a_at | SA1557 | 8.75E-05 | -2.08 |  |  | Transcriptional regulator, LacI family (probably maltose operon transcriptional repressor) | *ccpA* | Transcription |
| sa_c5323s4600_a_at | SA2153 | 2.22E-06 | -6.99 |  |  | weakly similar to two-component response regulator |  | Transcription, Signal transduction mechanisms |
| sa_c6738s5878_a_at | SA2474 | 0.000247 | -4.74 |  |  | ribosomal-protein-alanine acetyltransferase |  | Translation, ribosomal structure and biogenesis |
| sa_c8966s7880_a_at | SA0707 | 0.012 | -2.02 |  |  | Ribosomal_S30, Sigma 54 modulation protein : S30EA ribosomal protein |  | Translation, ribosomal structure and biogenesis |
|  |  |  |  |  |  |  |  |  |
| **Group VI: No change (20 min) - Downregulation (60min) 116 genes** | | | | | | |  |  |
| sa_c9525s8305_a_at | SA0112 |  |  | 0.00727 | -5.62 | Cysteine synthase (O-acetylserine sulfhydrylase) (O-acetylserine (Thiol)-lyase) (CSase) |  | Amino acid transport and metabolism |
| sa_c1308s1080_a_at | SA0113 |  |  | 1.60E-05 | -3.66 | ornithine cyclodeaminase; Ocd1 |  | Amino acid transport and metabolism |
| sa_c9193s8056_at | SA0313 |  |  | 0.000259 | -2.42 | glycine cleavage system protein H precursor (clone HFC2) |  | Amino acid transport and metabolism |
| sa_c388s229_a_at | SA0859 |  |  | 0.000262 | -2.01 | thimet oligopeptidase homologue |  | Amino acid transport and metabolism |
| sa_c10653s11106_s_at | SA1360 |  |  | 8.98E-05 | -2.00 | Xaa-Pro dipeptidase (X-Pro dipeptidase) (Proline dipeptidase) (Prolidase) (Imidodipeptidase) |  | Amino acid transport and metabolism |
| sa_c3150s2697_a_at | SA1531 |  |  | 0.0113 | -2.44 | AlaDh_PNT, Alanine dehydrogenase:pyridine nucleotide transhydrogenase | *ald* | Amino acid transport and metabolism |
| sa_c5195s4497_a_at | SA2125 |  |  | 0.00254 | -2.76 | Formimidoylglutamase (Formiminoglutamase) (Formiminoglutamate hydrolase) |  | Amino acid transport and metabolism |
| sa_c1447s1221_a_at | SA0118 |  |  | 6.09E-05 | -2.25 | 2-dehydro-3-deoxyglucarate aldolase (2-keto-3-deoxyglucarate aldolase) (2-dehydro-3-deoxygalactarate aldolase) (DDG aldolase) (5-keto-4-deoxy-D-glucarate aldolase) (KDGlucA) |  | Carbohydrate transport and metabolism |
| sa_c3850s3320_a_at | SA0186 |  |  | 0.0384 | -3.60 | PTS SYSTEM, SUCROSE-SPECIFIC IIBC COMPONENT (EIIBC-SCR) (SUCROSE- PERMEASE IIBC COMPONENT) (PHOSPHOTRANSFERASE ENZYME II, BC COMPONENT) (EC 2.7.1.69) (EII-SCR). |  | Carbohydrate transport and metabolism |
| sa_c7211s6279_a_at | SA0361 |  |  | 0.00168 | -2.30 | 6-Phosphofructo-2-kinase:fructose-2,6-bisphosphatase 2 (heart) |  | Carbohydrate transport and metabolism |
| sa_c8003s6987_a_at | SA0606 |  |  | 0.000468 | -4.15 | Dihydroxyacetone kinase 2 (Glycerone kinase 2) (DHA kinase 2) |  | Carbohydrate transport and metabolism |
| sa_c8169s7149_a_at | SA0654 |  |  | 0.00226 | -3.06 | Fructose-1-phosphate kinase and related fructose-6-phosphate kinase (PfkB) | *fruB* | Carbohydrate transport and metabolism |
| sa_c10615s11069cv_s_at | SA0655 |  |  | 0.00134 | -6.62 | PTS system, fructose-specific IIBC component (EIIBC-Fru) (Fructose-permease IIBC component) (Phosphotransferase enzyme II, BC component) (EII-Fru) | *fruA* | Carbohydrate transport and metabolism, Signal transduction mechanisms |
| sa_c8176s7154_a_at | SA0656 |  |  | 0.000304 | -2.22 | N-ACETYLGLUCOSAMINE-6-PHOSPHATE DEACETYLASE (GLCNAC 6-P DEACETYLASE) | *nagA* | Carbohydrate transport and metabolism |
| sa_c8689s7638_a_at | SA0823 |  |  | 7.40E-05 | -2.01 | glucose phosphate isomerase 1; glucose phosphate isomerase 1 complex; glucose phosphate isomerase 1, temporal; glucose phosphate isomerase 1, regulatory; glucose phosphate isomerase 1, structural | *pgi* | Carbohydrate transport and metabolism |
| sa_c3059s2614_a_at | SA1510 |  |  | 0.0337 | -2.89 | Glyceraldehyde 3-phosphate dehydrogenase 2 (GAPDH) (NAD(P)-dependent glyceraldehyde-3-phosphate dehydrogenase) | *gapB* | Carbohydrate transport and metabolism |
| sa_c5968s5174_a_at | SA2279 |  |  | 0.00406 | -2.56 | two functions are possible, as both enzymes are homologous to each other: phosphomannomutase or phosphoglucomutase |  | Carbohydrate transport and metabolism |
| sa_c6391s5562_a_at | SA2399 |  |  | 2.50E-05 | -2.34 | aldolase A; fructose-bisphosphate aldolase; Aldolase A, fructose-bisphosphatase |  | Carbohydrate transport and metabolism |
| sa_c2385s1987_a_at | SA0145 |  |  | 4.55E-06 | -2.90 | capsular polysaccharide synthesis enzyme Cap5B | *capB* | Cell division and chromosome partitioning |
| sa_c6119s5300_a_at | SA0249 |  |  | 0.0199 | -2.82 | cell division and morphogenesis-related protein | *scdA* | Cell division and chromosome partitioning |
| sa_c5616s4872_a_at | SA2196 |  |  | 0.00569 | -2.47 | Conserved hypothetical ORF |  | Cell division and chromosome partitioning |
| sa_c2346s1974_a_at | SA0144 |  |  | 4.02E-05 | -3.08 | capsular polysaccharide synthesis enzyme Cap5A | *capA* | Cell envelope biogenesis, outer membrane |
| sa_c2399s1991_a_at | SA0146 |  |  | 0.000551 | -2.62 | capsular polysaccharide synthesis enzyme Cap8C | *capC* | Cell envelope biogenesis, outer membrane, Carbohydrate transport and metabolism |
| sa_c2413s1997_a_at | SA0147 |  |  | 0.000102 | -2.58 | capsular polysaccharide synthesis enzyme Cap5D | *capD* | Cell envelope biogenesis, outer membrane, Carbohydrate transport and metabolism |
| sa_c9546s8318_a_at | SA0148 |  |  | 9.77E-05 | -2.77 | Putative UDP-glucose 4-epimerase (Galactowaldenase) (UDP-galactose 4-epimerase) | *capE* | Cell envelope biogenesis, outer membrane, Carbohydrate transport and metabolism |
| sa_c2479s2056_a_at | SA0149 |  |  | 2.40E-06 | -3.13 | capsular polysaccharide synthesis enzyme Cap5F | *capF* | Cell envelope biogenesis, outer membrane, Carbohydrate transport and metabolism |
| sa_c2516s2092_a_at | SA0150 |  |  | 5.92E-05 | -2.40 | UDP-N-acetylglucosamine 2-epimerase (UDP-GlcNAc-2-epimerase) | *capG* | Cell envelope biogenesis, outer membrane |
| sa_c10087s8814_a_at | SA0152 |  |  | 0.00017 | -2.68 | capsular polysaccharide synthesis enzyme Cap5I | *capI* | Cell envelope biogenesis, outer membrane |
| sa_c10089s8822_a_at | SA0154 |  |  | 0.00103 | -2.14 | capsular polysaccharide synthesis enzyme Cap5K | *capK* | Cell envelope biogenesis, outer membrane |
| sa_c7509s6527_a_at | SA0457 |  |  | 0.00173 | -2.04 | UDP-N-ACETYLGLUCOSAMINE PYROPHOSPHORYLASE (N-ACETYLGLUCOSAMINE-1-PHOSPHATE URIDYLTRANSFERASE) | *gcaD* | Cell envelope biogenesis, outer membrane |
| sa_c7706s6706_a_at | SA0522 |  |  | 0.000146 | -2.43 | hypothetical protein, similar to poly (glycerol-phosphate) alpha-glucosyltransferase (teichoic acid biosynthesis) |  | Cell envelope biogenesis, outer membrane |
| sa_c898s698_a_at | SA0977 |  |  | 0.000443 | -2.74 | 29-kDa cell surface protein | *isdA* | Cell envelope biogenesis, outer membrane |
| sa_c906s704_a_at | SA0978 |  |  | 0.00538 | -2.15 | hypothetical protein SirD | *isdC* | Cell envelope biogenesis, outer membrane |
| sa_c2246s1944_a_at | SA1312 |  |  | 8.78E-05 | -2.17 | cell surface elastin binding protein | *ebpS* | Cell envelope biogenesis, outer membrane |
| sa_c5757s4999_a_at | SA2231 |  |  | 0.00301 | -2.55 | UDP-glucose 4-epimerase |  | Cell envelope biogenesis, outer membrane, Carbohydrate transport and metabolism |
| sa_c3380s9339_a_at | SA1586 |  |  | 1.41E-05 | -2.41 | 6,7-dimethyl-8-ribityllumazine synthase (DMRL synthase) (Lumazine synthase) (Riboflavin synthase beta chain) | *ribH* | Coenzyme metabolism |
| sa_c3387s2918_a_at | SA1587 |  |  | 6.15E-06 | -2.75 | PROBABLE RIBOFLAVIN BIOSYNTHESIS BIFUNCTIONAL PROTEIN : GTP CYCLOHYDROLASE II AND 3,4-DIHYDROXY-2-BUTANONE-4-PHOSPHATE SYNTHASE (DHBP SYNTHASE) | *ribA* | Coenzyme metabolism |
| sa_c3391s2919_a_at | SA1588 |  |  | 3.10E-06 | -2.67 | riboflavin synthase alpha chain | *ribB* | Coenzyme metabolism |
| sa_c3395s2925_a_at | SA1589 |  |  | 5.10E-07 | -3.01 | riboflavin specific deaminase(diaminohydroxyphosphoribosylaminopyrimidine deaminase:5-amino-6-(5-phosphoribosylamino)uracil reductase); riboflavin specific deaminase | *ribD* | Coenzyme metabolism |
| sa_c4369s3721_at | SA1894 |  |  | 0.000661 | -2.27 | Thiamine-phosphate pyrophosphorylase (TMP pyrophosphorylase) (TMP-PPase) (Thiamine-phosphate synthase) | *thiE* | Coenzyme metabolism |
| sa_c4373s3725_a_at | SA1895 |  |  | 3.05E-05 | -2.53 | Hydroxyethylthiazole kinase (4-methyl-5-beta-hydroxyethylthiazole kinase) (Thz kinase) (TH kinase) | *thiM* | Coenzyme metabolism |
| sa_c4379s3726_a_at | SA1896 |  |  | 0.000137 | -2.79 | bifunctional enzyme: hydroxy-phosphomethylpyrimidine kinase (HMP-P kinase); hydroxy-methylpyrimidine kinase (HMP kinase) | *thiD* | Coenzyme metabolism |
| sa_c4397s3747_at | SA1899 |  |  | 0.000382 | -2.45 | single-strand DNA-binding protein (ssb) |  | DNA replication, recombination and repair |
| sa_c943s737_a_at | SA0987 |  |  | 0.00169 | -2.36 | Ribonuclease HIII (RNase HIII) |  | DNA replication, recombination and repair |
| sa_c6988s6111_a_at | SA0312 |  |  | 0.000501 | -2.83 | Alkanal monooxygenase alpha chain (Bacterial luciferase alpha chain) |  | Energy production and conversion |
| sa_c7841s6831_a_at | SA0557 |  |  | 5.05E-05 | -2.39 | putative aldo:keto reductase; putative oxidoreductase (fragment) |  | Energy production and conversion |
| sa_c1988s9104_a_at | SA1244 |  |  | 0.00134 | -2.76 | Dihydrolipoamide succinyltransferase component of 2-oxoglutarate dehydrogenase complex (E2) | *odhB* | Energy production and conversion |
| sa_c1994s9145_a_at | SA1245 |  |  | 0.00024 | -3.92 | oxoglutarate (alpha-ketoglutarate) dehydrogenase (lipoamide); oxoglutarate dehydrogenase (lipoamide) | *kgd* | Energy production and conversion |
| sa_c2446s2028_a_at | SA1331 |  |  | 9.29E-05 | -3.11 | putative aldo:keto reductase; putative oxidoreductase (fragment) |  | Energy production and conversion |
| sa_c3448s2976_a_at | SA1609 |  |  | 0.0011 | -5.41 | Phosphoenolpyruvate carboxykinase [ATP] (PEP carboxykinase) (Phosphoenolpyruvate carboxylase) (PEPCK) | *pckA* | Energy production and conversion |
| sa_c5464s4730_a_at | SA2160 |  |  | 0.00407 | -2.06 | dehydrogenase (zinc-binding alcohol dehydrogenase, NADPH quinone oxidoreductase, oxidoreductase) |  | Energy production and conversion |
| sa_c8005s6991_at | SA0607 |  |  | 0.000204 | -4.13 | probable PTS system enzyme I ycgC |  | Function unknown |
| sa_c939s733_a_at | SA0102 |  |  | 0.046 | -3.17 | 67 kDa Myosin-crossreactive streptococcal antigen homologue |  | Function unknown |
| sa_c2109s1814_a_at | SA1275 |  |  | 2.14E-05 | -2.65 | putative integral membrane protein |  | Function unknown |
| sa_c1796s1526_a_at | SA1193 |  |  | 0.000856 | -2.49 | oxacillin resistance-related FmtC protein | *fmtC* | Function unknown |
| sa_c2487s2066_a_at | SA1344 |  |  | 0.00315 | -2.44 | Predicted membrane, YQJA B.subtilis ortholog |  | Function unknown |
| sa_c5081s4376_a_at | SA2096 |  |  | 0.0248 | -2.26 | Conserved hypothetical ORF |  | Function unknown |
| sa_c5614s4868_a_at | SA2195 |  |  | 0.00309 | -2.21 | AviX3 |  | Function unknown |
| sa_c611s432_a_at | SA0909 |  |  | 8.53E-06 | -2.08 | Fmt, autolysis and methicillin resistant-related protein | *fmtA* | Function unknown |
| sa_c7199s6265_a_at | SA0359 |  |  | 0.00113 | -2.03 | Conserved hypothetical ORF |  | Function unknown |
| sa_c3814s3284_a_at | SA0185 |  |  | 0.0121 | -4.72 | GLUCOKINASE REGULATORY PROTEIN (GLUCOKINASE REGULATOR) | *murQ* | General function prediction only |
| sa_c10086s8810_a_at | SA0151 |  |  | 0.000248 | -2.77 | Chloramphenicol acetyltransferase (Xenobiotic acetyltransferase) (XAT) | *capH* | General function prediction only |
| sa_c6077s5267_a_at | SA2313 |  |  | 0.0188 | -2.36 | Hydrolase, haloacid dehalogenase-like hydrolase |  | General function prediction only |
| sa_c7895s6886_a_at | SA0572 |  |  | 0.0142 | -2.09 | 3-oxoadipate enol-lactonase I (Enol-lactone hydrolase I) (Beta-ketoadipate enol-lactone hydrolase I) |  | General function prediction only |
| sa_c6647s5802_a_at | SA2452 |  |  | 4.05E-05 | -2.00 | AGR_pAT_605p |  | General function prediction only |
| sa_c6626s5782_a_at | SA2447 |  |  | 7.40E-06 | -4.44 | serine-threonine rich antigen |  | hypothetical protein |
| sa_c6597s5767_a_at | SA2445 |  |  | 6.21E-06 | -4.17 | Conserved hypothetical ORF |  | hypothetical protein |
| sa_c3778s3248_a_at | SA0184 |  |  | 0.0374 | -3.70 | similar to putative outer surface protein |  | hypothetical protein |
| sa_c4755s4067_a_at | SA2006 |  |  | 0.00719 | -3.38 | hypothetical protein, similar to MHC class II analog |  | hypothetical protein |
| sa_c6591s5758_a_at | SA2443 |  |  | 2.31E-05 | -3.29 | Conserved hypothetical ORF |  | hypothetical protein |
| sa_c6603s5771_a_at | SA2446 |  |  | 0.000476 | -3.29 | PREPROTEIN TRANSLOCASE SECY SUBUNIT (secY) | *secY* | hypothetical protein |
| sa_c1340s1111_a_at | SA0114 |  |  | 0.0178 | -3.07 | probable siderophore biosynthesis protein IucC iucC |  | hypothetical protein |
| sa_c5717s4960_a_at | SA2220 |  |  | 0.000111 | -2.75 | putative glycerate kinase |  | hypothetical protein |
| sa_c1388s1161_a_at | SA0116 |  |  | 0.000178 | -2.72 | hypothetical protein, similar to rhizobactin siderophore biosynthesisprotein RhsC |  | hypothetical protein |
| sa_c5507s10021cs_s_at | SA2168 |  |  | 0.00208 | -2.62 | probable type II DNA modification enzyme |  | hypothetical protein |
| sa_c5470s4736_a_at | SA2161 |  |  | 0.000151 | -2.58 | hypothetical protein, similar to attachment to host cells and virulence |  | hypothetical protein |
| sa_c9309s8152_at | SA2113 |  |  | 0.00591 | -2.47 | Conserved hypothetical ORF |  | hypothetical protein |
| sa_c2489s2070_at | SA1345 |  |  | 0.006 | -2.44 | hypothetical protein predicted by GeneMark |  | hypothetical protein |
| sa_c1358s1133_a_at | SA0115 |  |  | 0.000182 | -2.44 | putative permease; possible multi-drug resistance efflux pump |  | hypothetical protein |
| sa_c304s144_a_at | SA0841 |  |  | 5.72E-05 | -2.36 | hypothetical protein, similar to cell surface protein Map-w |  | hypothetical protein |
| sa_c5510s4767_a_at | SA2169 |  |  | 8.67E-05 | -2.32 | hypothetical protein, simialr to transcription regulatory protein |  | hypothetical protein |
| sa_c1310s1083_a_at | SA1085 |  |  | 7.67E-05 | -2.25 | Conserved hypothetical ORF |  | hypothetical protein |
| sa_i2032d_x_at | SA1332 |  |  | 0.000478 | -2.19 | intergenic downstream of ORF sa_c2448s2032 |  | hypothetical protein |
| sa_c8807s7748_a_at | SA0480 |  |  | 0.0162 | -2.16 | transcription repressor of class III stress genes ctsR |  | hypothetical protein |
| sa_c4655s3976_a_at | SA1981 |  |  | 2.90E-05 | -2.10 | probable siderophore biosynthesis protein IucC iucC |  | hypothetical protein |
| sa_c6994s6115_a_at | SA0314 |  |  | 0.000113 | -2.08 | Hypothetical 29.1 kDa protein in tap1-dppD intergenic region |  | hypothetical protein |
| sa_c6333s5501_a_at | SA2376 |  |  | 0.00125 | -2.06 | Conserved hypothetical ORF |  | hypothetical protein |
| sa_c1723s1460_a_at | SA1179 |  |  | 0.018 | -2.00 | CcdC protein |  | hypothetical protein |
| sa_c10690s11140_s_at | SA2486 |  |  | 0.000737 | -2.98 | oxoglutarate:malate translocator precursor-like protein |  | Inorganic ion transport and metabolism |
| sa_c1230s1008_at | SA0111 |  |  | 0.00096 | -2.16 | iron(III) ABC transporter, periplasmic iron-compound-binding protein | *sirA* | Inorganic ion transport and metabolism |
| sa_c2560s2136_at | SA1364 |  |  | 0.000411 | -2.04 | Thiosulfate sulfurtransferase glpE |  | Inorganic ion transport and metabolism |
| sa_c4786s4095_a_at | SA0223 |  |  | 0.00862 | -5.26 | acetyl-CoA acyltransferase, 3-oxo acyl-CoA thiolase A, peroxisomal; Acetyl-CoA acyltransferase, 3-oxo acyl-CoA thiolase A 1, peroxisomal |  | Lipid metabolism |
| sa_c7092s6204_a_at | SA0342 |  |  | 0.000207 | -3.29 | 3-ketoacyl-CoA thiolase, peroxisomal precursor (Beta-ketothiolase) (Acetyl-CoA acyltransferase) (Peroxisomal 3-oxoacyl-CoA thiolase) |  | Lipid metabolism |
| sa_c6163s5345_a_at | SA2334 |  |  | 8.79E-05 | -2.46 | Hydroxymethylglutaryl-CoA synthase (HMG-CoA synthase) (3-hydroxy-3-methylglutaryl coenzyme A synthase) | *mvaS* | Lipid metabolism |
| sa_c1624s9141_a_at | SA1155 |  |  | 0.0146 | -2.20 | Probable cardiolipin synthetase 2 (Cardiolipin synthase 2) (CL synthase 2) |  | Lipid metabolism |
| sa_c9318s8160_a_at | SA2140 |  |  | 0.029 | -2.03 | putative esterase:acetyl hydrolase |  | Lipid metabolism |
| sa_c2735s2309_at | SA1417 |  |  | 0.00187 | -2.02 | late competence operon required for DNA binding and uptake comEB |  | Nucleotide transport and metabolism |
| sa_c2977s2534_a_at | SA1489 |  |  | 0.0101 | -2.56 | DNA-3-methyladenine glycosylase I (3-methyladenine-DNA glycosylase I, constitutive) (TAG I) (DNA-3-methyladenine glycosidase I) | *tag* | Nucleotide transport and metabolism |
| sa_c3237s2785_a_at | SA1553 |  |  | 0.00131 | -3.73 | methylenetetrahydrofolate dehydrogenase (NADP+ dependent), methenyltetrahydrofolate cyclohydrolase, formyltetrahydrofolate synthetase | *fhs* | Nucleotide transport and metabolism |
| sa_c4619s3943_a_at | SA0219 |  |  | 0.00935 | -4.20 | Pyruvate formate-lyase 2 activating enzyme (PFL-activating enzyme) | *pflA* | Posttranslational modification, protein turnover, chaperones |
| sa_c8485s7447_a_at | SA0755 |  |  | 0.0403 | -2.23 | Organic hydroperoxide resistance protein ohrB (General stress protein 17o) (Gsp17o) |  | Posttranslational modification, protein turnover, chaperones |
| sa_c278s123_a_at | SA0835 |  |  | 0.0426 | -2.00 | ATP-dependent protease ATP-binding subunit (fragment); putative ATP-dependent protease ATP-binding subunit | *clpB* | Posttranslational modification, protein turnover, chaperones |
| sa_c732s540_a_at | SA0936 |  |  | 0.0129 | -2.37 | putative NrdH-redoxin |  | Posttranslational modification, protein turnover, chaperones |
| sa_c1518s1296_a_at | SA1146 |  |  | 0.000556 | -2.61 | glutathione peroxidase 4; sperm nuclei glutathione peroxidase; phospholipid hydroperoxide glutathione peroxidase; 1700027O09Rik |  | Posttranslational modification, protein turnover, chaperones |
| sa_c2243s1939_a_at | SA1311 |  |  | 0.000575 | -2.07 | Thioredoxin reductase (TRXR) |  | Posttranslational modification, protein turnover, chaperones |
| sa_c5043s4344_a_at | SA2087 |  |  | 0.000115 | -2.53 | urease accessory protein UreG | *ureG* | Posttranslational modification, protein turnover, chaperones, Transcription |
| sa_c6449s5616_a_at | SA0257 |  |  | 0.0491 | -2.02 | Probable sterol 24-C-methyltransferase (Delta(24)-sterol C-methyltransferase) |  | Secondary metabolites biosynthesis, transport and catabolism |
| sa_c3928s3395_a_at | SA0187 |  |  | 0.0331 | -3.27 | transcriptional regulator (hex regulon repressor) |  | Transcription |
| sa_c6998s6118_a_at | SA0315 |  |  | 0.000561 | -2.00 | unknown; predicted coding region |  | Transcription |
| sa_c8164s7148_a_at | SA0653 |  |  | 0.000831 | -2.43 | putative DEOR-type transcriptional regulator of aga operon |  | Transcription, Carbohydrate transport and metabolism |
| sa_c9781s8524_a_at | SA1195 |  |  | 6.95E-05 | -2.58 | peptide methionine sulfoxide reductase regulator MsrR | *msrR* | Transcription |
| sa_c4381s3732_a_at | SA1897 |  |  | 0.00359 | -2.40 | hypothetical protein, simialr to trasncriptional activator TenA |  | Transcription |
| sa_c4577s3908_a_at | SA1961 |  |  | 0.00208 | -2.90 | putative transcriptional regulator; possible antiterminator |  | Transcription |
| sa_c6003s5203_a_at | SA2296 |  |  | 8.73E-06 | -4.18 | putative transcriptional activator regulator protein |  | Transcription |
| sa_c2160s1859_a_at | SA1287 |  |  | 0.00015 | -2.06 | Asparaginyl-tRNA synthetase (Asparagine--tRNA ligase) (AsnRS) | *asnC* | Translation, ribosomal structure and biogenesis |
| sa_c1691s1429_x_at | SA1171 |  |  | 0.000572 | -2.03 | 30S ribosomal protein S14; ribosomal protein S14 |  | Translation, ribosomal structure and biogenesis |
